# The epidemiological trends of multiple sclerosis among women of child-bearing age: a global analysis from 1990 to 2021 and forecasts to 2040

**DOI:** 10.3389/fimmu.2026.1677178

**Published:** 2026-05-01

**Authors:** Zhuo-lei Cai, Fei Dong, Jiachen Fan, Yuan-min Chang, Jin-yu Ma, Dujun Wang, Wenmin Yi, Zhi-you Cai, Lei Cao

**Affiliations:** 1 Department of Neurology, The Second Affiliated Hospital of Anhui Medical University, Hefei, Anhui, China; 2 Department of Neurology, The First Affiliated Hospital of Zhengzhou University, Zhengzhou, Henan, China; 3 People’s Hospital of Zhengzhou University, Zhengzhou, Henan, China; 4 International Medical Department, The Second Affiliated Hospital of Anhui Medical University, Hefei, Anhui, China; 5 Clinical College of Anhui Medical University, Hefei, Anhui, China; 6 The Discipline Construction and Development Fund of Liangjiang New Area People’s Hospital, Chongqing Medical University, Chongqing, China; 7 Liangjiang New Area People’s Hospital, Chongqing Medical University, Chongqing, China

**Keywords:** disability adjusted life year (DALYs), forecasts analysis, global burden of disease study, incidence, mortality, multiple sclerosis, women of child-bearing age

## Abstract

**Introduction:**

Multiple sclerosis (MS) shows marked female predominance, particularly among women of childbearing age (WCBA, 15–49 years), yet their specific disease burden remains under characterized. Using Global Burden of Disease (GBD) 1990–2021 data, we conducted the first comprehensive analysis of MS trends in WCBA globally, with projections to 2040, to guide targeted interventions for this vulnerable population.

**Methods:**

We analyzed age-standardized incidence (ASIR), prevalence (ASPR), mortality (ASMR), and disability-adjusted life years (ASDR) rates across 204 countries using GBD 2021 data. Our analytical approach incorporated Pearson correlation to examine Socio-demographic Index (SDI) relationships, decomposition analysis to identify burden drivers, and frontier analysis to assess disease control efficiency gaps. We employed Bayesian Age-Period-Cohort modeling alongside ARIMA for 20-year projections, with joinpoint regression analyzing temporal trends through annual percentage changes. Age-period-cohort effects were further disentangled through APC modeling, with stratification by age group, SDI level, and geographic region.

**Results:**

Between 1990-2021, WCBA experienced a 48% rise in MS incidence and 66% increase in prevalence, with mortality growing by 17% despite a 7% DALYs reduction. Age-standardized rates improved (ASMR:-0.35; ASDR:-0.3), though burden varied regionally: Australasia showed greatest incidence growth versus East Asia’s decline, while Central Latin America led prevalence increases. High-SDI countries (>0.4) carried disproportionate burden, peaking at ages 45-49 (incidence:30–34 years). Projections indicate rising crude rates but falling ASDR. Low-SDI nations (e.g., Cambodia) demonstrated optimal control versus high-SDI underperformers (e.g., Sweden). Birth cohorts revealed generally declining risk (RR1.146→0.805) except 2002-2006(RR1.413), with elevated DALYs(20-25y) and prevalence(30-35y) warranting targeted action.

**Discussion:**

This study reveals key disparities in MS burden among WCBA, with high-SDI countries showing paradoxical high burden yet improvement potential. The 2002–2006 cohort rebound and persistent burdens in 30-49-year-olds highlight critical intervention windows. While low-SDI countries demonstrate unexpected control efficiency, rising crude rates globally underscore the growing women’s health challenge of MS, particularly given projected prevalence increases. These findings advocate for tailored, age-specific management strategies.

## 1 Introduction

MS, a chronic autoimmune inflammatory demyelinating disorder of the central nervous system, represents one of the most prevalent neurological conditions affecting young to middle-aged individuals (1, 2). Characterized by progressive motor, sensory, and cognitive dysfunction, MS imposes substantial global disease burden and results in significant increase in disability-adjusted life years (DALYs),as evidenced by GBD studies. The global prevalence of MS has been increasing steadily since 1990, with marked regional differences in frequency. In 2023, it reached 2.9 million. The findings will aid in resource allocation and planning of health services. However, epidemiological data on MS are lacking or unavailable in many parts of the world, and more research is needed to make more accurate estimates (1–3). While high-income nations bear the greatest disease burden, recent epidemiological surveillance reveals increasing incidence rates in low- and middle-income countries (LMICs) (3), likely attributable to enhanced diagnostic capabilities and evolving environmental risk factors. The condition’s impact extends beyond clinical manifestations, generating considerable socio-economic consequences through direct medical expenditures, productivity losses, and long-term care requirements (3–7). Research indicates that due to hormonal influences and environmental factors, MS disproportionately affects women, with a significantly higher prevalence compared to men. While MS does not substantially impair biological fertility (8), it may reduce effective fertility through fatigue, sexual dysfunction, and altered reproductive decision-making (9). Understanding the epidemiological features identified in this study—particularly the peak incidence at 30–34 years and the rising burden in early adulthood—is critical in this context, as it highlights the direct overlap between MS onset and the prime reproductive window, underscoring the need for timely diagnosis and preconception disease control to preserve fertility options and pregnancy outcomes.

Women of childbearing age (WCBA, 15–49 years) represent a high-risk population for MS, with the peak incidence overlapping significantly with reproductive years (10, 11). Research suggests that sex hormones (e.g., estrogens) may modulate MS disease activity—for instance, pregnancy is often associated with clinical remission and may even improve long-term prognosis, though it increases the risk of postpartum relapse (12, 13). Beyond health implications, MS impacts WCBA in broader dimensions, including fertility decisions, treatment choices (e.g., safety of disease-modifying therapies [DMTs]) (14), and psychosocial stressors (10, 11, 13). However, most current epidemiological studies on MS do not specifically analyze the WCBA subgroup, resulting in insufficient evaluation of this critical population’s disease burden and long-term healthcare management needs.

Previous GBD studies have systematically analyzed worldwide epidemiological trends in MS, reporting variations in incidence, prevalence, and DALYs across regions, age groups, and sexes (3–6, 15, 16). However, these studies typically treat MS as a homogeneous entity without in-depth exploration of disease patterns in specific subgroups, such as WCBA. Furthermore, existing MS prediction models are largely based on limited time spans or localized data, potentially failing to accurately capture long-term global trends—particularly the unique epidemiological characteristics of the WCBA population. Consequently, there is an urgent need for a dedicated assessment of the MS disease burden in WCBA to address this gap in the literature and inform targeted public health interventions. Leveraging GBD data from 1990 to 2021, this study aims to systematically evaluate the MS disease burden among women of childbearing age (WCBA) worldwide, analyze its spatiotemporal trends, and develop a predictive model to estimate future disease burden.

## 2 Materials and methods

### 2.1 Data source and collection

All data in this study were sourced from the GBD 2021 study (accessible via https://ghdx.healthdata.org/gbd-results-tool). Our analysis specifically focuses on MS burden among women of childbearing age (WCBA, defined as 15–49 years (17)), encompassing prevalence, incidence, mortality, and DALYs with their corresponding 95% uncertainty intervals (UIs). These metrics were stratified by geographic region, nation, age group, and Socio-demographic Index (SDI) quintiles.

GBD 2021 study employed standardized methodologies to evaluate 371 diseases and injuries across 204 countries and regions, incorporating analysis of 88 demographic-specific risk factors (18). Specifically, GBD collects raw data through systematic searches of published literature, government health statistical reports, hospital discharge records, and vital registration systems across countries and regions. After cross-walking case definitions and standardizing coding schemes, the data are modeled using the Bayesian meta-regression tool DisMod-MR 2.1. For low-income regions with sparse data, the model generates estimates by incorporating covariates such as the Socio-demographic Index and latitude, while borrowing strength from neighboring geographic areas. Finally, a cause-of-death ensemble calibration ensures internal consistency across all data sources. This WHO-IHME collaborative initiative with >1,000 global researchers calculates key metrics - including mortality, morbidity and DALYs - to map disease distributions and risk factor impact (19, 20).Our study utilized GHDx-derived data, processed using R software (*p* < 0.05 significance threshold). The study complies with GATHER reporting guidelines (19).

### 2.2 Descriptive analysis

To assess the global impact of MS among WCBA (15–49 years), we performed a multi-level epidemiological analysis using 2021 data. The study quantified disease burden through absolute metrics (cases of incidence, prevalence, and mortality; DALYs) and standardized rates (age-standardized incidence rate [ASIR], prevalence rate [ASPR], mortality rate [ASMR], and disability rate [ASDR]). Furthermore, we analyzed temporal trends (1990-2021) for these indicators across three spatial scales: globally, across 21 GBD-defined regions, and in 204 countries/territories. This approach enabled systematic evaluation of both current disease patterns and their longitudinal evolution.

### 2.3 Trend analysis

To comprehensively assess the temporal trends of MS burden among WCBA (15–49 years) from 1990 to 2021, we employed a rigorous analytical approach combining two complementary methodologies. First, we conducted a linear trend analysis using estimated annual percentage change (EAPC) calculated through least squares linear regression models, implemented with the R package broom for efficient model fitting and result organization. This approach provided a robust measure of overall temporal patterns in age-standardized rates. Second, we performed segmented trend analysis using Joinpoint regression to identify potential inflection points in the temporal patterns of MS burden indicators. The Joinpoint analysis yielded three key metrics: APC for each identified temporal segment, AAPC across the entire study period, and their corresponding 95% confidence intervals. Statistical significance of trends was determined at α=0.05, with confidence intervals excluding zero indicating significant temporal changes. This dual-method analytical framework enabled both a comprehensive assessment of global temporal patterns and identification of specific periods with significant changes in MS burden, thereby providing valuable insights for developing targeted prevention strategies and evidence-based health policies. The methodology was carefully designed to ensure analytical rigor, result reproducibility, and comparability with other epidemiological studies.

Joinpoint regression analysis is a powerful segmented trend analysis method that automatically identifies inflection points in time-series data, partitioning long-term trends into distinct temporal segments. By calculating APC and AAPC, it precisely quantifies trend variations while demonstrating robustness against outliers through permutation testing. Its flexible algorithm supports multiple data distributions and customizable constraints, making it particularly valuable for evaluating MS intervention effectiveness, analyzing epidemiological transitions, and informing evidence-based policy decisions through high-quality visual outputs.

### 2.4 Pearson correlation

To examine the association between the SDI and MS burden metrics in WCBA (15–49 years), we employed complementary analytical approaches using global data from 204 countries and 21 regions (1990-2021). Our analysis incorporated: (1) Pearson correlation coefficients to quantify linear relationships between SDI and age-standardized rates (incidence [ASIR], prevalence [ASPR], mortality [ASMR], and disability [ASDR]); and (2) Gaussian Process Regression (GPR) modeling to capture potential non-linear associations while accounting for data uncertainty. This dual-method analytical framework enabled comprehensive characterization of how MS disease burden varies across the SDI spectrum, with Pearson correlations identifying general trends and GPR modeling providing nuanced understanding of complex patterns and uncertainty in the relationships.

### 2.5 Frontier analysis

This study employed frontier analysis methodology to quantitatively assess the relative efficiency of MS disease burden control across nations with varying socio-demographic development levels. The analytical framework established an empirical production possibility frontier representing the optimal achievable control of MS-related DALYs among WCBA (15–49 years) for each given SDI level.

Using data from the GBD study (2021), we constructed an efficiency frontier through deterministic non-parametric envelopment analysis, where countries lying on the frontier curve were classified as achieving optimal performance given their resource constraints. Technical efficiency scores were calculated as the radial distance from each country’s observed performance to the frontier benchmark, uses a color-coded system to classify countries by performance: blue for top-performing low-SDI countries near the optimal boundary, red for underperforming high-SDI countries farthest from it, and black for the 15 worst-performing nations globally. This visual tool quickly identifies exemplary models, systems needing improvement, and those requiring major reforms.

The analysis uses a color-coded system to classify countries by performance: blue for top-performing low-SDI countries near the optimal boundary, red for underperforming high-SDI countries farthest from it, and black for the 15 worst-performing nations globally. This visual tool quickly identifies exemplary models, systems needing improvement, and those requiring major reforms.

The model incorporated adjustments for healthcare system characteristics and population demographics to ensure valid cross-national comparisons. Sensitivity analyses were performed to verify the robustness of frontier placement against alternative model specifications. This approach enabled systematic identification of best-performing health systems and quantification of improvement potential across the development spectrum, while controlling for fundamental structural differences between nations.

### 2.6 Decomposition analysis

We conducted a comprehensive decomposition analysis to systematically examine the drivers of temporal changes in MS burden among WCBA (15–49 years) between 1990 and 2021. Using data from the GBD 2021 study, this analysis quantified the relative contributions of population aging, population growth, and epidemiological factors to observed trends in MS incidence, prevalence, mortality, and DALYs across global, regional, and SDI levels.

The analytical approach employed a regression-based decomposition framework that carefully distinguished between the effects of changing age structures, population size dynamics, and evolving age-specific disease rates. The model incorporated age-stratified population distributions to capture structural aging effects while simultaneously accounting for population growth patterns and temporal variations in epidemiological parameters. To ensure robust and comparable results, the analysis adjusted for potential interactions between demographic and epidemiological components through multivariable modeling techniques.

Regional stratification was performed at multiple levels, including five SDI-defined regions and 21 GBD regions, allowing for systematic examination of geographical variations in dominant drivers of MS burden. The decomposition accounted for data quality variations across locations and time periods through GBD’s standardized uncertainty estimation procedures, providing reliable attribution of burden trends to their underlying demographic and epidemiological determinants throughout the study period.

### 2.7 Age-period-cohort analysis

We conducted a robust APC analysis to systematically evaluate the temporal dynamics of MS burden among WCBA (15–49 years). The analytical framework employed a multi-dimensional approach to disentangle the complex interplay between age effects, temporal trends, and birth cohort influences on both DALYs and prevalence rates.

The analysis incorporated longitudinal age curve modeling to characterize the natural progression of MS burden across the reproductive lifespan, complemented by cross-sectional age curve analysis to capture period-specific variations. We computed rate ratios (RR) with 95% confidence intervals (derived through 1000 bootstrap replicates) to quantify differences between longitudinal and cross-sectional estimates, providing insights into temporal effect modifications.

For cohort-specific assessments, we implemented local drift estimation to calculate annual percentage changes in disease burden, while deviation analysis identified age groups exhibiting significant departures from expected epidemiological patterns. The Bayesian APC modeling framework was carefully applied to simultaneously estimate age, period, and cohort effects, incorporating constrained estimation techniques to address the inherent identifiability challenges of APC analysis. This approach enabled us to generate fitted cohort patterns that revealed generational trends in MS risk while accounting for potential confounding between temporal dimensions.

The analytical strategy included comprehensive sensitivity analyses to assess the robustness of findings to alternative model specifications and stratification approaches. All models incorporated adjustments for socio-demographic development levels and geographic region to examine potential effect modification by these structural factors, with uncertainty estimation procedures accounting for data quality variations across populations and time periods.

### 2.8 Health inequality analysis

This study systematically analyzed socio-economic inequalities in the disease burden of MS among WCBA (15–49 years) globally from 1990 to 2021, using standardized data from the GBD 2021 study. Countries were stratified by the SDI, while health inequalities were quantified using the Slope Index of Inequality (SII) and Concentration Index (CI), supplemented by visual analysis of concentration curves. ASDR served as the primary outcome measure. The analysis employed sophisticated statistical methods including 1000 posterior draws and joinpoint regression to account for data uncertainty, with comprehensive sensitivity analyses conducted to verify robustness. All procedures adhered to GBD ethical guidelines using approved aggregated data. This methodological framework provides a reliable approach for evaluating socio-economic gradients in MS disease burden.

### 2.9 Forecast analysis (2022–2040) based on 1990–2021 data

To forecast the global burden of MS in women of childbearing age (2022–2040), we applied two complementary modeling approaches: (1) Bayesian Age-Period-Cohort (BAPC) modeling, which integrated age-specific risks, temporal trends, and birth cohort effects while adjusting for SDI and region, and (2) Autoregressive Integrated Moving Average (ARIMA) modeling, optimized for time-series forecasting with external regressors (e.g., SDI, healthcare expenditure). Both models generated age-standardized projections for incidence (ASIR), prevalence (ASPR), mortality (ASMR), and disability-adjusted life years (ASDR), with ensemble weighting used to combine their strengths—BAPC for long-term trends and ARIMA for near-term fluctuations. Model performance was validated through back-testing (1990–2021), residual diagnostics, and comparative metrics (e.g., MAPE, Diebold-Mariano tests), ensuring robust 20-year burden estimates with 95% uncertainty intervals.

## 3 Results

### 3.1 Descriptive analysis of MS among WCBA

**Figure 1:**
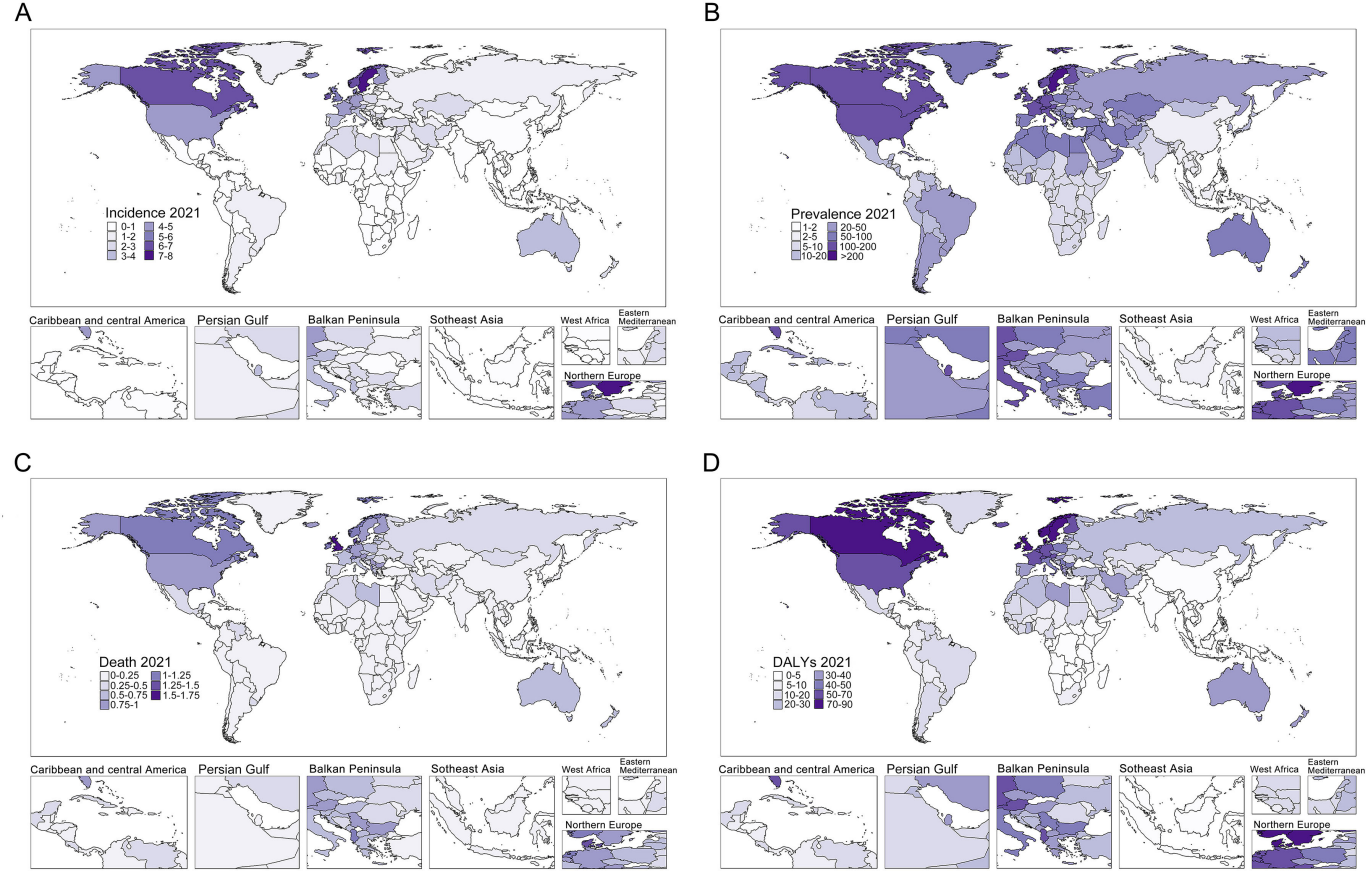
The global burden of MS among women of childbearing age in 204 countries and regions in 2021. **(A)** ASIR of MS; **(B)** ASPR of MS; **(C)** ASMR of MS; **(D)** ASDR of MS. Abbreviations: ASIR, age-standardized incidence rate; ASPR, age-standardized prevalence rate; ASMR, age-standardized mortality rate; ASDR, age-standardized disability rate; MS, Multiple Sclerosis.

**Figure 2:**
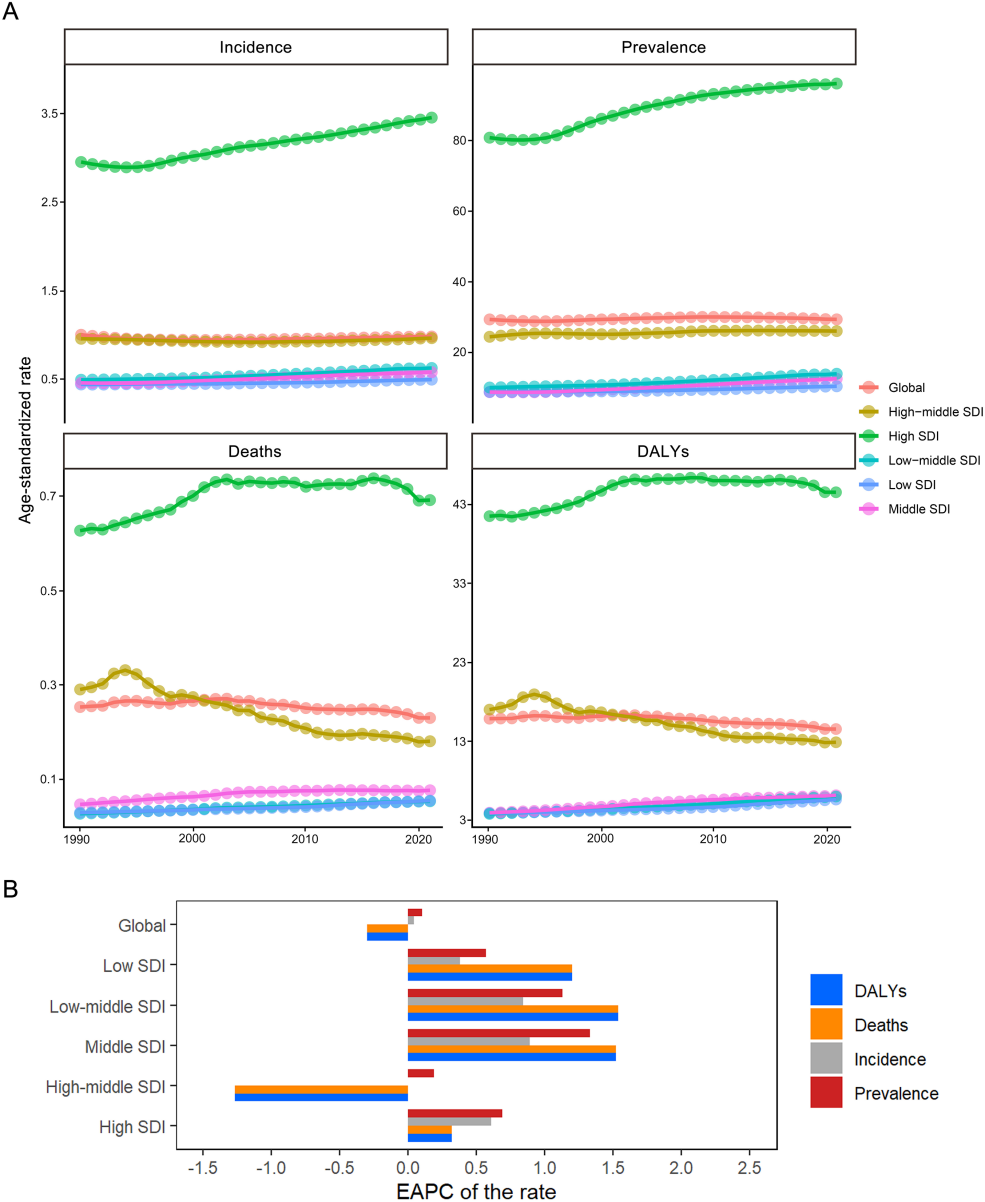
Trends and EAPC in disease burden of MS among WCBA from 1990 to 2021, Globally and 5 SDI Regions. Panel **(A)** shows trends in age-standardized incidence, prevalence, death, and DALYs rates of MS from 1990 to 2021, stratified by the global level and five SDI regions. Panel **(B)** presents EAPC of these four disease burden metrics across different SDI strata. MS, Multiple Sclerosis; EAPC, estimated annual percentage change; WCBA, women of childbearing age; DALYs, disability-adjusted life-years.

**Figure 3:**
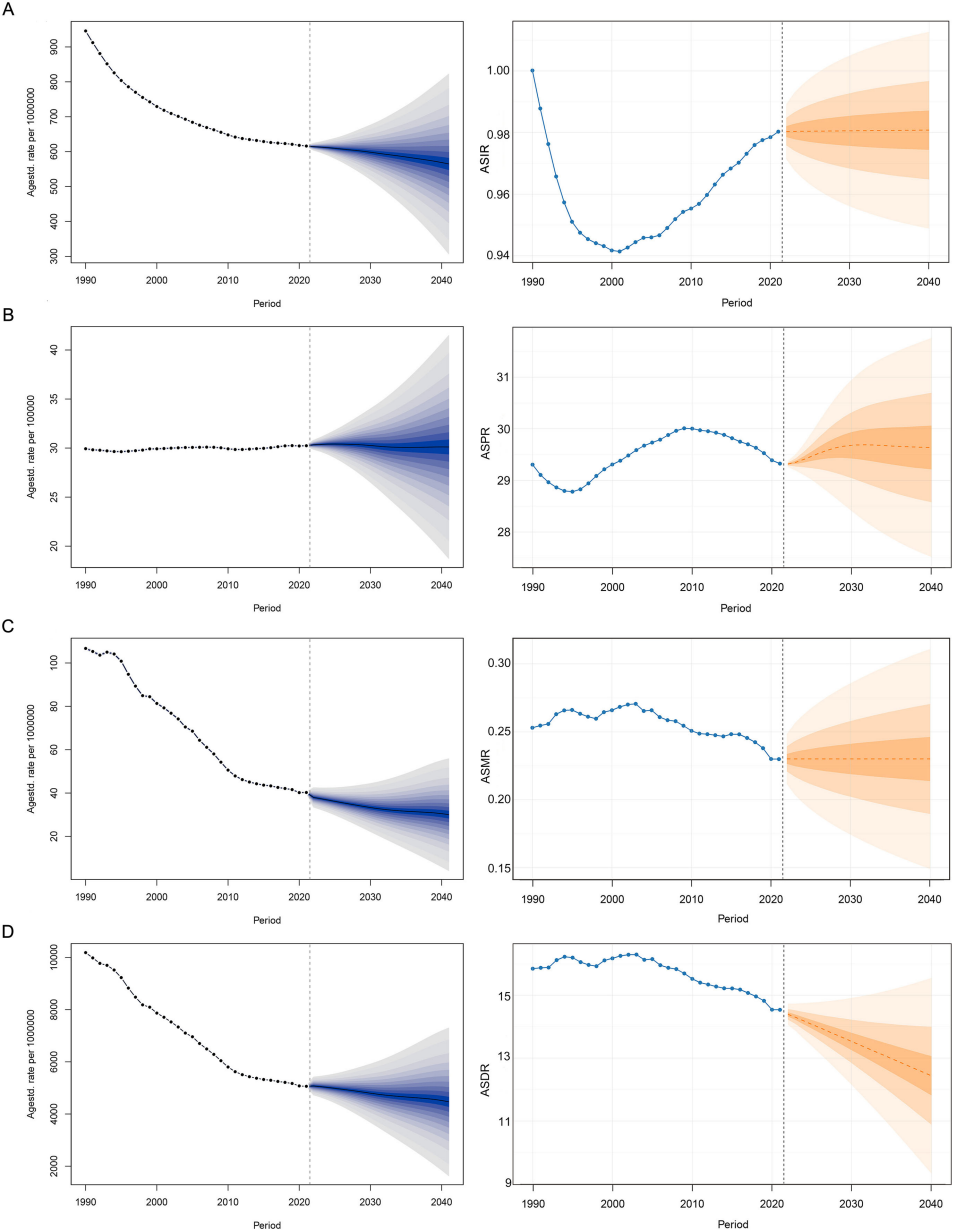
Projected global trends in ASIR **(A)**, ASPR **(B)**, ASMR **(C)**, ASDR **(D)** of MS among women of childbearing age over the next two decades (2022–2040) using BAPC and ARIMA models. ASIR, age-standardized incidence rate; ASPR, age-standardized rate; ASMR, age-standardized deaths rate; ASDR, age-standardized DALYs rate; DALYs, disability-adjusted life-years; MS, Multiple Sclerosis.

Global burden analysis of MS among WCBA (15–49 years) in 2021 revealed a crude incidence of 33,939.85 cases (95% UI: 29,760.56-38,684.01), crude prevalence of 606,711.23 cases (95% UI: 526,386.61-695,366.28), 2,111.99 deaths (95% UI: 1,934.47-2,320.57), and 269,173.6 DALYs (95% UI: 221,179.06-325,225.96). Age-standardized rates per 100,000 population were: incidence (ASIR) 0.98 (95% UI: 0.88-1.10), prevalence (ASPR) 29.32 (95% UI: 26.24-32.74), mortality (ASMR) 0.23 (95% UI: 0.21-0.24), and DALYs (ASDR) 14.54 (95% UI: 12.37-16.93) (**Figure 1**;**Supplementary Table 1**; **Supplementary Table 2**). Significant regional disparities were observed, with high-SDI regions exhibiting the highest burden. North America showed the highest age-standardized rates globally, while Oceania demonstrated the lowest burden. Socio-demographic index analysis indicated significantly higher crude and standardized rates in high-SDI countries compared to low-SDI regions (**Figure 2**). Age stratification identified peak prevalence, mortality and DALYs in the 45–49 age group, highest incidence in 30–34 year-olds, and lowest burden in 15–19 year-olds (**Supplementary Table 9**). Under the model of ARIMA, Age-standardized analysis predicts significant ASDR reduction, stable ASIR and ASMR to 2040, and ASPR reaching a plateau after 2030 (**Figure 3**). Consistent with the ARIMA model, the BAPC model also projected a sustained downward trend in ASDR over the next two decades. However, distinct patterns emerged for other indicators: both models showed modest declines in ASIR and ASMR, while ASPR remained relatively stable throughout the projection period.

### 3.2 Global trends in MS burden

**Figure 4:**
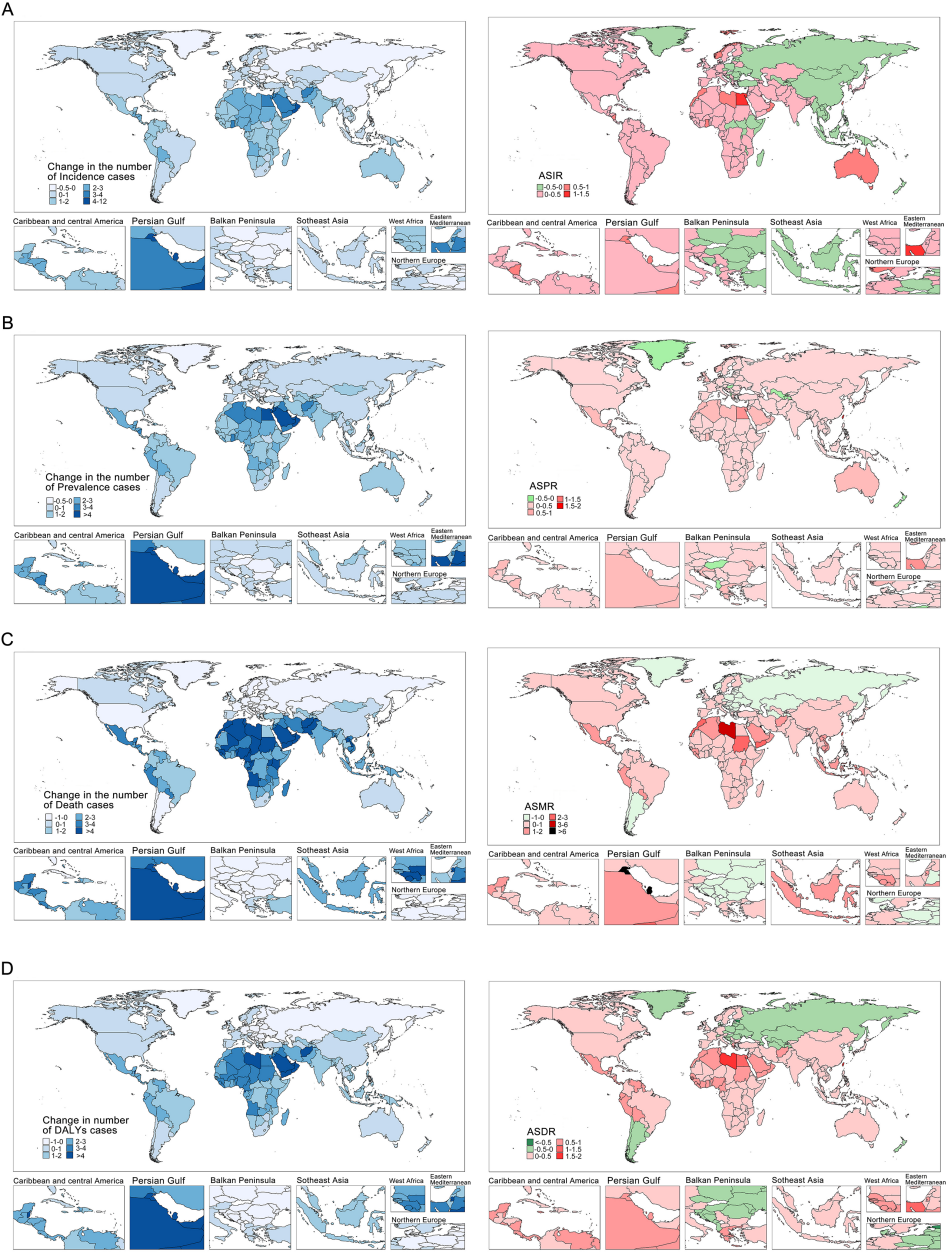
Trends in the number and age-standardized rates of incidence **(A)**, prevalence **(B)**, mortality **(C)**, and DALYs **(D)** of MS in women of childbearing age globally from 1990 to 2021. Abbreviation: MS, Multiple Sclerosis.

Longitudinal analysis of GBD data (1990-2021) demonstrated characteristic epidemiological transitions (**Figure 4**; **Supplementary Tables 4**-**8**). Crude cases of prevalence increased from 365,303 (95%UI: 307,711.42-431,148.96) to 606,711.23 (95%UI: 526,386.61-695,366.28), while ASPR showed minimal change from 29.31 (95%UI: 25.45-33.77) to 29.32 (95%UI: 26.24-32.74) (EAPC = 0.1, 95%UI: 0.06-0.14). Oceania uniquely demonstrated decreasing ASPR (EAPC=-0.04, 95%UI: -0.1-0.01). Similarly, crude cases of incidence rose from 22,866.69 (95%UI: 19,661.09-26,640.68) to 33,939.85 (95%UI: 29,760.56-38,684.01), while ASIR slightly declined from 1.0 (95%UI: 0.88-1.14) to 0.98 (95%UI: 0.88-1.1) (EAPC = 0.04, 95%UI: -0.02-0.1). Seven regions showed significant ASIR declines (**Supplementary Table 4**). Mortality indicators demonstrated consistent improvement, with crude cases of death increasing from 1,797.77 (95%UI: 1,698.82-1,896.81) to 2,111.99 (95%UI: 1,934.47-2,320.57) but ASMR declining from 0.25 (95%UI: 0.24-0.26) to 0.23 (95%UI: 0.21-0.24) (EAPC=-0.35, 95%UI: -0.46--0.24). ASDR decreased from 15.85 (95%UI: 13.65-18.37) to 14.54 (95%UI: 12.37-16.93) (EAPC=-0.3, 95%UI: -0.37--0.23), indicating improved global MS management.

### 3.3 Generalized estimating equations analysis

**Figure 5:**
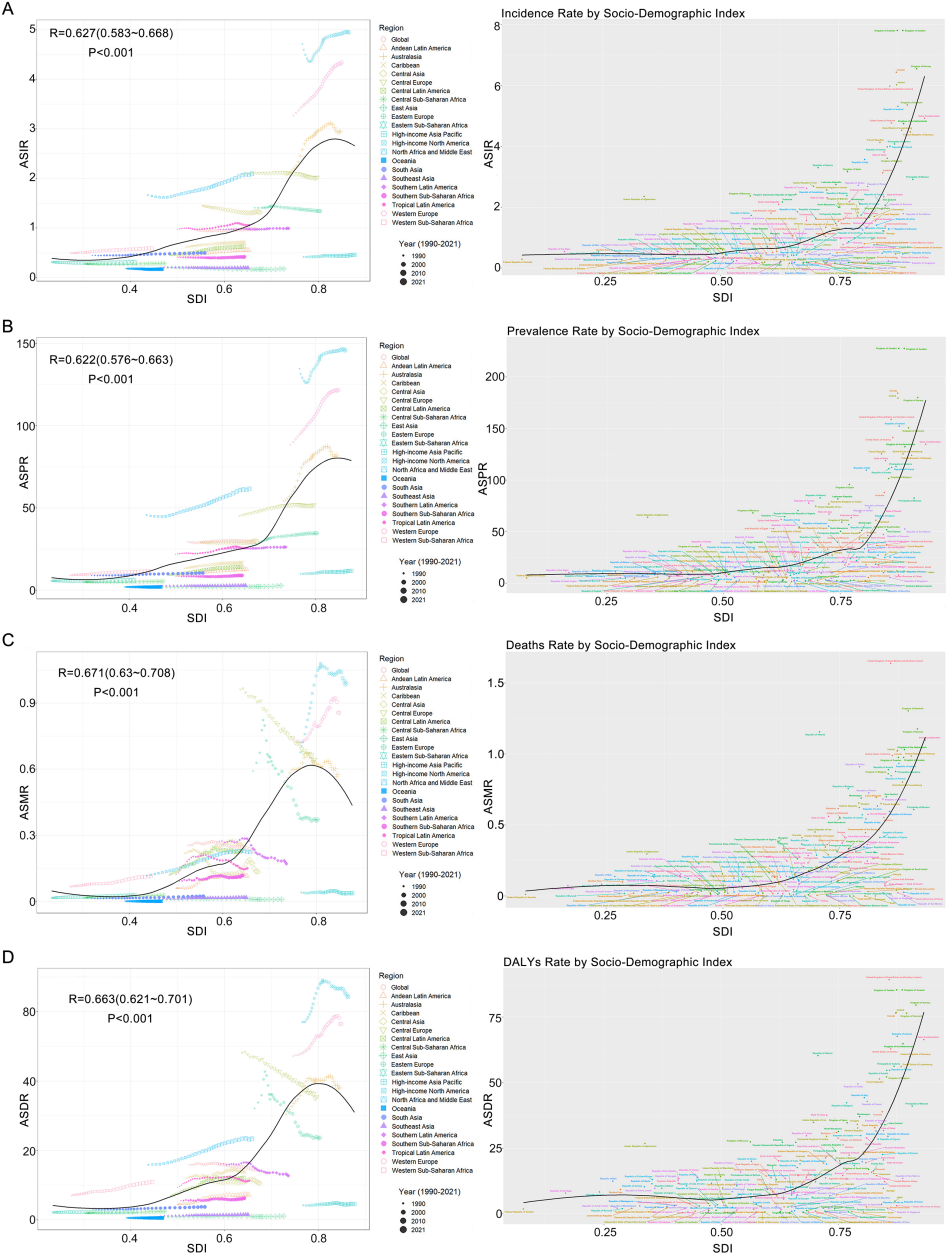
Correlation analysis of the ASIR **(A)**, ASPR **(B)**, ASMR **(C)**, and ASDR **(D)** of MS in women of childbearing age with the SDI across 21 regions and 204 countries. Abbreviations: ASIR, age-standardized incidence rate; ASPR, age-standardized prevalence rate; ASMR, age-standardized mortality rate; ASDR, age-standardized dis-ability rate; SDI, socio-demographic index; MS, Multiple Sclerosis.

From 1990-2021, cases of incidence increased 48%, cases of prevalence 66%, and deaths 17%, while DALYs decreased 7% globally. Age-standardized metrics showed ASIR and ASPR increases of 0.04 (95%CI: -0.02-0.1) and 0.1 (95%CI: 0.06-0.14) respectively, contrasted with ASMR and ASDR declines of -0.35 (95%CI: -0.46--0.24) and -0.3 (95%CI: -0.37--0.23) (**Supplementary Tables 5**-**8**). Regional variations were pronounced (**Figure 4**; **Supplementary Table 4**): Australasia showed greatest incidence increase versus East Asia’s largest decrease; Central Latin America had maximal prevalence growth; Andean Latin America showed greatest mortality increase while Eastern Europe demonstrated most improvement. SDI-based EAPC analysis revealed ASIR, ASPR, ASMR and ASDR increases in low through high-SDI regions, except high-middle SDI where ASDR and ASMR declined (**Figure 2**). Nonlinear Pearson correlations showed minimal burden changes below SDI 0.4, but significant increases above this threshold (**Figures 5**, **2**). Age-stratified analysis found rising ASIR across all ages, increasing ASPR (most notably 15–19 years), elevated ASMR, and greatest ASDR increases in 20–49 year-olds (particularly 45–49 years) (**Supplementary Table 9**).

### 3.4 Joinpoint regression analysis

Joinpoint analysis of global trends (1990-2021) revealed: ASMR and ASDR showed significant declines (most rapid 2017-2021) with 4–5 breakpoints; ASIR and ASPR generally increased, with fastest growth during 2006-2021 (4 breakpoints) and 1995-2003 (6 breakpoints) respectively (**Table 1**). ASIR transitioned from decline (1990-2000) to increase (post-2000). ASPR demonstrated complex fluctuations: slight decline (1990-1995), increase (1995-2009), then decline. ASMR shifted from slight increase (1990-2003) to significant decline. ASDR showed the most variability: slight increases (1990-1994; 1997-2003) alternating with declines (1994-1997; 2003-2021), most notably 2017-2021.

**Table 1:** Joinpoint regression models for disease burden indicators of MS in WCBA globally.

Measure	Segment	Lower endpoint	Upper endpoint	APC	Lower CI	Upper CI	Test statistic	P
DALYs	0	1990	1994	0.7	0.3	1.0	4.2	0.001
	1	1994	1997	-0.5	-1.6	0.5	-1.1	0.310
	2	1997	2003	0.4	0.1	0.6	3.3	0.005
	3	2003	2012	-0.7	-0.8	-0.6	-12.3	<0.001
	4	2012	2017	-0.3	-0.7	0.1	-1.8	0.098
	5	2017	2021	-1.0	-1.4	-0.7	-6.0	<0.001
Incidence	0	1990	1992	-1.2	-1.5	-0.9	-8.7	<0.001
	1	1992	1995	-0.9	-1.2	-0.6	-6.5	<0.001
	2	1995	2000	-0.2	-0.3	-0.1	-4.4	<0.001
	3	2000	2006	0.1	0.0	0.2	3.9	0.001
	4	2006	2021	0.2	0.2	0.3	47.6	<0.001
Mortality	0	1990	1993	1.3	0.0	2.5	2.2	0.045
	1	1993	2003	0.3	0.1	0.5	2.7	0.015
	2	2003	2011	-1.0	-1.3	-0.6	-5.8	<0.001
	3	2011	2017	-0.2	-0.8	0.5	-0.5	0.596
	4	2017	2021	-1.9	-3.0	-0.8	-3.5	0.003
Prevalence	0	1990	1992	-0.6	-0.9	-0.3	-4.6	0.001
	1	1992	1995	-0.2	-0.5	0.1	-1.7	0.118
	2	1995	2003	0.4	0.3	0.4	25.6	<0.001
	3	2003	2009	0.2	0.2	0.3	11.3	<0.001
	4	2009	2013	-0.1	-0.2	0.0	-1.5	0.171
	5	2013	2018	-0.2	-0.3	-0.1	-6.5	<0.001
	6	2018	2021	-0.4	-0.5	-0.3	-7.0	<0.001

APC, Annual Percent Change; MS, Multiple Sclerosis; DALYs, Disability-Adjusted Life Years.

### 3.5 Frontier analysis

**Figure 6:**
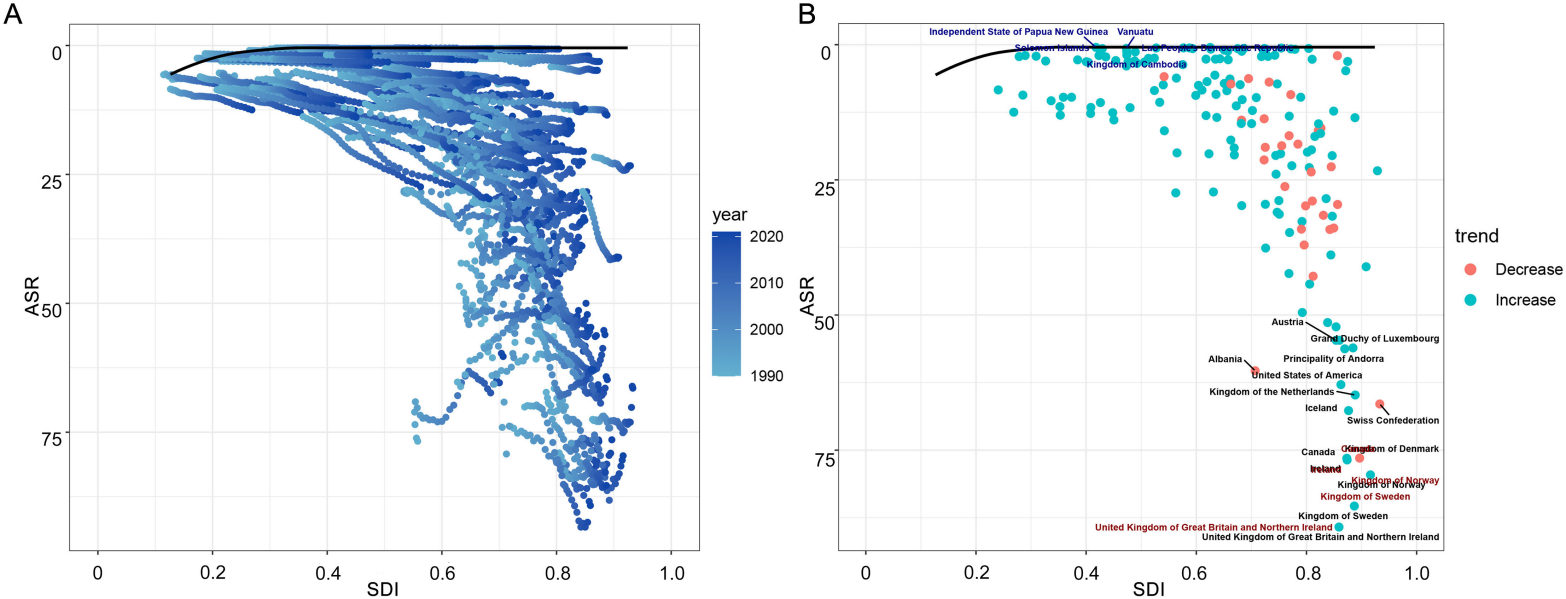
Frontier analysis of MS in women of childbearing age and SDI at the regional and national levels based on ASDR in the GBD. **(A)** Relationship between ASR and SDI from 1990 to 2021; **(B)** Trends in ASR by country. ASDR, age-standardized DALY rate; ASR, age-standardized rate; MS, Multiple Sclerosis; SDI, socio-demographic index.

Frontier analysis evaluated national efficiency in controlling MS-related DALYs across SDI levels (**Figure 6**). Low-SDI countries (Papua New Guinea, Vanuatu, Solomon Islands, Laos, Cambodia) demonstrated optimal efficiency (nearest frontier line, blue), suggesting effective burden control despite limited resources. High-SDI nations (United Kingdom, Sweden, Norway, Ireland, Canada) showed greatest improvement potential (furthest from frontier, red), particularly the UK and Sweden. Fifteen least efficient countries (black) highlighted global disparities. Results indicate MS burden control variations stem from differences in healthcare strategies, resource allocation and prevention coverage, revealing improvement opportunities for high-SDI countries and efficient models in resource-limited settings.

### 3.6 Decomposition analysis

**Figure 7:**
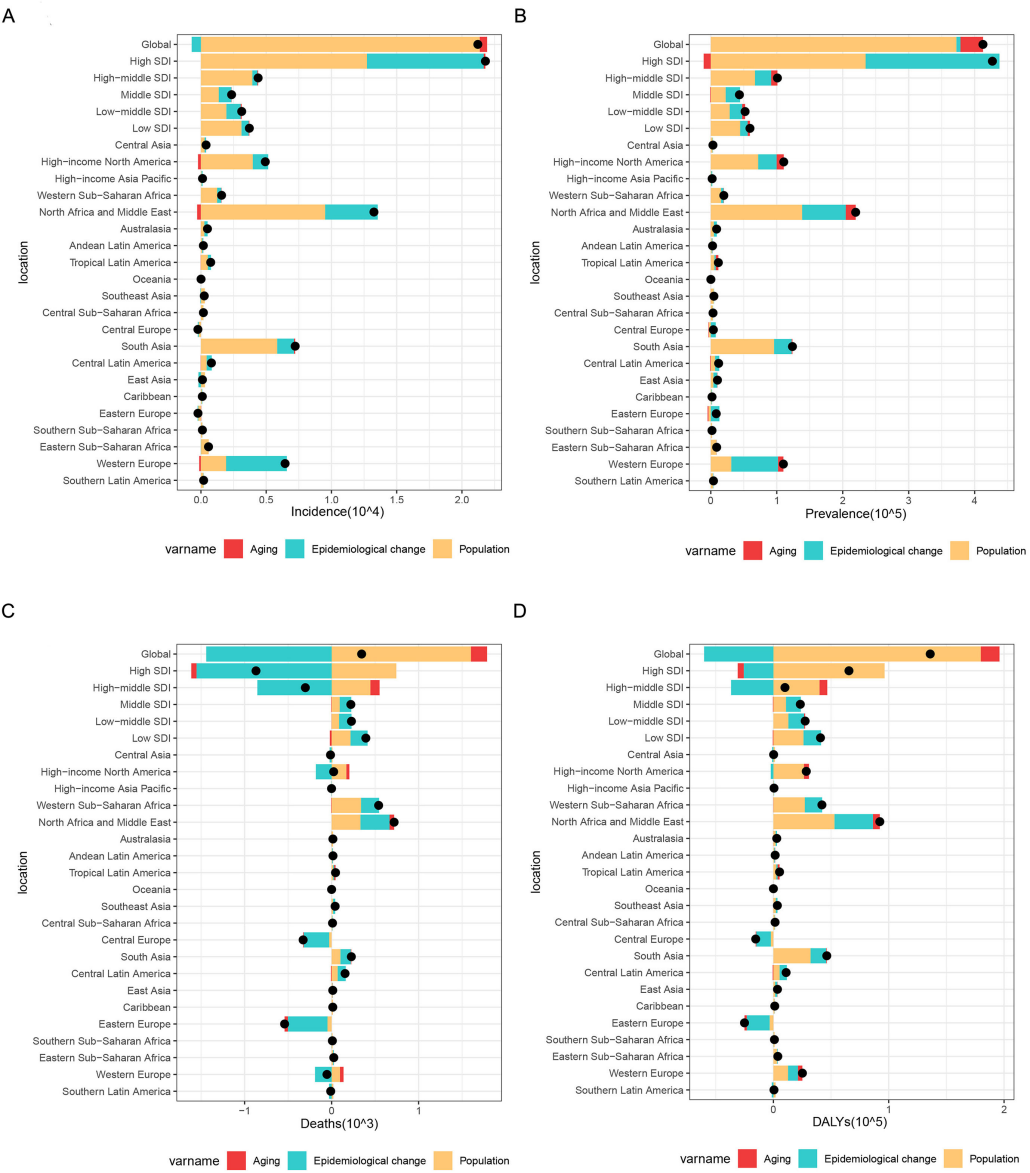
Decomposition analysis of the changes in incidence **(A)**, prevalence **(B)**, mortality **(C)**, and DALYs **(D)** for MS in women of childbearing age from 1990 to 2021, by SDI and GBD regions. Abbreviations: MS, Multiple Sclerosis; SDI, socio-demographic index.

Our decomposition analysis quantified contributions of population aging, growth and epidemiological factors to MS burden across 5 SDI and 21 GBD regions (1990-2021). Rising incidence, prevalence, mortality and DALYs were primarily driven by population growth (**Figure 7**). Western Europe’s increasing incidence/prevalence reflected epidemiological changes, while mortality declines in high-income and Central/Eastern European regions resulted from epidemiological improvements. Central/Eastern Europe’s DALYs reduction followed similar patterns, whereas other regions showed population aging driving DALYs increases. These findings demonstrate heterogeneous burden determinants across regions, reflecting variations in healthcare resources and disease management.

### 3.7 Age-period-cohort analysis

Age-cohort analysis revealed MS DALYs increasing steadily from age 15-19, plateauing at 20-30, then rising sharply (**Supplementary Figure 1**). Longitudinal versus cross-sectional RR declined from 1.0 to 0.6, suggesting reduced age-specific risk in recent birth cohorts. Local drift analysis showed decreasing annual percentage changes with age (coefficient=-1.433), indicating global DALYs improvement, though 20–25 year-olds showed significant positive deviations (>0.5), warranting targeted intervention. Birth cohort analysis demonstrated: RR increase from 1.086 (95%CI: 0.779-1.515) in 1990–1991 to 1.115 (95%CI: 0.835-1.49) in 1992-1996, then decline to 0.719 (95%CI: 0.54-0.957) by 2017-2021. Fitted cohort patterns showed nonlinear trends, with pre-2008 increases transitioning to declines. Early cohorts (1947-1951) had highest RR (2.721, 95%CI: 1.755-4.218), declining to 0.913 (95%CI: 0.589-1.417) by 1992-1996, then rebounding to 1.413 (95%CI: 0.715-2.794) in 2002-2006, suggesting generational risk fluctuations.

Prevalence patterns mirrored DALYs but with more pronounced longitudinal-cross-sectional RR differences (1.1→0.7). Local drift showed 1.191% annual prevalence reduction, though 30–35 year-olds demonstrated positive deviations (>0.3), indicating unique risk factors. Cohort analysis revealed consistent improvements: RR declined from 1.146 (95%CI: 0.909-1.444) in 1990–1991 to 0.805 (95%CI: 0.662-0.979) by 2017-2021. Early cohorts (1947-1951) showed RR = 2.081 (95%CI: 1.51-2.869), decreasing to 1.033 (95%CI: 0.854-1.251) by 1972-1976, then stabilizing (2002–2006 RR = 0.965, 95%CI: 0.531-1.754). Fitted models confirmed post-2008 prevalence declines.

### 3.8 Health inequality of MS among WCBA

**Figure 8:**
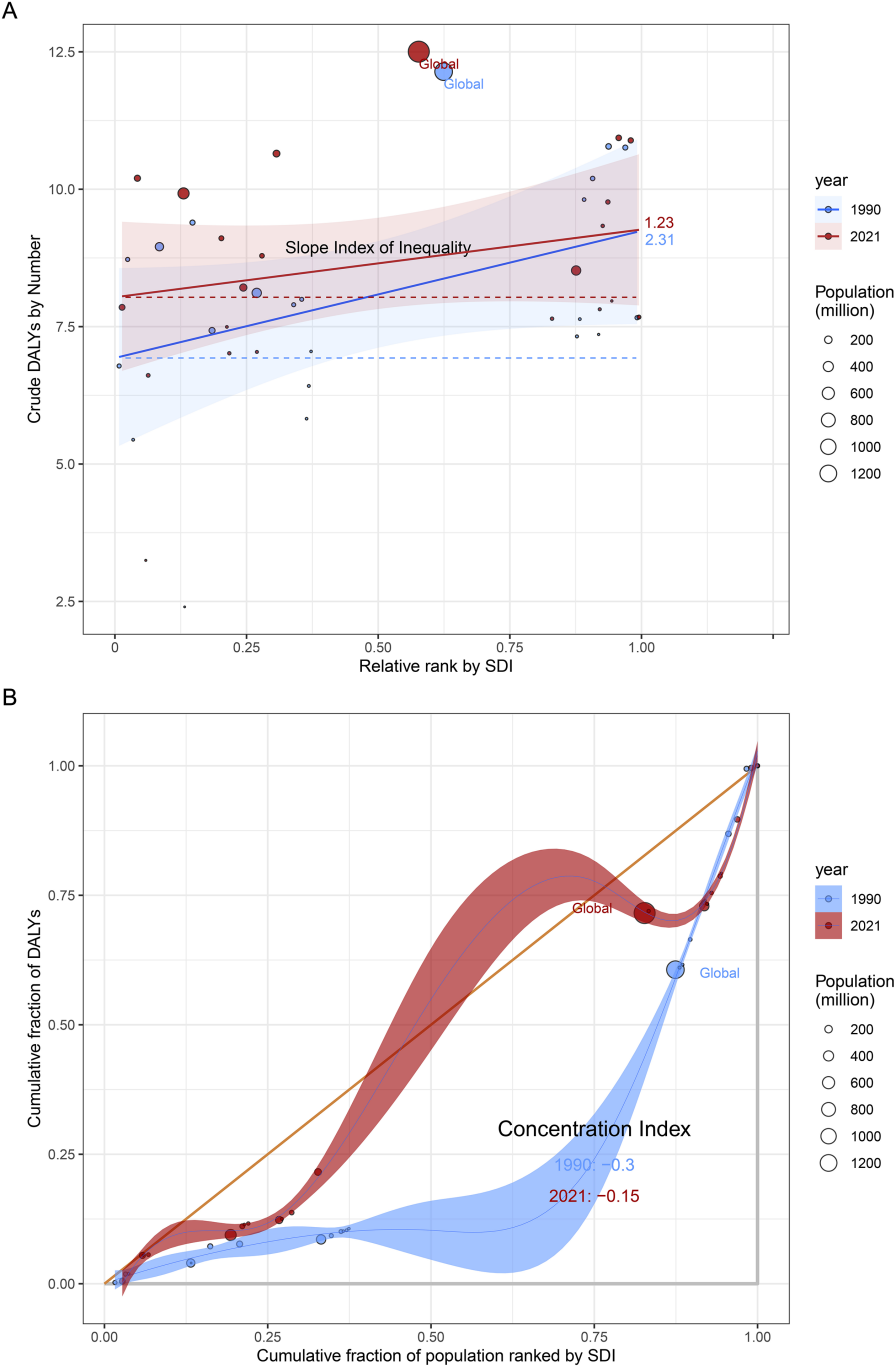
SDI-related health inequality regression **(A)** and concentration **(B)** curves for the Deaths of MS among women of childbearing age worldwide, 1990 and 2021. Abbreviations: SDI, socio demographic index; MS, Multiple Sclerosis.

This study analyzed socio-economic inequalities in the disease burden of MS among WCBA globally. The SII analysis revealed that in 2019 (**Figure 8**), the disparity in DALY rates between the lowest and highest SDI quintiles was 2.31 per 100,000 (95%UI: 1.23-3.42). The SII for a specific high-risk subgroup (labeled “Crucially”) was 1.23 (95%UI: 0.75-1.68), indicating a disproportionately higher disease burden among populations with lower socio-economic status.

The CI analysis further corroborated these findings. The CI improved significantly from -0.30 (95%CI: -0.35 to -0.25) in 1990 to -0.15 (95%CI: -0.18 to -0.12) in 2021 (*p* < 0.01), demonstrating a persistent but decreasing pro-poor inequality in disease distribution. The concentration curve showed that in 2021, the bottom 40% of the population bore approximately 60% of the DALY burden, representing an improvement compared to 1990 when the bottom 30% carried 65% of the burden.

Demographic data indicated that the global population of WCBA increased from 201 million (95%CI: 198–205 million) in 1990 to 1.23 billion (95%CI: 1.21-1.25 billion) in 2021, a 512% increase. Notably, despite this dramatic population growth, the CI showed consistent improvement, suggesting potential positive effects of healthcare interventions. These findings provide critical evidence for targeted health policies, emphasizing the need for enhanced early screening and disease management for women in low-SDI regions, along with improved diagnostic systems to better assess the true disease burden. Future research should employ multilevel modeling to quantify the independent contributions of various social determinants (e.g., education, income, healthcare accessibility) to observed inequality trends.

## 4 Discussion

Our study leverages data from the GBD database (1990-2021), the most authoritative global public health data platform. The GBD’s core strengths lie in its standardized Bayesian statistical models that integrate multi-source data from 200+ countries/regions, 369 diseases, and 87 risk factors, overcoming traditional statistical geographical limitations. It provides high-precision temporal data (including provincial/state-level granularity and age-sex stratification) since 1990 and pioneered composite metrics like DALYs. All data include 95% uncertainty intervals ensuring reliability. Its open-access nature and visualization tools (e.g., GBD Compare) enable direct application of complex data to research and policymaking, establishing it as the gold standard for global health policy formulation by WHO and other agencies (21–23). Through multiple statistical models and visualization methods, we comprehensively analyzed and presented the MS burden (including incidence, prevalence, mortality, DALYs, and ASRs) among WCBA at global, regional, and national levels, with comprehensive future projections. These efforts have made the global MS burden among reproductive-aged women clear and actionable. We specifically examined relationships between MS burden and SDI, time series, and age groups from 1990 to 2021. This represents the first comprehensive analytical framework simultaneously evaluating incidence, prevalence, mortality, DALYs, and age-standardized rates (ASRs) while incorporating socio-demographic index (SDI) and age stratification to reveal MS burden heterogeneity. Our work fills critical gaps in global MS burden research and projections for reproductive-aged women, enhancing understanding and informing health policy development.

The 2021 data reveal significant geographical disparities in MS burden among reproductive-aged women. High-income North America shows the highest age-standardized prevalence rate (ASPR 32.74, 95% UI: 26.24-32.74), attributable to synergistic effects of environmental risk factors (e.g., 42% vitamin D deficiency rate) (24) and genetic susceptibility (15-25% HLA-DRB1*15:01 allele frequency) (25). Age-specific analysis identifies peak incidence at 30–34 years (ASIR 0.98) (26), while rising mortality at 45–49 years (ASMR 0.23) correlates closely with menopausal hormonal changes. Projection models indicate declining age-standardized disability rates (ASDR), reflecting positive effects of disease-modifying therapies. We recommend: (1) implementing vitamin D fortification programs in high-SDI regions; (2) developing targeted therapies for perimenopausal patients; and (3) establishing a global MS surveillance network to address regional disparities and demographic challenges.

GBD data demonstrate that from 1990-2021, MS prevalence and incidence among reproductive-aged women showed overall increases, though age-standardized rates (ASPR and ASIR) changed minimally (EAPCs 0.1 and 0.04, respectively), suggesting case growth primarily stems from population expansion and diagnostic criteria evolution (27). Notably, Oceania showed declining ASPR (EAPC=-0.04), while East Asia and Eastern Europe exhibited significant ASIR reductions (e.g., East Asia EAPC=-0.69), potentially reflecting underdiagnosis or environmental improvements (e.g., vitamin D levels). Conversely, substantial declines in age-standardized mortality and DALY rates (ASMR and ASDR EAPCs -0.35 and -0.30, respectively) demonstrate the impact of widespread disease-modifying therapy (DMT) adoption and care quality improvements (28). However, elevated perinatal mental health risks in MS patients underscore the need for enhanced psychological screening. Future research should investigate regional variation drivers (e.g., genetic susceptibility, Epstein-Barr virus infection, immune regulation) (25) and optimize global MS surveillance frameworks for improved data comparability (29).

GBD-based analysis reveals that during 1990-2021, age-standardized MS prevalence among reproductive-aged women remained stable (EAPC = 0.1) despite 66% absolute case growth (30), likely reflecting diagnostic advances (31) and DMT dissemination (32). Crucially, we identified a nonlinear relationship between socio-demographic index (SDI) and MS burden, with high-SDI regions showing significantly elevated risk (30). Age stratification revealed peak burden at 45–49 years, potentially associated with menopausal hormonal changes (33). Based on epidemiological evidence, we recommend: (1) strengthening environmental risk factor control (e.g., vitamin D deficiency, smoking) in high-SDI regions (24); (2) optimizing therapeutic strategies for perimenopausal MS patients; and (3) establishing a global MS surveillance network to monitor burden evolution (2). These measures will help address regional disparities and population aging challenges in MS management.

Joinpoint regression analysis uncovers complex temporal patterns in global MS burden among reproductive-aged women (1990-2021). The most pronounced declines in ASMR and ASDR (2017-2021) correlate strongly with widespread DMT adoption and diagnostic improvements following the 2017 McDonald criteria revision (28). The pre-2003 ASMR increase may reflect limitations of early therapies, while accelerated ASDR decline (2017-2021) likely stems from high-efficacy DMTs (e.g., ocrelizumab). Conversely, rising ASIR and ASPR (peaking during 2006–2021 and 1995-2003, respectively) may involve cumulative environmental exposures (e.g., vitamin D deficiency, EBV infection) and enhanced diagnostic sensitivity (34).Regional variations merit attention—East Asia’s ASIR decline may reflect diagnostic lag, while Western Europe’s high burden indicates robust surveillance (2). Future research priorities include: 1) developing personalized therapies for reproductive-aged women; 2) optimizing perinatal management to reduce postpartum relapse; and 3) establishing unified global MS surveillance frameworks for improved data comparability.

Frontier analysis indicates that low-SDI countries (e.g., Papua New Guinea, Cambodia) achieve relatively efficient MS control despite limited resources, potentially due to lower intrinsic incidence (35) and early intervention strategies (36). Conversely, suboptimal performance in high-SDI nations (e.g., UK, Sweden) may reflect overdiagnosis (37), treatment delays (38), or resource allocation inefficiencies. Notably, MS burden in reproductive-aged women shows strong associations with hormonal fluctuations (39) and inadequate pregnancy management (40), suggesting intervention optimization opportunities. Future studies should focus on: improving healthcare efficiency pathways in high-SDI settings, adapting low-SDI best practices (e.g., primary care integration), and strengthening multinational collaboration to reduce management disparities (28).

Decomposition analysis demonstrates that global MS burden increases (1990-2021) primarily stem from population growth (35), with marked regional heterogeneity: Western Europe’s incidence rise mainly reflects epidemiological factors (e.g., diagnostic criteria changes and environmental triggers) (28), while Central/Eastern Europe’s mortality decline benefits from care optimization (38). Importantly, DALY increases in most regions strongly correlate with population aging (41), particularly exposing healthcare system vulnerabilities in managing age-related MS complications in resource-limited settings. Future efforts require targeted early interventions in high-burden regions (40) and aging-adaptive healthcare infrastructure development, complemented by transnational research on environmental risk factors (42) to alleviate epidemiological loads.

Our study elucidates distinct age-period-cohort characteristics of MS burden among reproductive-aged women: both DALYs and prevalence demonstrate early-life increases (from 15–19 years) followed by plateauing, yet recent birth cohorts (2017-2021) show significantly reduced age-specific risk (RR = 0.719), aligning with global trends of delayed MS onset (43). Local drift analysis reveals 1.433% annual DALY reduction, likely reflecting diagnostic criteria revisions (28) and therapeutic advances, though significant positive deviations in 20-25-year-olds indicate unaddressed risk factors (e.g., adolescent vitamin D deficiency (44) or EBV infection windows). Cohort effect analysis shows RR peaking at 2.721 for 1947–1951 births, with gradual decline post-1990 potentially linked to improved prenatal nutrition (e.g., folic acid fortification) and reduced childhood environmental exposures. However, the temporary rebound in 2002–2006 cohorts suggests generational risk fluctuations possibly tied to periodic environmental toxin exposures (e.g., pesticides) (35). Key research priorities include: 1) targeted biomarker screening for 20-30-year-olds; 2) longitudinal cohort monitoring of prenatal/childhood environmental risks; and 3) healthcare resource optimization in high-SDI countries to address delayed onset patterns (2).

In terms of both clinical implications and public health, these findings point to a number of priorities for women of childbearing age. In light of the positive deviation in burden observed among women in the age group of 20–25 and peak incidence at 30–34, increased clinical suspicion should be directed towards the age bracket of 15–30 years. Allowing for low-threshold application of the 2017 McDonald Criteria would aid in diagnosis (28). An additional aspect of perinatal management is that it must be performed in accordance with ECTRIMS/EAN guidelines. Family planning advice must be given before conception. Individual assessment of the risks and benefits of disease-modifying therapy during pregnancy and postpartum (when relapse risk is greatest) is required (45, 46). The unaccounted for increased risk of 2002–2006 birth cohort and persistent health inequality (CI= -0.15) call attention to the need for implementation of vitamin D supplementation as well as smoking cessation targeted preventive interventions as suggested by the WHO and National MS Society recommendations for at-risk populations. Together, these measures provide a means of transforming the observed epidemiological trends into concrete improvements in screening, treatment, and prevention for this population.

This study has several limitations that warrant consideration. First, the retrospective nature of GBD data may lead to potential underestimation of MS burden in low-SDI countries due to incomplete medical records and underreporting. Second, many findings rely on modeled estimates that could be constrained by data gaps and inherent modeling assumptions. Additionally, both BAPC and ARIMA models are limited to capturing age, period, and cohort effects, and do not account for other potential confounders such as economic development, advances in MS treatment, or major societal disruptions like war. As a result, the projections, while scientifically informed, carry considerable uncertainty. Finally, as a cross-sectional analysis based on the GBD database, variations in reporting standards, diagnostic criteria, and methodologies across different national statistical and health agencies may affect data validity and accuracy. Such inconsistencies could result in underestimation of true MS incidence rates, and the inherent time lag in GBD data may not fully capture recent epidemiological patterns. Future research should prioritize enhanced data collection and quality improvement initiatives, particularly in resource-limited settings where substantial proportions of undiagnosed MS cases may remain unrecorded, potentially skewing the representativeness of actual disease burden estimates.

## 5 Conclusion

This global analysis of MS in women of childbearing age (1990-2021) reveals significant epidemiological trends: while crude incidence and prevalence increased by 48% and 66% respectively, age-standardized mortality and disability rates declined substantially (-0.35% and -0.30% annually), demonstrating improved disease management. The study identifies critical age-specific patterns (peak incidence at 30–34 years, maximum burden at 45–49 years) and regional disparities, with high-SDI countries showing paradoxically high burden yet improvement potential, while low-SDI regions demonstrated unexpected control efficiency. These findings underscore the need for targeted interventions addressing age-specific risks and optimized healthcare strategies to reduce global disparities in MS management for this vulnerable population.

## Data Availability

Publicly available datasets were analyzed in this study. This data can be found here: https://ghdx.healthdata.org/gbd-results-tool.

## Supplementary material

Supplementary Table 1 The number of incident cases, prevalent cases, deaths, and DALYs for women of childbearing age with MS in 2021 globally, across 21 regions, and in 204 countries.

Supplementary Table 2 ASIR, ASPR, ASMR, and ASDR of MS in Women of Childbearing Age in 2021, Globally and by Country.

Supplementary Table 3 Disease burden of MS in women of childbearing age by age group in 2021.

Supplementary Table 4 The Estimated Annual Percentage Change (EAPC) in ASIR, ASPR, ASMR, and ASDR for MS in Women of Childbearing Age from 1990 to 2021, Globally, by Country, and by Region.

Supplementary Table 5 Global and regional prevalence of MS in 1990 and 2021, and EAPC of ASR from 1990 to 2021.

Supplementary Table 6 Global and regional Incidence of MS in 1990 and 2021, and EAPC of ASR from 1990 to 2021.

Supplementary Table 7 Global and regional Deaths of MS in 1990 and 2021, and EAPC of ASR from 1990 to 2021.

Supplementary Table 8 Global and regional DALYs of MS in 1990 and 2021, and EAPC of ASR from 1990 to 2021.

Supplementary Table 9 The EAPC in Disease Burden of MS in Women of Childbearing Age by Age Group from 1990 to 2021.

Supplementary Figure 1 Age, period, and cohort trends in global MS among women of childbearing age. **(A)** prevalence **(B)** DALYs.
