# Supplementary material for: The epidemiological trends of multiple sclerosis among women of child-bearing age: a global analysis from 1990 to 2021 and forecasts to 2040
Source: Front Immunol. 2026 May 1;17:1677178. doi: 10.3389/fimmu.2026.1677178 (PMC13175834; doi:10.3389/fimmu.2026.1677178)
Supplement: Supplementary Table 1 — The number of incident cases, prevalent cases, deaths, and DALYs for women of childbearing age with MS in 2021 globally, across 21 regions, and in 204 countries. [file Table1.docx]

Supplementary Table 1: The number of incident cases, prevalent cases, deaths, and DALYs for women of childbearing age with MS in 2021 globally, across 21 regions, and in 204 countries

| location | Prevalence (95%UI) | Incidence (95%UI) | Deaths (95%UI) | DALYs (95%UI) |
| --- | --- | --- | --- | --- |
| Global | 606711.23(526386.61-695366.28) | 33939.85(29760.56-38684.01) | 2111.99(1934.47-2320.57) | 269173.6(221179.06-325225.96) |
| Low SDI | 29346.09(23288.34-36488.49) | 2137.6(1744.75-2590.61) | 218.53(110.04-330.57) | 22132.76(14330.76-30372.96) |
| Low-middle SDI | 77240.51(61824.51-95687.93) | 5194.31(4253.23-6300.76) | 271.62(207.26-356.58) | 37299.28(28831.56-46892.01) |
| Middle SDI | 94725.24(76567.22-113824.76) | 5980.16(4951.83-7158.83) | 427.72(376.92-486.87) | 47763.16(39069.92-58353.17) |
| High-middle SDI | 103239.85(91837.25-116011.26) | 5016.54(4480.19-5632.99) | 462.07(405.19-522.26) | 49540.6(40813.8-58389.88) |
| High SDI | 301544.38(272233.7-333366.32) | 15579.44(14176.55-17093.99) | 728.73(694.88-763.06) | 112113.84(90051.39-135682.95) |
| 21 Regions |  |  |  |  |
| Andean Latin America | 2109.37(1648.4-2682.39) | 154.79(122.2-190.03) | 11.07(7.36-16.49) | 1141.37(859.65-1502.09) |
| Australasia | 7325.46(6138.24-8719.92) | 412.81(357.26-474.94) | 21.28(18.66-24.18) | 2885.87(2208.76-3650.42) |
| Caribbean | 2207.53(1770.02-2663.71) | 145.84(119.74-172.54) | 23.47(18.58-30.28) | 1799.07(1470.64-2235.24) |
| Central Asia | 5313.93(4314.41-6640.64) | 491.19(422.14-579.07) | 12.69(9.55-16.22) | 2089.35(1502.34-2712.46) |
| Central Europe | 19980.89(17752.89-22207.69) | 905.44(812.77-1002.7) | 125.83(109.98-143.77) | 11306.29(9685.76-13113.04) |
| Central Latin America | 10521.77(8375.1-12859.99) | 712.99(581.09-855.56) | 120.55(100.17-140.25) | 9025.49(7598.5-10525.73) |
| Central Sub-Saharan Africa | 1828.59(1397.78-2409.25) | 135.88(107.13-172.17) | 4.32(2.16-7.31) | 745.01(534.47-1046.71) |
| East Asia | 12964.59(9741.82-17334.78) | 743.59(561.68-983.35) | 26.69(19.33-35.37) | 5021.64(3549.56-6727.73) |
| Eastern Europe | 25334.98(22992.16-27753.92) | 1122.27(996.98-1259.34) | 220.13(181.13-267.77) | 17467.8(14613.21-20169.95) |
| Eastern Sub-Saharan Africa | 6419.55(4936.38-8357.71) | 478.58(377.82-602.12) | 14.03(5.99-21.44) | 2569.34(1780.61-3536.41) |
| High-income Asia Pacific | 5547.34(4368.73-7010.06) | 281.06(222.86-351.44) | 12.01(10.89-13.32) | 2151.1(1603.23-2808.74) |
| High-income North America | 154160.15(142274.27-167033.62) | 7898.96(7308.06-8528.45) | 300.78(286.27-316.19) | 53543.09(42526.98-65163.16) |
| North Africa and Middle East | 105835.24(89221.55-125353.33) | 6082.95(5180.76-7136.9) | 294.55(247.54-351.74) | 42139.31(33378.54-52327.7) |
| Oceania | 90.67(65.76-125.65) | 7.32(5.49-9.88) | 0(0-0.01) | 25.72(15.89-39.29) |
| South Asia | 58687.24(46283.71-72920.61) | 3949.53(3199.23-4812.96) | 74.62(51.72-98.74) | 20421.49(14893.51-27428.76) |
| Southeast Asia | 6897.2(5162.62-9283.41) | 458.89(350.28-597.73) | 32.41(26.67-38.77) | 3682.85(2918-4577.72) |
| Southern Latin America | 5602.2(4517.07-6754.73) | 315.86(261.6-383.07) | 19.73(17.19-22.36) | 2479.67(1936.14-3110.74) |
| Southern Sub-Saharan Africa | 2104.29(1659.34-2684.92) | 139.54(111.44-173.99) | 10.81(7.45-14.72) | 1113.51(851.38-1434.46) |
| Tropical Latin America | 14317.9(11345.67-17963.38) | 1082.4(886.46-1322.33) | 54.06(50.01-58.41) | 6561.82(5247.56-8101.56) |
| Western Europe | 146232.19(127665.89-167455.73) | 7343.94(6528.16-8244.75) | 389.81(365.1-414.06) | 56094.21(44458.59-68078.15) |
| Western Sub-Saharan Africa | 13230.15(10618.2-16347.48) | 1076(886.85-1291.28) | 343.14(209.32-498.55) | 26909.6(17714.7-37822.09) |
| 204 Countries |  |  |  |  |
| People's Republic of China | 11913.4(8926.84-16042.44) | 693.49(522.99-922.15) | 24.58(17.26-32.98) | 4617.83(3245.95-6228.61) |
| Democratic People's Republic of Korea | 404.17(303.89-522.79) | 24.73(19.08-31.32) | 0.87(0.36-1.73) | 158.77(109.17-230.21) |
| Taiwan (Province of China) | 647.02(485.73-834.15) | 25.37(19.62-32.31) | 1.24(1.01-1.51) | 245.05(174.64-315.03) |
| Kingdom of Cambodia | 161.91(117.87-220.85) | 12.14(9.15-16.09) | 0.48(0.19-0.88) | 72.19(47.92-106.51) |
| Republic of Indonesia | 2520.82(1859.96-3375.86) | 170.53(129.51-223.04) | 8.86(5.87-13.7) | 1185.85(863.32-1578.66) |
| Lao People's Democratic Republic | 77.86(57.97-105.34) | 5.99(4.57-8.02) | 0.22(0.09-0.42) | 34.36(22.62-51.66) |
| Malaysia | 259.09(192.07-352.28) | 17.35(13.06-23.51) | 2.37(1.44-3.74) | 197.88(138.1-270) |
| Republic of Maldives | 3.59(2.64-4.89) | 0.23(0.17-0.3) | 0.01(0-0.02) | 1.53(0.96-2.25) |
| Republic of the Philippines | 1090.31(807.96-1473.37) | 74.72(57.25-97.64) | 12.41(9.36-16.03) | 976.6(761.82-1221.83) |
| Republic of the Union of Myanmar | 662.93(498.11-888.95) | 48.21(37.25-62.85) | 2.09(0.87-3.8) | 302.02(204.89-427.36) |
| Democratic Socialist Republic of Sri Lanka | 212.35(162.05-285.28) | 13.44(10.36-17.47) | 0.33(0.18-0.56) | 77.46(54.44-108.12) |
| Kingdom of Thailand | 708.37(536.62-939.77) | 41.88(31.65-54.55) | 1.65(0.93-2.67) | 285.49(207.08-382.92) |
| Democratic Republic of Timor-Leste | 9.74(7.13-13.47) | 0.78(0.59-1.05) | 0.02(0.01-0.04) | 4.05(2.7-5.77) |
| Socialist Republic of Viet Nam | 1164.38(879.98-1539.59) | 71.94(55.29-92.86) | 3.81(1.31-7.5) | 529.8(348.49-782.13) |
| Republic of Fiji | 8.08(6.04-10.97) | 0.58(0.44-0.75) | 0(0-0) | 2.29(1.4-3.42) |
| Republic of Kiribati | 0.9(0.65-1.23) | 0.08(0.06-0.11) | 0(0-0) | 0.26(0.16-0.39) |
| Republic of the Marshall Islands | 0.45(0.33-0.61) | 0.04(0.03-0.05) | 0(0-0) | 0.13(0.08-0.19) |
| Federated States of Micronesia | 0.72(0.52-0.99) | 0.06(0.04-0.08) | 0(0-0) | 0.2(0.13-0.31) |
| Independent State of Papua New Guinea | 63.43(45.38-89.18) | 5.26(3.89-7.14) | 0(0-0.01) | 18(11.13-27.91) |
| Independent State of Samoa | 1.56(1.14-2.14) | 0.12(0.09-0.16) | 0(0-0) | 0.44(0.27-0.66) |
| Solomon Islands | 5.56(4.03-7.54) | 0.43(0.34-0.56) | 0(0-0) | 1.57(0.97-2.27) |
| Republic of Vanuatu | 2.52(1.86-3.45) | 0.2(0.16-0.27) | 0(0-0) | 0.71(0.44-1.08) |
| Kingdom of Tonga | 0.92(0.69-1.25) | 0.07(0.05-0.09) | 0(0-0) | 0.26(0.16-0.38) |
| Republic of Armenia | 221.18(172.88-280.31) | 20.35(16.94-24.14) | 0.24(0.19-0.3) | 71.5(49.58-96.21) |
| Republic of Azerbaijan | 509.41(397.22-639.82) | 39.88(31.97-49.01) | 0.42(0.18-0.82) | 161.36(104.02-224.99) |
| Georgia | 203(164.18-256.3) | 16.73(14.18-19.86) | 0.29(0.23-0.36) | 69.55(48.31-96.55) |
| Republic of Kazakhstan | 1668.01(1343.86-2125.55) | 185.63(162.33-219.21) | 5.41(3.54-7.41) | 708.79(492.21-965.13) |
| Kyrgyz Republic | 295.41(231.01-366.7) | 24.35(19.78-29.64) | 0.62(0.47-0.8) | 112.82(84.24-150.11) |
| Mongolia | 161.24(127.54-206.04) | 12.82(10.33-15.86) | 1.1(0.48-2.3) | 100.17(62.34-164.78) |
| Republic of Tajikistan | 368.92(289.32-469.65) | 31.16(25.02-38.19) | 0.55(0.16-1.35) | 132.52(88.89-194.84) |
| Turkmenistan | 277.49(217.09-349.93) | 28.42(24.46-33.61) | 1.2(0.63-2.05) | 137.81(90.12-194.96) |
| Republic of Uzbekistan | 1609.25(1265.25-2044.44) | 131.86(106.84-160.34) | 2.86(2.09-3.69) | 594.83(408.62-821.11) |
| Republic of Albania | 547.5(466.75-646.74) | 39.07(35.03-43.27) | 5.51(2.4-10.88) | 416.81(250.07-691.71) |
| Bosnia and Herzegovina | 330.1(288.92-375.12) | 17.74(15.75-20) | 2.15(1.16-3.82) | 192.17(134.25-274.71) |
| Republic of Bulgaria | 1127(957.93-1304.24) | 66.76(59.68-74.23) | 11.12(8.58-13.88) | 829.88(672.06-1018.62) |
| Republic of Croatia | 516.2(433.44-595.7) | 29.41(26.41-32.27) | 3.76(2.89-4.77) | 316.04(255.25-387) |
| Czech Republic | 1299.49(1127.76-1490.04) | 63.55(56.02-70.61) | 12.64(10.16-15.41) | 951.83(777.56-1153.84) |
| Hungary | 1393.74(1190.24-1596.14) | 61.9(54.72-69.25) | 12.04(9.31-15.06) | 936.01(765.17-1127.11) |
| North Macedonia | 351.29(299.45-407.69) | 20.23(17.97-22.52) | 2.24(1.39-3.54) | 200.22(147.68-267.42) |
| Montenegro | 101.45(86.63-115.35) | 5.62(5.12-6.1) | 0.81(0.44-1.38) | 65.7(44.46-96.49) |
| Republic of Poland | 9912.19(8918.07-10881.49) | 384.44(339.58-430.73) | 37.8(31.62-44.4) | 4413.44(3595.29-5249.19) |
| Romania | 1145.75(960.91-1364.59) | 56.92(47.72-67.16) | 12.48(9.76-16.09) | 909.67(712.11-1115.06) |
| Republic of Serbia | 2051.35(1793.14-2329.15) | 101.78(94-110.18) | 15.19(9.13-23.41) | 1267.77(879.57-1733.42) |
| Slovak Republic | 617.4(525.44-725.24) | 29.9(26.1-34.06) | 6.33(4.03-9.74) | 472.43(347.03-650.79) |
| Republic of Slovenia | 296.58(254.48-335.31) | 14.94(13.35-16.51) | 1.93(1.42-2.48) | 169.75(128.41-212.65) |
| Republic of Belarus | 666.62(559.34-804.31) | 41.37(35.51-47.98) | 9.01(6.74-11.64) | 620.88(493.08-771.52) |
| Republic of Estonia | 107.58(89.78-127.29) | 6.6(5.84-7.33) | 1.35(1.08-1.65) | 94.72(76.87-112.38) |
| Republic of Latvia | 170.07(145.45-201.65) | 10.75(9.65-11.84) | 3.08(2.47-3.8) | 192.3(158.22-232.56) |
| Republic of Lithuania | 220.25(186.57-257.02) | 14.24(12.76-15.72) | 3.98(3.12-4.89) | 250.82(202.91-302.04) |
| Republic of Moldova | 135.38(109.33-170.86) | 7.76(6.03-9.69) | 1.2(0.95-1.48) | 96.16(76.28-115.67) |
| Russian Federation | 18834.42(17328.24-20331.57) | 724.57(649.2-806.25) | 137.05(117.36-155.16) | 11638.35(10021.26-13393.97) |
| Ukraine | 5200.66(4447.44-6031.71) | 316.99(273.01-362.16) | 64.46(34.73-105.24) | 4574.57(2978.27-6610.29) |
| Brunei Darussalam | 8.32(6.44-10.67) | 0.52(0.41-0.66) | 0.03(0.01-0.09) | 3.78(2.12-6.84) |
| Japan | 3716.31(2938.92-4695.81) | 186.88(148.79-234.25) | 8.72(8.25-9.21) | 1470.2(1109.08-1915.76) |
| Republic of Korea | 1716.17(1355.45-2146.78) | 87.88(69.2-109.69) | 3.14(2.18-4.36) | 640.73(469.95-851.89) |
| Republic of Singapore | 106.54(81.67-139.23) | 5.77(4.41-7.38) | 0.13(0.11-0.15) | 36.39(25.71-50.22) |
| Australia | 6565.16(5493.24-7877.82) | 366.58(315.77-426.53) | 17.32(15.09-19.98) | 2500.54(1874.24-3222.9) |
| New Zealand | 760.3(637.65-902.95) | 46.23(40.06-52.81) | 3.96(3.25-4.77) | 385.34(304-466.24) |
| Republic of Austria | 2835.07(2400.85-3256.57) | 145.9(128.43-167.35) | 8.3(6.93-9.76) | 1127.15(835.98-1399.63) |
| Principality of Andorra | 32.09(27.64-38.01) | 1.44(1.23-1.67) | 0.12(0.06-0.24) | 13.99(9.59-20.72) |
| Kingdom of Belgium | 3158.23(2656.39-3800.21) | 162.26(138.32-187.18) | 11.81(10.1-13.79) | 1378.61(1078.73-1724.16) |
| Kingdom of Denmark | 2434.82(2070.86-2907.08) | 127.63(110.77-148.58) | 7.23(6.1-8.57) | 964.88(749.36-1207.63) |
| Republic of Cyprus | 291.05(235.84-354.09) | 14.7(12.16-17.64) | 0.75(0.33-1.48) | 111.86(76.9-159.14) |
| Republic of Finland | 1572.16(1401.03-1771.01) | 84.77(77.28-93.22) | 5.06(4.17-5.99) | 649.05(511.41-803.23) |
| French Republic | 22909.26(19966.07-26180.47) | 1037.3(912.46-1156.24) | 43.8(36.75-51.53) | 7989.57(5999.44-9941.67) |
| Federal Republic of Germany | 27408.24(23092.03-32258.59) | 1431.23(1241.52-1676.96) | 65.22(56.82-75) | 10079.77(7613.84-12902.26) |
| Republic of Iceland | 143.15(121.64-169.75) | 7.66(6.4-9.03) | 0.44(0.37-0.52) | 58.19(44.67-72.4) |
| Hellenic Republic | 1503.79(1252.49-1789.86) | 66.91(56.74-77.95) | 9.53(8.11-10.99) | 833.65(685.19-1033.69) |
| Ireland | 2772.12(2281.18-3383.23) | 122.25(104.48-144.34) | 6.94(5.61-8.33) | 1036.05(783.57-1300.43) |
| State of Israel | 1189.07(975.22-1441.46) | 62.35(51.43-74.44) | 2.48(2.08-2.92) | 434.04(320.68-581.68) |
| Republic of Italy | 17594.99(14806.07-20727.69) | 696.18(586.39-817.98) | 37.44(34.06-40.8) | 6287.84(4842.79-7954.97) |
| Grand Duchy of Luxembourg | 257.69(216.01-297.88) | 13.29(11.43-15.23) | 0.64(0.53-0.77) | 96.54(73.49-121.9) |
| Kingdom of Spain | 12800.96(11549.79-14113.07) | 508.75(454.27-563.33) | 22.09(18.74-26.08) | 4320.8(3220.03-5605.48) |
| Kingdom of the Netherlands | 6029.35(5034.91-7063.71) | 320.65(283.65-367.14) | 18.72(15.93-21.99) | 2443.56(1894.1-3051.74) |
| Portuguese Republic | 1197.57(1072.47-1331.67) | 57.58(51.67-64.11) | 5.4(4.46-6.43) | 576.5(465.69-705.02) |
| Republic of Malta | 40.93(33.45-48.9) | 2.04(1.68-2.41) | 0.22(0.18-0.27) | 21.25(16.89-26.65) |
| Swiss Confederation | 3449.73(2904.72-3961.98) | 185.12(159.23-211.94) | 10.41(8.81-12.08) | 1383.22(1080.91-1727.06) |
| Kingdom of Norway | 2552.78(2090.58-3058.41) | 150.78(128.75-176.43) | 6.36(5.76-7.02) | 954.49(743.61-1184.23) |
| United Kingdom of Great Britain and Northern Ireland | 30153.35(25845.64-35088.37) | 1821.26(1601.32-2060.61) | 117.27(111.72-123.15) | 13350.72(11017.91-15787.3) |
| Kingdom of Sweden | 5764.49(4826.68-6867.12) | 316.86(273.88-365.29) | 9.21(7.6-11.03) | 1928.3(1412.75-2473.15) |
| Argentine Republic | 3948.65(3196.46-4726.66) | 225.33(188.48-271.21) | 14.89(12.82-17.09) | 1796.37(1387.28-2272.75) |
| Eastern Republic of Uruguay | 296.88(246.22-357.25) | 16.56(14.02-19.71) | 2.05(1.7-2.42) | 181.34(143.8-223.46) |
| Republic of Chile | 1356.37(1072.76-1636.2) | 73.96(58.7-89.45) | 2.79(2.33-3.26) | 501.82(364.59-670.31) |
| Canada | 18747.21(17883.92-19594.69) | 1022.36(980.43-1067.81) | 37.69(32.59-42.62) | 6656.53(5229.73-8330.51) |
| United States of America | 135399.53(123679.53-147568.51) | 6876.1(6308.33-7475.45) | 263.08(249.21-277.68) | 46882.92(37089.69-57223.05) |
| Barbados | 28.87(23.93-34.45) | 1.85(1.62-2.09) | 0.4(0.29-0.53) | 27.64(21.27-34.75) |
| Belize | 14.71(11.5-18.31) | 1.02(0.81-1.23) | 0.13(0.1-0.16) | 10.63(8.82-12.86) |
| Republic of Cuba | 715.61(579.13-854.9) | 42.45(35.21-49.4) | 7.74(6.16-9.59) | 572.89(466.46-695.85) |
| Grenada | 5.38(4.34-6.5) | 0.37(0.3-0.43) | 0.09(0.07-0.11) | 5.99(4.79-7.32) |
| Dominican Republic | 385.19(301.34-479.3) | 26.12(20.82-31.96) | 2.39(1.37-3.99) | 231.97(161.59-327.34) |
| Commonwealth of Dominica | 2.08(1.63-2.59) | 0.14(0.11-0.17) | 0.01(0.01-0.02) | 1.35(0.94-1.96) |
| Commonwealth of the Bahamas | 23.49(19.09-28.73) | 1.48(1.22-1.77) | 0.51(0.37-0.68) | 32.38(24.7-41.74) |
| Antigua and Barbuda | 10.26(8.68-12.26) | 0.7(0.61-0.8) | 0.1(0.09-0.12) | 7.9(6.6-9.32) |
| Republic of Guyana | 19.62(15.51-24.7) | 1.39(1.11-1.7) | 0.21(0.13-0.32) | 16.57(12.11-22.43) |
| Republic of Haiti | 565.1(449.6-687.82) | 43.06(34.92-51.43) | 6.32(2.8-13.23) | 492.1(298.26-855.99) |
| Saint Vincent and the Grenadines | 4.49(3.59-5.49) | 0.29(0.23-0.34) | 0.07(0.06-0.09) | 4.88(4-5.94) |
| Jamaica | 104.74(82.35-131.38) | 6.9(5.51-8.54) | 1.16(0.8-1.64) | 88.62(67.49-116.59) |
| Republic of Suriname | 14.37(11.35-18.18) | 0.93(0.74-1.15) | 0.12(0.07-0.19) | 9.98(7.25-13.43) |
| Saint Lucia | 6.66(5.23-8.25) | 0.4(0.32-0.49) | 0.1(0.07-0.13) | 6.81(5.52-8.43) |
| Plurinational State of Bolivia | 455.97(354.73-572.57) | 35.08(28.31-42.82) | 2.56(0.9-5.76) | 255.18(162.6-421.51) |
| Republic of El Salvador | 165.53(127.23-208.78) | 11.24(9.02-13.85) | 1.34(0.83-2.01) | 114.81(85.08-156.35) |
| Republic of Trinidad and Tobago | 52.75(42.29-64.65) | 3.09(2.47-3.76) | 0.94(0.63-1.27) | 62.14(47.09-79.11) |
| Republic of Costa Rica | 215.74(168.03-268.88) | 15.63(12.78-18.72) | 2.93(2.35-3.48) | 208.23(171.76-245.06) |
| Republic of Peru | 1215.09(931.97-1547.24) | 88.29(67.57-109.67) | 5.62(3.04-9.83) | 618.78(449.37-845.13) |
| Republic of Ecuador | 438.31(339.48-567.95) | 31.42(24.82-39.37) | 2.89(2.06-3.92) | 267.41(206.8-339.05) |
| Republic of Colombia | 1310.81(1015.38-1674.78) | 89.11(70.53-110.22) | 16.3(12.81-20.3) | 1210.33(985.57-1465.77) |
| Republic of Panama | 108.81(83.99-136.84) | 7.28(5.78-9) | 1.61(1.2-2.07) | 113.82(89.76-140.41) |
| Bolivarian Republic of Venezuela | 875.56(679.52-1095.39) | 56.91(45.66-69.4) | 18.16(13.02-24.82) | 1170.49(898.5-1496.14) |
| Republic of Honduras | 258.06(199.8-324.82) | 18.93(14.83-23.23) | 0(0-0) | 72.72(46.85-103.05) |
| Republic of Nicaragua | 250.29(195.73-321.56) | 19.22(15.22-23.92) | 1.52(0.97-2.33) | 148.79(107.22-200.52) |
| Republic of Guatemala | 454.84(355.06-581) | 34.43(27.25-42.55) | 3.71(3-4.53) | 320.73(262.97-387.21) |
| United Mexican States | 6882.12(5516.65-8384.94) | 460.24(378.55-547.7) | 74.99(59.45-90.53) | 5665.57(4661.68-6720.58) |
| Republic of Paraguay | 325.91(256.63-411.87) | 24.08(19.17-29.85) | 1.24(0.78-1.93) | 152.75(112.02-206.57) |
| Arab Republic of Egypt | 14357.13(11663.63-17658.98) | 848.19(685.11-1038.6) | 2.59(1.41-4.26) | 3857.82(2457.13-5650.65) |
| Islamic Republic of Iran | 20326.63(16935.87-24028.68) | 1160.17(992.82-1351.81) | 99.79(79.72-123.13) | 10268.44(8303.39-12603.98) |
| Kingdom of Bahrain | 214.44(172.4-262.43) | 12.07(9.83-14.82) | 0.38(0.23-0.59) | 74.54(53.85-101.61) |
| Republic of Iraq | 5773.04(4612.25-7069.54) | 352.48(283.57-425.52) | 10.1(5.71-17.61) | 2001.16(1393.08-2753.9) |
| Lebanese Republic | 1440.7(1169.03-1823.3) | 78.36(64.2-97.91) | 1.43(0.84-2.33) | 439.31(306.87-620.65) |
| People's Democratic Republic of Algeria | 9212.48(7565.44-11252.97) | 512.47(429.19-617.24) | 28.84(16.34-48.09) | 3823.25(2703.06-5123.29) |
| Federative Republic of Brazil | 13991.99(11088.39-17569.31) | 1058.32(867.06-1293.31) | 52.82(48.81-57.24) | 6409.07(5109.71-7909.69) |
| Sultanate of Oman | 737.14(583.22-923.37) | 43.55(35.2-53.63) | 1.49(0.57-3.44) | 266.92(173.76-381.86) |
| Palestine | 817.16(641.96-998.68) | 52.95(42.51-64.35) | 2.54(1.66-3.84) | 342.35(254.24-442.45) |
| State of Kuwait | 1375.85(1123.59-1647.18) | 62.23(50.65-74.63) | 0.98(0.78-1.2) | 395.23(268.65-556.98) |
| State of Libya | 1556.47(1256.92-1917.6) | 83.91(70.94-102.23) | 11.04(5.61-20.56) | 947.26(624.17-1415.92) |
| Kingdom of Morocco | 7422.78(5970.94-9117.61) | 421.62(347.83-506.2) | 25.52(12.27-49.6) | 3182.4(2174.22-4726.13) |
| Hashemite Kingdom of Jordan | 2408.28(1939.23-2941.9) | 146.1(120.05-177.9) | 6.79(4.45-10.46) | 960.29(698.32-1287.82) |
| United Arab Emirates | 952.47(820.2-1125.42) | 40.67(33.17-48.2) | 0.8(0.45-1.39) | 291.15(202.15-394.46) |
| Republic of Tunisia | 2882.67(2342.94-3536.69) | 147.35(121.39-177.06) | 8.95(4.49-15.6) | 1179.79(812.54-1622.03) |
| State of Qatar | 816.43(723.52-921.56) | 44.36(37.93-52.08) | 0.28(0.16-0.46) | 221.48(152.67-305.64) |
| Republic of Turkey | 15437.63(14621.04-16260.04) | 830.11(781.3-880.44) | 40.19(25.19-59.17) | 5944.26(4416.13-7653.68) |
| Kingdom of Saudi Arabia | 5752.57(4536.01-7196.18) | 315.38(246.8-393.4) | 13.62(7.35-23.72) | 2171.65(1489.95-2952.03) |
| Syrian Arab Republic | 2493.2(2006.57-3027.86) | 134.56(110.92-163.04) | 4.05(2.18-7.51) | 840.82(570.27-1165.93) |
| Republic of India | 44196.15(34827.77-54989.91) | 2955.49(2388.06-3600.23) | 56.75(41.85-73.55) | 15395.71(11255.11-20655.67) |
| Republic of Yemen | 3050.15(2407.69-3789.6) | 199.79(161.3-243.67) | 6.59(3.36-12.8) | 1139.05(769.18-1629.17) |
| Islamic Republic of Pakistan | 8265.49(6536.59-10327.81) | 581.26(471.77-708.5) | 10.49(5.39-18.15) | 2885.65(2082.34-3888.87) |
| People's Republic of Bangladesh | 5066.69(3979.95-6469.86) | 332.45(265.85-417.59) | 6.29(1.78-13.27) | 1756.39(1216.43-2456.51) |
| Kingdom of Bhutan | 26.59(21.2-33.3) | 1.81(1.46-2.26) | 0.03(0.01-0.05) | 8.91(6.26-12.41) |
| Federal Democratic Republic of Nepal | 1132.32(882.21-1419.21) | 78.53(63.38-95.81) | 1.06(0.29-2.17) | 374.83(264-530.5) |
| Islamic Republic of Afghanistan | 4368.41(3596.06-5301.21) | 309.1(255.73-373.91) | 15.16(7.6-30.05) | 1901.46(1284.79-2783.02) |
| Democratic Republic of the Congo | 1110.45(836.08-1475.14) | 82.98(64.77-104.57) | 2.39(1.03-4.29) | 439.96(309.79-606.26) |
| Central African Republic | 77.51(58.84-101.56) | 5.99(4.71-7.63) | 0.14(0.05-0.27) | 29.15(20.59-41.02) |
| Republic of the Congo | 84.47(64.83-108.57) | 6.07(4.8-7.53) | 0.32(0.14-0.62) | 40.17(26.51-57.93) |
| Republic of Angola | 509.77(392.43-666.01) | 37.47(29.81-47.82) | 1.32(0.45-2.83) | 214.1(142.12-319.95) |
| Republic of Madagascar | 569.68(434.75-729.6) | 41.58(32.83-51.83) | 1.25(0.34-2.8) | 228.36(149.8-330.91) |
| Republic of Malawi | 320.18(242.53-415.58) | 24.58(19.28-31.35) | 0.71(0.15-1.49) | 128.94(80.68-192.99) |
| Republic of Kenya | 720.33(552.95-948.15) | 52.82(41.65-67.41) | 1.93(1.3-2.74) | 305.19(219.98-398.16) |
| State of Eritrea | 131.15(102.83-167.35) | 9.69(7.75-12.02) | 0.33(0.11-0.73) | 54.11(36.17-78.9) |
| Republic of Equatorial Guinea | 19.71(15.13-26.02) | 1.44(1.13-1.86) | 0.07(0.03-0.14) | 9.34(6.26-13.64) |
| Gabonese Republic | 26.68(20.26-34.41) | 1.92(1.51-2.43) | 0.09(0.05-0.18) | 12.29(8.41-17.15) |
| Federal Democratic Republic of Ethiopia | 1623.43(1243.56-2108.47) | 119.64(94.63-150.64) | 3.39(1.02-6.02) | 642.13(423.36-892.27) |
| Union of the Comoros | 14.81(11.58-19.22) | 1.02(0.81-1.26) | 0.04(0.02-0.08) | 6.47(4.43-9.2) |
| Republic of Burundi | 161.66(121.39-212.9) | 12.46(9.7-16.02) | 0.26(0.06-0.52) | 60.16(40.16-83.76) |
| Republic of Djibouti | 25.64(19.9-32.68) | 1.71(1.35-2.16) | 0.06(0.02-0.12) | 10.23(6.96-14.96) |
| Kingdom of Lesotho | 45.31(34.98-57.82) | 3.33(2.65-4.16) | 0.04(0.02-0.07) | 14.65(10.12-20.75) |
| Republic of Seychelles | 1.74(1.33-2.19) | 0.12(0.1-0.15) | 0.01(0-0.01) | 0.81(0.57-1.13) |
| United Republic of Tanzania | 876.21(669.64-1145.61) | 64.8(51.77-81.98) | 1.97(0.72-3.77) | 353.24(244.85-502.31) |
| Republic of Mozambique | 548.38(421.72-704.66) | 42.33(33.68-53.42) | 1.16(0.17-2.64) | 217.85(134.56-332.36) |
| Kingdom of Eswatini | 26.58(20.13-34.34) | 1.89(1.51-2.36) | 0.03(0.01-0.05) | 8.83(6.23-12.37) |
| Republic of Zimbabwe | 294.09(228.29-375.49) | 21.45(17.04-26.86) | 0(0-0) | 82.87(52.39-116.44) |
| Republic of Rwanda | 191.62(143.22-253.53) | 13.81(10.82-17.75) | 0.47(0.18-0.84) | 79.01(54.78-112.73) |
| Republic of South Africa | 1627.33(1287.6-2072.78) | 105.4(84.07-131.39) | 10.65(7.34-14.52) | 971.15(740.94-1248.49) |
| Republic of Mauritius | 14.49(10.89-19.41) | 0.9(0.69-1.16) | 0.11(0.09-0.13) | 9.67(7.84-11.84) |
| Republic of Uganda | 488.78(364.32-645.53) | 37.59(28.67-48) | 0.98(0.31-1.91) | 191.8(128.29-269.12) |
| Republic of Namibia | 51.78(39.88-67.27) | 3.55(2.76-4.55) | 0.05(0.02-0.09) | 17.03(11.56-23.57) |
| Republic of Zambia | 346.03(266.6-444.78) | 26.54(21.09-33.5) | 0.97(0.43-1.85) | 150.68(107.25-212.7) |
| Federal Republic of Somalia | 262.59(197.83-344.76) | 20.04(15.49-25.21) | 0.28(0.04-0.65) | 88.92(59.32-128.6) |
| Republic of Botswana | 59.21(46.19-76) | 3.92(3.12-5.03) | 0.04(0.02-0.09) | 18.98(12.92-26.65) |
| Republic of Chad | 373.95(288.96-476.42) | 30.57(24.42-37.73) | 8.23(1.69-22.73) | 662.82(221.99-1623.67) |
| Republic of Guinea-Bissau | 57.09(45.12-70.88) | 4.62(3.7-5.59) | 1.87(0.63-4.64) | 142.45(59.07-331.71) |
| Republic of Ghana | 2812.93(2190.12-3438.47) | 266.3(221.23-306.44) | 35.4(11.02-87.84) | 3143.27(1429.48-6821.83) |
| Federal Republic of Nigeria | 5226.97(4084.57-6538.99) | 400.01(322.99-491.26) | 153.7(81.94-273.6) | 11877.11(6986.42-20086.09) |
| Democratic Republic of Sao Tome and Principe | 4.32(3.38-5.47) | 0.32(0.26-0.39) | 0.04(0.01-0.1) | 3.85(1.86-7.8) |
| Republic of the Niger | 522.28(411.76-652.6) | 42.78(34.42-51.88) | 10.46(1.21-28.57) | 855.47(222.6-2095.12) |
| Republic of the Gambia | 64.49(51.25-80.5) | 5.12(4.13-6.23) | 2.4(0.68-5.92) | 180.84(60.7-418.78) |
| Islamic Republic of Mauritania | 140.9(112.45-175.17) | 10.75(8.85-13) | 4.08(1.3-9.92) | 315.69(125.76-713.28) |
| Republic of Côte d'Ivoire | 587.79(454.11-743.5) | 45.09(35.97-55.13) | 19.19(5.53-53.01) | 1464.08(541.51-3743.08) |
| Republic of Guinea | 320.56(252.28-409.93) | 25.49(20.51-32.01) | 9.22(2.74-23.82) | 714.29(274.58-1701.27) |
| Republic of Cameroon | 705.21(556.53-893.21) | 56.06(45.07-69.06) | 25.72(7.79-66.35) | 1939.87(697.85-4702.79) |
| Republic of Cabo Verde | 18.92(15.02-23.42) | 1.27(1.02-1.56) | 0.34(0.08-0.95) | 28.65(10.51-69.52) |
| Burkina Faso | 523.09(409.94-659.87) | 41.44(33.27-50.79) | 13.62(3.18-39.45) | 1069.23(362.78-2804.46) |
| Republic of Benin | 299.02(235.04-374.62) | 23.31(18.58-28.69) | 8.75(2.6-22.14) | 676.62(254.17-1563.11) |
| Republic of Liberia | 125.46(97.21-159.04) | 9.37(7.57-11.46) | 4.36(1.26-10.81) | 330.61(121.72-778.27) |
| Republic of Mali | 597.72(469.2-735.31) | 48.91(39.46-59.11) | 19.56(4.38-47.65) | 1492.63(463.55-3407.62) |
| Cook Islands | 0.17(0.13-0.22) | 0.01(0.01-0.01) | 0(0-0) | 0.05(0.03-0.07) |
| American Samoa | 0.38(0.29-0.51) | 0.03(0.02-0.03) | 0(0-0) | 0.11(0.07-0.16) |
| Bermuda | 4.19(3.4-5.07) | 0.23(0.18-0.27) | 0.03(0.03-0.04) | 2.82(2.24-3.5) |
| Republic of Sierra Leone | 199.18(152.96-252.79) | 15.81(12.52-19.65) | 6.31(1.8-15.97) | 483.37(175.32-1131.4) |
| Togolese Republic | 217.22(171.37-270.4) | 15.95(12.92-19.48) | 6.35(1.93-14.8) | 490.77(189.01-1068.07) |
| Republic of Senegal | 432.89(339.18-540.16) | 32.81(26.34-40.11) | 13.53(3.8-36.95) | 1037.7(383.14-2623.16) |
| United States Virgin Islands | 3.81(3.02-4.66) | 0.23(0.19-0.27) | 0.05(0.02-0.1) | 3.56(2.08-6.02) |
| Tuvalu | 0.08(0.06-0.11) | 0.01(0-0.01) | 0(0-0) | 0.02(0.01-0.03) |
| Puerto Rico | 167.46(135.23-202.84) | 10(8.25-11.93) | 2.26(1.69-2.94) | 156.68(123.15-196.58) |
| Republic of San Marino | 4.74(3.83-5.84) | 0.21(0.17-0.25) | 0(0-0) | 1.24(0.77-1.86) |
| Tokelau | 0.01(0.01-0.01) | 0(0-0) | 0(0-0) | 0(0-0) |
| Republic of Palau | 0.12(0.09-0.16) | 0.01(0.01-0.01) | 0(0-0) | 0.03(0.02-0.05) |
| Saint Kitts and Nevis | 4.04(3.27-4.92) | 0.27(0.24-0.32) | 0.04(0.03-0.06) | 3.28(2.49-4.19) |
| Greenland | 10.99(8.96-13.27) | 0.38(0.32-0.46) | 0(0-0) | 2.8(1.8-3.93) |
| Northern Mariana Islands | 0.44(0.33-0.58) | 0.03(0.02-0.04) | 0(0-0) | 0.12(0.08-0.18) |
| Republic of Nauru | 0.07(0.05-0.09) | 0.01(0-0.01) | 0(0-0) | 0.02(0.01-0.03) |
| Guam | 1.18(0.89-1.61) | 0.08(0.06-0.1) | 0(0-0) | 0.34(0.21-0.49) |
| Republic of Niue | 0.02(0.01-0.02) | 0(0-0) | 0(0-0) | 0(0-0.01) |
| Principality of Monaco | 7.79(6.45-9.24) | 0.36(0.3-0.42) | 0.03(0.02-0.06) | 3.55(2.43-5.19) |
| Republic of Sudan | 4340.9(3438.6-5432.42) | 281.86(224.64-349.64) | 13.13(5.8-25.42) | 1851.4(1263.27-2601.63) |
| Republic of South Sudan | 133.49(102.53-173.67) | 9.55(7.53-12.32) | 0.23(0.05-0.53) | 50(33.24-73.41) |

MS, Multiple sclerosis; SDI, socio-demographic index.

Supplementary Table 2: ASIR, ASPR, ASMR, and ASDR of MS in Women of Childbearing Age in 2021, Globally and by Country

| location | Prevalence (95%UI) | Incidence (95%UI) | Deaths (95%UI) | DALYs (95%UI) |
| --- | --- | --- | --- | --- |
| Global | 29.32(26.24-32.74) | 0.98(0.88-1.1) | 0.23(0.21-0.24) | 14.54(12.37-16.93) |
| Low SDI | 10.4(8.58-12.26) | 0.49(0.42-0.58) | 0.05(0.03-0.08) | 5.6(3.95-7.46) |
| Low-middle SDI | 13.94(11.62-16.57) | 0.63(0.53-0.73) | 0.05(0.04-0.06) | 5.98(4.8-7.56) |
| Middle SDI | 12.53(10.58-14.74) | 0.58(0.5-0.67) | 0.08(0.07-0.08) | 6.15(5.11-7.4) |
| High-middle SDI | 26.02(23.48-28.95) | 0.96(0.88-1.06) | 0.18(0.16-0.2) | 12.86(10.89-14.86) |
| High SDI | 96.02(87.63-104.92) | 3.45(3.17-3.77) | 0.69(0.64-0.73) | 44.54(37.45-51.42) |
| 21 Regions |  |  |  |  |
| Andean Latin America | 12.61(10.3-15) | 0.55(0.46-0.66) | 0.12(0.08-0.15) | 7.21(5.75-8.98) |
| Australasia | 82.09(70.64-95) | 2.95(2.59-3.35) | 0.58(0.5-0.66) | 38.08(30.85-45.73) |
| Caribbean | 16.58(13.85-19.56) | 0.71(0.6-0.82) | 0.25(0.22-0.3) | 13.76(11.68-16.49) |
| Central Asia | 29.54(26.12-33.69) | 1.32(1.17-1.5) | 0.13(0.1-0.17) | 10.57(8.19-13.15) |
| Central Europe | 51.78(46.78-56.77) | 2.02(1.86-2.21) | 0.64(0.57-0.72) | 35.47(30.99-40.31) |
| Central Latin America | 14.14(11.76-16.82) | 0.63(0.53-0.73) | 0.23(0.2-0.26) | 12.14(10.33-14) |
| Central Sub-Saharan Africa | 5.35(4.28-6.64) | 0.28(0.23-0.34) | 0.03(0.01-0.04) | 2.43(1.79-3.29) |
| East Asia | 2.72(2.13-3.44) | 0.18(0.14-0.21) | 0.01(0.01-0.01) | 1.08(0.79-1.4) |
| Eastern Europe | 34.62(31.76-37.75) | 1.34(1.23-1.47) | 0.37(0.32-0.44) | 23.71(20.25-27.31) |
| Eastern Sub-Saharan Africa | 5.95(4.79-7.27) | 0.31(0.25-0.37) | 0.03(0.01-0.04) | 2.54(1.75-3.38) |
| High-income Asia Pacific | 11.71(9.61-14.19) | 0.46(0.38-0.56) | 0.04(0.03-0.04) | 4.57(3.49-5.9) |
| High-income North America | 145.65(135.61-156.47) | 4.94(4.6-5.34) | 0.99(0.92-1.05) | 64.29(54.04-74.71) |
| North Africa and Middle East | 61.73(53.46-71) | 2.1(1.82-2.42) | 0.23(0.19-0.26) | 23.4(18.99-28.51) |
| Oceania | 1.94(1.48-2.53) | 0.17(0.13-0.21) | 0(0-0) | 0.55(0.36-0.8) |
| South Asia | 10.42(8.58-12.43) | 0.5(0.42-0.59) | 0.02(0.02-0.03) | 3.74(2.81-4.91) |
| Southeast Asia | 2.82(2.2-3.58) | 0.2(0.16-0.24) | 0.02(0.01-0.02) | 1.52(1.22-1.85) |
| Southern Latin America | 26.18(21.6-30.89) | 0.99(0.84-1.18) | 0.17(0.15-0.19) | 12.7(10.31-15.24) |
| Southern Sub-Saharan Africa | 8.93(7.4-10.72) | 0.42(0.35-0.49) | 0.12(0.09-0.15) | 6.26(5.11-7.72) |
| Tropical Latin America | 25.55(21.7-30.12) | 1.06(0.9-1.22) | 0.15(0.14-0.16) | 11.28(9.14-13.61) |
| Western Europe | 121.71(108.47-137.09) | 4.34(3.9-4.85) | 0.86(0.78-0.91) | 56.53(47.25-65.46) |
| Western Sub-Saharan Africa | 11.54(9.84-13.55) | 0.59(0.51-0.68) | 0.12(0.07-0.17) | 10.99(7.78-14.82) |
| 204 Countries |  |  |  |  |
| People's Republic of China | 2.59(2.03-3.3) | 0.17(0.14-0.21) | 0.01(0.01-0.01) | 1.03(0.75-1.34) |
| Democratic People's Republic of Korea | 4.3(3.43-5.38) | 0.28(0.23-0.33) | 0.01(0.01-0.02) | 1.75(1.26-2.5) |
| Taiwan (Province of China) | 7.96(6.22-9.86) | 0.33(0.27-0.4) | 0.02(0.02-0.03) | 3.1(2.3-3.93) |
| Kingdom of Cambodia | 2.65(2.04-3.37) | 0.21(0.17-0.26) | 0.01(0.01-0.02) | 1.25(0.87-1.8) |
| Lao People's Democratic Republic | 2.89(2.29-3.7) | 0.24(0.19-0.29) | 0.01(0.01-0.02) | 1.33(0.91-1.92) |
| Malaysia | 2.41(1.9-3.05) | 0.16(0.13-0.2) | 0.03(0.02-0.05) | 1.95(1.47-2.51) |
| Republic of Indonesia | 2.44(1.89-3.12) | 0.18(0.15-0.22) | 0.01(0.01-0.02) | 1.19(0.9-1.58) |
| Republic of Maldives | 2.3(1.78-2.94) | 0.15(0.12-0.18) | 0.01(0-0.02) | 0.99(0.69-1.35) |
| Republic of the Union of Myanmar | 3.19(2.51-4.07) | 0.25(0.2-0.3) | 0.01(0.01-0.02) | 1.49(1.03-2.08) |
| Republic of the Philippines | 2.92(2.3-3.68) | 0.2(0.17-0.24) | 0.04(0.03-0.06) | 2.64(2.1-3.28) |
| Democratic Socialist Republic of Sri Lanka | 2.92(2.31-3.68) | 0.19(0.15-0.23) | 0.01(0-0.01) | 1.06(0.76-1.4) |
| Kingdom of Thailand | 3.1(2.42-3.94) | 0.2(0.16-0.24) | 0.01(0-0.01) | 1.21(0.9-1.59) |
| Democratic Republic of Timor-Leste | 2.37(1.85-3.06) | 0.19(0.15-0.23) | 0.01(0-0.02) | 1.02(0.7-1.4) |
| Socialist Republic of Viet Nam | 3.36(2.67-4.19) | 0.22(0.18-0.26) | 0.02(0.01-0.03) | 1.58(1.07-2.19) |
| Republic of Fiji | 2.56(1.99-3.29) | 0.2(0.16-0.24) | 0(0-0) | 0.72(0.47-1.01) |
| Republic of Kiribati | 2.11(1.62-2.69) | 0.22(0.18-0.26) | 0(0-0) | 0.6(0.39-0.86) |
| Republic of the Marshall Islands | 2.22(1.72-2.81) | 0.2(0.16-0.24) | 0(0-0) | 0.63(0.42-0.89) |
| Federated States of Micronesia | 2.11(1.61-2.7) | 0.18(0.15-0.22) | 0(0-0) | 0.6(0.39-0.86) |
| Independent State of Samoa | 2.56(1.98-3.23) | 0.19(0.16-0.24) | 0(0-0) | 0.72(0.47-1.03) |
| Independent State of Papua New Guinea | 1.75(1.3-2.3) | 0.16(0.13-0.2) | 0(0-0) | 0.5(0.32-0.73) |
| Solomon Islands | 2.55(1.97-3.24) | 0.2(0.16-0.23) | 0(0-0) | 0.72(0.48-1.02) |
| Kingdom of Tonga | 2.91(2.26-3.68) | 0.2(0.16-0.25) | 0(0-0) | 0.82(0.55-1.16) |
| Republic of Vanuatu | 2.42(1.89-3.12) | 0.21(0.17-0.26) | 0(0-0) | 0.69(0.44-0.97) |
| Republic of Armenia | 36.77(32.24-41.89) | 1.64(1.45-1.84) | 0.17(0.14-0.21) | 12.18(9.34-15.62) |
| Republic of Azerbaijan | 19.75(16.44-23.54) | 0.88(0.73-1.05) | 0.05(0.02-0.08) | 6.27(4.62-8.5) |
| Georgia | 27.45(24.03-31.51) | 1.22(1.06-1.39) | 0.13(0.1-0.15) | 9.72(7.34-12.59) |
| Republic of Kazakhstan | 55.22(49.08-63.04) | 2.56(2.3-2.9) | 0.22(0.14-0.3) | 18.98(13.75-24.16) |
| Kyrgyz Republic | 20.12(16.87-23.81) | 0.91(0.76-1.07) | 0.1(0.08-0.13) | 7.52(5.66-9.73) |
| Mongolia | 19.64(16.27-23.16) | 0.92(0.77-1.09) | 0.38(0.19-0.72) | 13.1(8.36-20.55) |
| Republic of Tajikistan | 17.09(14.34-20.4) | 0.81(0.67-0.95) | 0.06(0.03-0.1) | 5.9(4.31-8.05) |
| Turkmenistan | 32.71(28.95-37.17) | 1.56(1.39-1.75) | 0.23(0.13-0.36) | 13.96(9.47-18.38) |
| Republic of Uzbekistan | 20.12(16.88-23.65) | 0.93(0.78-1.08) | 0.09(0.07-0.11) | 7.27(5.53-9.37) |
| Republic of Albania | 82.79(74.03-93.15) | 3.3(2.99-3.63) | 1.16(0.52-2.09) | 60.32(38.28-93.95) |
| Bosnia and Herzegovina | 33.1(29.6-36.7) | 1.34(1.21-1.48) | 0.36(0.2-0.65) | 21.32(14.63-31.44) |
| Republic of Bulgaria | 56.81(50.32-63.16) | 2.38(2.17-2.6) | 0.75(0.6-0.93) | 42.33(35.39-50.42) |
| Republic of Croatia | 40.74(35.96-45.73) | 1.74(1.59-1.91) | 0.57(0.47-0.7) | 29.81(24.73-35.54) |
| Czech Republic | 36.6(32.21-40.96) | 1.55(1.41-1.7) | 0.69(0.59-0.81) | 32.81(27.92-38.06) |
| Hungary | 42.79(37.22-48.42) | 1.71(1.55-1.9) | 0.65(0.54-0.78) | 34.11(28.69-40.41) |
| North Macedonia | 50.29(44.25-56.33) | 1.99(1.79-2.19) | 0.55(0.35-0.82) | 31.35(23.84-40.77) |
| Montenegro | 51.14(45.27-57.06) | 2.11(1.94-2.29) | 0.69(0.44-1.02) | 37.05(26.82-50.96) |
| Republic of Poland | 72.99(66.33-79.3) | 2.68(2.45-2.94) | 0.73(0.62-0.83) | 42.83(36.4-49.19) |
| Romania | 18.67(15.96-21.9) | 0.82(0.71-0.95) | 0.33(0.27-0.4) | 16.83(13.9-19.66) |
| Republic of Serbia | 69.05(62.12-77.17) | 2.72(2.53-2.93) | 0.91(0.6-1.35) | 49.54(35.96-66.54) |
| Slovak Republic | 32.05(28.1-36.71) | 1.35(1.21-1.52) | 0.58(0.39-0.84) | 28.91(21.56-38.73) |
| Republic of Slovenia | 47.17(41.89-52.36) | 1.93(1.77-2.11) | 0.68(0.55-0.85) | 34.18(27.54-41.01) |
| Republic of Belarus | 21.92(19.07-25.37) | 0.99(0.87-1.13) | 0.32(0.24-0.4) | 18.39(14.78-22.63) |
| Republic of Estonia | 24.96(21.65-28.62) | 1.22(1.1-1.37) | 0.42(0.35-0.5) | 22.58(18.72-26.39) |
| Republic of Latvia | 27.21(23.7-31.24) | 1.41(1.28-1.55) | 0.63(0.52-0.76) | 31.55(26.09-37.45) |
| Republic of Lithuania | 25.27(22.08-28.9) | 1.29(1.17-1.41) | 0.6(0.48-0.72) | 29.56(24.17-35.5) |
| Republic of Moldova | 9.93(8.11-12.23) | 0.48(0.39-0.57) | 0.1(0.09-0.12) | 6.92(5.61-8.25) |
| Russian Federation | 37.04(34.31-39.91) | 1.3(1.2-1.41) | 0.36(0.31-0.41) | 23.54(20.33-26.89) |
| Ukraine | 32.63(28.64-37.04) | 1.61(1.44-1.81) | 0.41(0.24-0.65) | 26.24(17.66-36.86) |
| Brunei Darussalam | 5.59(4.59-6.88) | 0.26(0.21-0.31) | 0.03(0.01-0.09) | 2.7(1.55-4.99) |
| Japan | 12(9.85-14.56) | 0.47(0.39-0.57) | 0.04(0.04-0.04) | 4.82(3.73-6.15) |
| Republic of Korea | 11.7(9.59-14.13) | 0.47(0.38-0.57) | 0.03(0.02-0.04) | 4.3(3.22-5.68) |
| Republic of Singapore | 5.64(4.55-7.05) | 0.24(0.19-0.28) | 0.01(0.01-0.01) | 2.03(1.51-2.65) |
| Australia | 87.95(75.54-102.47) | 3.13(2.73-3.6) | 0.55(0.47-0.63) | 38.89(31.28-47.31) |
| Principality of Andorra | 113.17(98.37-133.78) | 4.08(3.58-4.7) | 0.88(0.48-1.39) | 56.26(39.86-77.58) |
| New Zealand | 51.81(44.81-60.35) | 2.06(1.82-2.35) | 0.7(0.58-0.83) | 33.91(28.08-39.49) |
| Kingdom of Belgium | 101.35(86.89-118.75) | 3.61(3.14-4.12) | 0.86(0.75-0.98) | 52.19(42.94-61.93) |
| Republic of Finland | 112.3(102.62-124.06) | 4(3.69-4.37) | 0.85(0.72-0.98) | 54.67(45.92-64.15) |
| Kingdom of Denmark | 150.5(130.38-178.63) | 5.36(4.73-6.22) | 1.3(1.11-1.48) | 76.47(63.91-90.78) |
| French Republic | 125.3(110.22-141.87) | 4.13(3.66-4.6) | 0.65(0.55-0.75) | 51.39(41.24-61.72) |
| Republic of Austria | 111.01(96.81-126.89) | 3.98(3.56-4.52) | 0.89(0.78-1.01) | 54.67(44.41-64.78) |
| Hellenic Republic | 49.15(41.85-58.36) | 1.82(1.6-2.11) | 0.62(0.54-0.71) | 32.7(27.81-38.55) |
| Republic of Cyprus | 63.11(52.93-74.98) | 2.22(1.9-2.59) | 0.41(0.27-0.64) | 28.47(21.65-37.21) |
| Federal Republic of Germany | 124.01(106.79-146.29) | 4.48(3.96-5.23) | 0.99(0.87-1.09) | 59.94(49.7-71.86) |
| State of Israel | 46.21(38.68-54.78) | 1.62(1.37-1.9) | 0.25(0.21-0.28) | 19.46(15.42-23.98) |
| Grand Duchy of Luxembourg | 125.4(107.21-144.1) | 4.53(3.98-5.1) | 0.8(0.69-0.92) | 56.08(45.64-66.79) |
| Republic of Iceland | 152.3(130.61-177.87) | 5.32(4.59-6.18) | 0.93(0.79-1.07) | 67.68(55.09-81.8) |
| Kingdom of Norway | 179.92(152.28-210.66) | 6.55(5.62-7.57) | 1.18(1.06-1.29) | 79.57(65.17-94.55) |
| Kingdom of Spain | 95.71(87.15-104.33) | 3.17(2.9-3.45) | 0.34(0.29-0.38) | 34.74(27.42-42.73) |
| Republic of Italy | 109.57(94.78-128.56) | 3.57(3.11-4.16) | 0.54(0.48-0.6) | 44.28(35.77-53.28) |
| Swiss Confederation | 134.65(117.64-152.94) | 4.92(4.4-5.55) | 1.1(0.95-1.27) | 66.46(54.78-78.16) |
| Ireland | 179.41(151.68-216.98) | 6.02(5.24-7.11) | 1.02(0.84-1.2) | 76.81(61.06-92.08) |
| Kingdom of Sweden | 227.48(197.12-262.66) | 7.81(6.88-8.89) | 0.98(0.82-1.16) | 85.33(67.26-104.65) |
| Portuguese Republic | 39.72(36.33-43.42) | 1.47(1.36-1.6) | 0.31(0.27-0.35) | 20.49(16.99-24.2) |
| Kingdom of the Netherlands | 132.31(114.8-153.63) | 4.75(4.21-5.43) | 1.03(0.9-1.16) | 64.8(53.36-76.11) |
| Republic of Malta | 33.36(28-39.33) | 1.25(1.06-1.44) | 0.35(0.29-0.41) | 19.87(16.69-23.57) |
| United Kingdom of Great Britain and Northern Ireland | 158.75(136.77-181.47) | 5.98(5.27-6.73) | 1.64(1.53-1.72) | 89.22(76.96-101.76) |
| Eastern Republic of Uruguay | 28.18(23.77-33.45) | 1.09(0.94-1.28) | 0.34(0.29-0.39) | 19.33(15.82-22.81) |
| United States of America | 141.13(130.16-152.97) | 4.77(4.39-5.21) | 0.99(0.91-1.05) | 62.87(52.82-72.87) |
| Argentine Republic | 27.06(22.48-31.68) | 1.04(0.89-1.22) | 0.19(0.17-0.22) | 13.71(11.2-16.37) |
| Antigua and Barbuda | 35.1(30.81-40.25) | 1.54(1.38-1.71) | 0.56(0.48-0.63) | 28.84(24.86-33.18) |
| Commonwealth of the Bahamas | 17.91(14.87-21.54) | 0.8(0.69-0.93) | 0.45(0.34-0.57) | 22.73(17.86-28.38) |
| Barbados | 31.96(27.52-36.97) | 1.41(1.25-1.57) | 0.62(0.46-0.8) | 30.99(24.42-38.51) |
| Canada | 184.21(178.22-190.85) | 6.44(6.24-6.66) | 1.02(0.9-1.16) | 76.5(62.98-90.82) |
| Republic of Chile | 23.8(19.35-28.42) | 0.87(0.72-1.03) | 0.09(0.08-0.1) | 9.24(7.1-11.79) |
| Republic of Cuba | 22.61(19.02-26.63) | 0.96(0.82-1.1) | 0.37(0.3-0.44) | 19.1(16.07-22.57) |
| Commonwealth of Dominica | 11.24(9.1-13.47) | 0.5(0.41-0.6) | 0.11(0.07-0.16) | 7.25(5.43-9.74) |
| Republic of Haiti | 13.67(11.37-16.17) | 0.72(0.62-0.83) | 0.23(0.1-0.47) | 12.58(7.49-21.7) |
| Republic of Suriname | 8.55(6.98-10.34) | 0.4(0.32-0.48) | 0.08(0.05-0.12) | 5.63(4.2-7.36) |
| Saint Lucia | 11.94(9.73-14.43) | 0.52(0.43-0.63) | 0.21(0.16-0.26) | 11.31(9.22-13.65) |
| Republic of Trinidad and Tobago | 12.01(9.78-14.45) | 0.53(0.44-0.62) | 0.25(0.17-0.33) | 13.22(10.16-16.84) |
| Grenada | 18.19(15.06-21.39) | 0.83(0.71-0.96) | 0.41(0.34-0.49) | 20.4(16.92-24.02) |
| Republic of Guyana | 8.76(7.14-10.58) | 0.43(0.35-0.51) | 0.12(0.08-0.17) | 7.17(5.39-9.4) |
| Saint Vincent and the Grenadines | 13.34(11.03-15.82) | 0.61(0.52-0.71) | 0.25(0.2-0.3) | 13.45(11.16-15.94) |
| Jamaica | 12.29(10.03-14.93) | 0.53(0.44-0.64) | 0.18(0.13-0.24) | 10.17(7.91-12.89) |
| Belize | 11.68(9.5-13.9) | 0.52(0.43-0.61) | 0.13(0.11-0.16) | 8.39(6.98-10.03) |
| Dominican Republic | 12.23(9.94-14.59) | 0.55(0.45-0.65) | 0.09(0.05-0.13) | 6.83(4.95-9.14) |
| Republic of Peru | 13.26(10.67-15.87) | 0.57(0.46-0.67) | 0.1(0.06-0.16) | 7(5.35-9.04) |
| Republic of Honduras | 8.97(7.35-10.75) | 0.43(0.36-0.51) | 0(0-0) | 2.53(1.71-3.48) |
| Republic of Ecuador | 9.6(7.82-11.72) | 0.43(0.35-0.51) | 0.12(0.09-0.15) | 6.35(4.97-7.8) |
| Republic of Costa Rica | 16.11(13.44-19.07) | 0.7(0.6-0.81) | 0.29(0.24-0.33) | 14.6(12.37-16.77) |
| Bolivarian Republic of Venezuela | 10.79(8.83-12.83) | 0.5(0.42-0.58) | 0.31(0.23-0.41) | 14.42(11.31-18.47) |
| Republic of Guatemala | 10.82(8.89-13.05) | 0.51(0.43-0.61) | 0.12(0.1-0.14) | 7.43(6.16-8.91) |
| United Mexican States | 17.72(14.79-20.82) | 0.77(0.67-0.9) | 0.27(0.22-0.33) | 14.58(12.15-17.22) |
| Plurinational State of Bolivia | 15.34(12.92-18.13) | 0.7(0.6-0.82) | 0.16(0.09-0.28) | 9.37(6.66-13.23) |
| Republic of Nicaragua | 14.9(12.43-17.96) | 0.66(0.55-0.78) | 0.12(0.09-0.18) | 8.48(6.45-10.94) |
| Republic of Panama | 9.66(7.92-11.62) | 0.42(0.35-0.51) | 0.19(0.15-0.24) | 9.78(7.81-11.9) |
| Republic of El Salvador | 9.02(7.4-10.9) | 0.4(0.33-0.48) | 0.1(0.07-0.14) | 6.18(4.65-8.05) |
| Republic of Colombia | 9.33(7.61-11.24) | 0.42(0.34-0.49) | 0.16(0.13-0.2) | 8.51(7.07-10.23) |
| State of Kuwait | 72.72(61.38-85.7) | 2.34(1.99-2.76) | 0.07(0.06-0.09) | 20.51(14.47-27.69) |
| Lebanese Republic | 81.78(68.36-101.22) | 2.75(2.31-3.37) | 0.1(0.07-0.14) | 23.95(18-32.41) |
| Hashemite Kingdom of Jordan | 76.8(63.68-92.29) | 2.57(2.13-3.08) | 0.29(0.21-0.4) | 29.52(22.36-38.05) |
| Arab Republic of Egypt | 52.9(43.96-63.65) | 1.81(1.5-2.17) | 0.02(0.01-0.02) | 13.76(9.26-19.72) |
| Republic of Iraq | 54.42(44.69-64.81) | 1.85(1.51-2.2) | 0.11(0.07-0.16) | 17.65(12.55-23.09) |
| People's Democratic Republic of Algeria | 70.47(59.29-84.38) | 2.42(2.08-2.86) | 0.26(0.17-0.39) | 27.26(20.6-34.66) |
| Islamic Republic of Iran | 74.72(65.11-85.41) | 2.69(2.36-3.03) | 0.45(0.36-0.54) | 34.84(28.52-41.15) |
| Kingdom of Bahrain | 59.15(48.62-70.72) | 2.01(1.67-2.41) | 0.17(0.12-0.23) | 20.17(15.19-25.92) |
| Federative Republic of Brazil | 25.7(21.83-30.28) | 1.06(0.91-1.23) | 0.15(0.14-0.16) | 11.33(9.19-13.64) |
| Republic of Paraguay | 19.15(15.83-23.11) | 0.8(0.66-0.95) | 0.14(0.1-0.2) | 9.2(7.2-11.99) |
| Palestine | 64.41(52.94-77.08) | 2.18(1.8-2.6) | 0.32(0.23-0.44) | 27.24(20.98-34.31) |
| Syrian Arab Republic | 61.92(51.53-73.98) | 2.1(1.75-2.5) | 0.13(0.08-0.22) | 20.18(14.64-27.24) |
| Republic of Turkey | 61.97(59.01-64.57) | 2.11(2.01-2.23) | 0.22(0.16-0.3) | 23.32(18.44-28.92) |
| Republic of Tunisia | 77.64(63.95-93.11) | 2.62(2.19-3.13) | 0.28(0.16-0.45) | 29.76(21.65-39.3) |
| United Arab Emirates | 37.37(32.61-43.33) | 1.38(1.23-1.58) | 0.1(0.06-0.14) | 12.28(9.25-15.76) |
| Republic of Yemen | 37.04(30.09-44.73) | 1.33(1.1-1.59) | 0.12(0.07-0.21) | 13.92(10.05-19.09) |
| Kingdom of Saudi Arabia | 48.81(39.83-60.3) | 1.67(1.37-2.03) | 0.12(0.07-0.19) | 16.97(12.32-22.58) |
| Sultanate of Oman | 63.49(52.6-77.29) | 2.18(1.83-2.63) | 0.18(0.08-0.39) | 22.38(14.74-31.5) |
| State of Qatar | 118.76(106.39-133.01) | 3.82(3.39-4.31) | 0.08(0.06-0.13) | 31.73(22.34-42.25) |
| Kingdom of Morocco | 68.16(56.2-82.43) | 2.37(2-2.83) | 0.28(0.16-0.45) | 27.41(20.14-38.18) |
| State of Libya | 66.96(55.74-81.29) | 2.31(1.98-2.79) | 0.56(0.36-0.88) | 37.61(27.86-50.62) |
| Democratic Republic of the Congo | 5.03(3.98-6.28) | 0.27(0.22-0.32) | 0.02(0.01-0.04) | 2.24(1.59-3.06) |
| Kingdom of Bhutan | 11.46(9.56-13.63) | 0.54(0.46-0.64) | 0.02(0.01-0.04) | 3.94(2.81-5.32) |
| Republic of the Congo | 5.23(4.21-6.49) | 0.28(0.23-0.34) | 0.04(0.02-0.06) | 2.77(1.95-3.83) |
| Central African Republic | 5.1(4.08-6.29) | 0.3(0.25-0.36) | 0.02(0.01-0.04) | 2.13(1.48-3.06) |
| Republic of Angola | 6.34(5.18-7.87) | 0.33(0.27-0.4) | 0.03(0.01-0.06) | 2.94(2.04-4.19) |
| Islamic Republic of Pakistan | 12.31(10.3-14.61) | 0.6(0.51-0.7) | 0.03(0.01-0.05) | 4.4(3.3-5.77) |
| Republic of India | 10.23(8.41-12.19) | 0.49(0.41-0.58) | 0.02(0.02-0.03) | 3.68(2.76-4.81) |
| Federal Democratic Republic of Nepal | 11.27(9.32-13.39) | 0.54(0.45-0.63) | 0.02(0.01-0.03) | 3.82(2.73-5.34) |
| People's Republic of Bangladesh | 9.71(7.92-11.74) | 0.45(0.37-0.54) | 0.02(0.01-0.04) | 3.45(2.45-4.77) |
| Islamic Republic of Afghanistan | 63.96(53.87-76.18) | 2.36(2.03-2.77) | 0.3(0.15-0.55) | 26.85(19.14-38.41) |
| Republic of Mozambique | 7.25(5.89-8.75) | 0.38(0.32-0.46) | 0.03(0-0.06) | 3.02(1.89-4.48) |
| Republic of Malawi | 6.47(5.21-7.93) | 0.34(0.28-0.41) | 0.03(0.01-0.05) | 2.8(1.73-4.11) |
| Republic of Madagascar | 7.51(6.14-9.1) | 0.38(0.31-0.46) | 0.03(0.01-0.05) | 3.15(2.1-4.43) |
| Republic of Mauritius | 3.34(2.63-4.25) | 0.22(0.18-0.26) | 0.03(0.03-0.03) | 2.19(1.82-2.61) |
| Federal Democratic Republic of Ethiopia | 5.95(4.76-7.3) | 0.3(0.25-0.36) | 0.02(0.01-0.04) | 2.51(1.66-3.38) |
| Republic of Kenya | 5.46(4.4-6.73) | 0.28(0.23-0.33) | 0.03(0.02-0.04) | 2.48(1.88-3.16) |
| Union of the Comoros | 6.77(5.52-8.17) | 0.34(0.28-0.41) | 0.04(0.01-0.06) | 3.15(2.18-4.45) |
| Republic of Djibouti | 6.79(5.5-8.23) | 0.34(0.28-0.41) | 0.03(0.01-0.06) | 3(1.98-4.32) |
| State of Eritrea | 7.27(5.91-8.81) | 0.39(0.33-0.46) | 0.04(0.01-0.06) | 3.26(2.19-4.68) |
| Republic of Burundi | 5.02(3.98-6.27) | 0.28(0.22-0.33) | 0.02(0-0.03) | 1.99(1.31-2.81) |
| Republic of Equatorial Guinea | 5.3(4.3-6.54) | 0.27(0.22-0.33) | 0.04(0.02-0.07) | 2.78(1.98-3.91) |
| Gabonese Republic | 5.22(4.16-6.36) | 0.27(0.22-0.32) | 0.04(0.02-0.06) | 2.67(1.9-3.58) |
| Republic of Zimbabwe | 6.79(5.51-8.21) | 0.36(0.29-0.42) | 0(0-0) | 1.91(1.27-2.61) |
| Republic of Botswana | 7.55(6.14-9.25) | 0.37(0.31-0.44) | 0.01(0.01-0.02) | 2.6(1.9-3.43) |
| Kingdom of Eswatini | 7.96(6.49-9.64) | 0.4(0.33-0.47) | 0.02(0.01-0.03) | 2.9(2.12-3.91) |
| Republic of Namibia | 7.4(6.03-9.11) | 0.36(0.29-0.43) | 0.02(0.01-0.03) | 2.67(1.9-3.62) |
| Republic of South Africa | 9.44(7.82-11.32) | 0.43(0.36-0.51) | 0.15(0.11-0.18) | 7.4(6.02-9.12) |
| Republic of Zambia | 6.91(5.59-8.45) | 0.37(0.3-0.44) | 0.04(0.02-0.06) | 3.21(2.32-4.46) |
| Kingdom of Lesotho | 8.49(6.96-10.14) | 0.44(0.37-0.52) | 0.02(0.01-0.03) | 3.01(2.21-4.08) |
| Republic of Seychelles | 4.72(3.79-5.76) | 0.33(0.27-0.39) | 0.02(0.01-0.03) | 2.16(1.58-2.88) |
| United Republic of Tanzania | 5.75(4.61-7.13) | 0.3(0.25-0.36) | 0.03(0.01-0.05) | 2.51(1.73-3.47) |
| Republic of Uganda | 4.83(3.79-6.02) | 0.25(0.2-0.31) | 0.02(0.01-0.03) | 2.01(1.35-2.84) |
| Republic of Rwanda | 5.1(4.04-6.36) | 0.27(0.22-0.32) | 0.02(0.01-0.04) | 2.25(1.57-3.1) |
| Federal Republic of Somalia | 5.15(4.16-6.32) | 0.29(0.24-0.35) | 0.01(0-0.02) | 1.86(1.21-2.66) |
| Republic of Mali | 11.2(9.39-13.18) | 0.59(0.51-0.68) | 0.14(0.03-0.34) | 12.48(5.19-25.9) |
| Republic of Liberia | 8.39(6.92-10.06) | 0.44(0.37-0.52) | 0.13(0.04-0.33) | 11.44(4.94-25.54) |
| Republic of Guinea-Bissau | 10.06(8.31-11.85) | 0.56(0.48-0.65) | 0.15(0.05-0.38) | 13.04(6.22-28.09) |
| Republic of Ghana | 33.36(28.96-38.32) | 1.73(1.58-1.89) | 0.17(0.05-0.42) | 20.01(11.76-37.86) |
| Republic of Guinea | 9.23(7.62-11.12) | 0.5(0.42-0.59) | 0.11(0.03-0.3) | 10.37(4.84-22.72) |
| Republic of Côte d'Ivoire | 8.29(6.79-9.83) | 0.44(0.37-0.51) | 0.12(0.04-0.34) | 10.71(4.75-25.31) |
| Republic of the Gambia | 10.58(8.85-12.5) | 0.54(0.46-0.63) | 0.15(0.04-0.38) | 13.39(5.46-28.85) |
| Republic of Chad | 9.83(8.03-11.89) | 0.53(0.45-0.62) | 0.08(0.02-0.23) | 8.36(3.83-17.85) |
| Republic of Cameroon | 8.74(7.14-10.36) | 0.47(0.39-0.55) | 0.14(0.04-0.35) | 11.64(5.09-26.3) |
| Republic of Cabo Verde | 11.31(9.47-13.45) | 0.51(0.43-0.6) | 0.11(0.03-0.3) | 10.66(4.76-23.85) |
| Burkina Faso | 9.31(7.74-11.18) | 0.49(0.42-0.58) | 0.1(0.02-0.29) | 9.32(4.06-21.65) |
| Republic of Benin | 9.11(7.54-10.85) | 0.47(0.39-0.55) | 0.11(0.03-0.27) | 9.72(4.71-20.39) |
| Islamic Republic of Mauritania | 13.09(10.98-15.43) | 0.64(0.56-0.75) | 0.16(0.05-0.38) | 14.16(6.7-29.69) |
| Federal Republic of Nigeria | 9.39(7.72-11.21) | 0.47(0.39-0.55) | 0.11(0.06-0.19) | 9.85(6.23-15.58) |
| Republic of Senegal | 10.65(8.74-12.5) | 0.54(0.46-0.63) | 0.14(0.04-0.39) | 12.72(5.68-29.61) |
| Democratic Republic of Sao Tome and Principe | 7.43(6.11-8.97) | 0.37(0.31-0.44) | 0.03(0.01-0.07) | 4.14(2.49-7.3) |
| Republic of the Niger | 10.35(8.55-12.3) | 0.55(0.46-0.64) | 0.07(0.01-0.2) | 7.87(3.34-16.79) |
| Republic of Sierra Leone | 8.6(6.99-10.33) | 0.46(0.38-0.54) | 0.11(0.03-0.27) | 9.72(4.48-20.7) |
| Togolese Republic | 9.02(7.42-10.62) | 0.47(0.39-0.55) | 0.13(0.04-0.31) | 11.6(5.16-24.35) |
| American Samoa | 2.44(1.86-3.12) | 0.18(0.14-0.22) | 0(0-0) | 0.69(0.45-0.97) |
| Bermuda | 24.18(20.26-28.64) | 0.95(0.82-1.12) | 0.26(0.21-0.33) | 15.88(13.06-19.24) |
| Cook Islands | 3.01(2.37-3.78) | 0.19(0.15-0.23) | 0(0-0) | 0.85(0.56-1.18) |
| Greenland | 61.11(49.95-73.39) | 1.7(1.41-2.02) | 0(0-0) | 15.42(10.17-21.45) |
| Guam | 2.46(1.91-3.14) | 0.16(0.13-0.2) | 0(0-0) | 0.7(0.46-0.98) |
| Principality of Monaco | 82.64(69.9-95.95) | 2.92(2.54-3.37) | 0.61(0.36-1) | 41.07(29.94-56.46) |
| Republic of Nauru | 1.79(1.36-2.32) | 0.16(0.13-0.2) | 0(0-0) | 0.51(0.33-0.74) |
| Republic of Niue | 2.95(2.31-3.74) | 0.21(0.17-0.26) | 0(0-0) | 0.85(0.56-1.17) |
| Northern Mariana Islands | 2.77(2.18-3.51) | 0.19(0.15-0.23) | 0(0-0) | 0.78(0.52-1.11) |
| Republic of Palau | 2.18(1.68-2.78) | 0.17(0.13-0.21) | 0(0-0) | 0.62(0.41-0.87) |
| Puerto Rico | 18.4(15.22-22.2) | 0.77(0.65-0.9) | 0.31(0.24-0.38) | 16.41(13.2-19.89) |
| Saint Kitts and Nevis | 21.03(17.86-24.85) | 0.97(0.85-1.11) | 0.37(0.29-0.47) | 18.69(15.25-22.93) |
| Tokelau | 2.35(1.82-2.98) | 0.18(0.15-0.22) | 0(0-0) | 0.68(0.44-0.95) |
| Tuvalu | 2.08(1.61-2.64) | 0.17(0.14-0.21) | 0(0-0) | 0.59(0.39-0.82) |
| Republic of San Marino | 52.6(43.47-63.85) | 1.79(1.48-2.14) | 0(0-0) | 13.5(8.85-19.29) |
| United States Virgin Islands | 17.59(14.56-20.84) | 0.76(0.65-0.89) | 0.24(0.14-0.39) | 14.63(9.88-21.64) |
| Republic of South Sudan | 5.47(4.34-6.73) | 0.29(0.23-0.35) | 0.02(0-0.04) | 2.19(1.43-3.21) |
| Republic of Sudan | 38.9(32.21-47.3) | 1.38(1.15-1.66) | 0.15(0.09-0.26) | 15.92(11.46-21.53) |

MS, Multiple sclerosis; SDI, socio-demographic index.

Supplementary Table 3: Disease burden of MS in women of childbearing age by age group in 2021

| Metric | age | Prevalence..95.UI. | Incidence..95.UI. | Deaths..95.UI. | DALYs..95.UI. |
| --- | --- | --- | --- | --- | --- |
| Number | 15-19 years | 11507.3(7798.08-15676.15) | 2755.94(1779.19-3931.4) | 17.31(14.05-20.98) | 4497.92(3118.56-6383.94) |
|  | 20-24 years | 30361.8(22386.63-40651.83) | 4896.26(3360.59-6741.32) | 388.1(252.48-545.85) | 34611.93(24396.56-45775.23) |
|  | 25-29 years | 60308.41(46458.27-75872.75) | 6821.17(4734.1-9070.61) | 87.88(79.02-97.89) | 21616.94(15584.03-28479.79) |
|  | 30-34 years | 99237.34(80045.32-118260.15) | 7563.59(5212.63-10281.43) | 188.43(175.59-205.11) | 37006.26(28347.49-47222.47) |
|  | 35-39 years | 128282.38(108821.22-150288.5) | 5790.4(3730.31-7965.84) | 312.19(288.42-340.27) | 49955.2(39965.67-61121.5) |
|  | 40-44 years | 138239.57(122219.39-158738.69) | 3679.07(2235.12-5463) | 464.88(431.76-507.25) | 57904.44(46799.6-69141.43) |
|  | 45-49 years | 138774.44(123534.35-156750.91) | 2433.42(1615.57-3416.69) | 653.2(611.76-696.65) | 63580.91(53117.72-74776.63) |
|  |  |  |  |  |  |
| Rate | 15-19 years | 3.79(2.57-5.16) | 0.91(0.59-1.29) | 0.01(0-0.01) | 1.48(1.03-2.1) |
|  | 20-24 years | 10.34(7.62-13.84) | 1.67(1.14-2.29) | 0.13(0.09-0.19) | 11.78(8.31-15.58) |
|  | 25-29 years | 20.73(15.97-26.07) | 2.34(1.63-3.12) | 0.03(0.03-0.03) | 7.43(5.36-9.79) |
|  | 30-34 years | 33.2(26.78-39.56) | 2.53(1.74-3.44) | 0.06(0.06-0.07) | 12.38(9.48-15.8) |
|  | 35-39 years | 46.18(39.17-54.1) | 2.08(1.34-2.87) | 0.11(0.1-0.12) | 17.98(14.39-22) |
|  | 40-44 years | 55.72(49.26-63.98) | 1.48(0.9-2.2) | 0.19(0.17-0.2) | 23.34(18.86-27.87) |
|  | 45-49 years | 58.89(52.42-66.52) | 1.03(0.69-1.45) | 0.28(0.26-0.3) | 26.98(22.54-31.73) |
| *MS, Multiple sclerosis.* | | | | | |

Supplementary Table 4: The Estimated Annual Percentage Change (EAPC) in ASIR, ASPR, ASMR, and ASDR for MS in Women of Childbearing Age from 1990 to 2021, Globally, by Country, and by Region

| location | Prevalence (95%CI) | Incidence (95%CI) | Deaths (95%CI) | DALYs (95%CI) |
| --- | --- | --- | --- | --- |
| Global | 0.1 (0.06-0.14) | 0.04 (-0.02-0.1) | -0.35 (-0.46--0.24) | -0.3 (-0.37--0.23) |
| Low SDI | 0.57 (0.52-0.62) | 0.38 (0.35-0.42) | 2.05 (1.98-2.12) | 1.2 (1.13-1.27) |
| Low-middle SDI | 1.13 (1.08-1.18) | 0.84 (0.8-0.89) | 2.26 (2.15-2.37) | 1.54 (1.52-1.56) |
| Middle SDI | 1.33 (1.28-1.38) | 0.89 (0.85-0.93) | 1.57 (1.32-1.83) | 1.52 (1.43-1.62) |
| High-middle SDI | 0.19 (0.16-0.22) | 0 (-0.05-0.06) | -2.03 (-2.21--1.85) | -1.27 (-1.39--1.14) |
| High SDI | 0.69 (0.63-0.75) | 0.61 (0.57-0.64) | 0.43 (0.29-0.57) | 0.32 (0.22-0.42) |
| 21 Regions |  |  |  |  |
| Andean Latin America | 1.34 (1.28-1.41) | 1.06 (1-1.11) | 2.89 (2.48-3.29) | 1.98 (1.76-2.2) |
| Australasia | 1.45 (1.14-1.75) | 1.24 (0.99-1.49) | 0.14 (-0.06-0.35) | 0.69 (0.45-0.93) |
| Caribbean | 0.76 (0.68-0.84) | 0.43 (0.39-0.48) | 0.71 (0.55-0.87) | 0.59 (0.48-0.69) |
| Central Asia | 0.08 (0.04-0.11) | -0.38 (-0.42--0.33) | -0.64 (-1.3-0.02) | -0.4 (-0.58--0.21) |
| Central Europe | 0.42 (0.34-0.5) | -0.14 (-0.18--0.1) | -1.41 (-1.47--1.36) | -1.06 (-1.09--1.03) |
| Central Latin America | 1.46 (1.36-1.56) | 1.16 (1.07-1.24) | 2.13 (1.75-2.5) | 1.87 (1.59-2.15) |
| Central Sub-Saharan Africa | 0.24 (0.18-0.3) | 0.07 (0.02-0.11) | 1.56 (1.41-1.71) | 0.67 (0.57-0.76) |
| East Asia | 1.04 (0.87-1.22) | -0.69 (-0.83--0.56) | -0.21 (-0.8-0.39) | 0.58 (0.4-0.75) |
| Eastern Europe | 0.67 (0.6-0.75) | -0.21 (-0.26--0.16) | -2.68 (-3.07--2.29) | -1.89 (-2.2--1.58) |
| Eastern Sub-Saharan Africa | 0.09 (0.03-0.16) | -0.1 (-0.17--0.04) | 1.44 (1.36-1.52) | 0.46 (0.39-0.53) |
| High-income Asia Pacific | 0.49 (0.43-0.56) | 0.27 (0.22-0.32) | -0.25 (-0.58-0.08) | 0.24 (0.1-0.38) |
| High-income North America | 0.52 (0.44-0.6) | 0.42 (0.34-0.5) | 1.09 (0.74-1.44) | 0.46 (0.28-0.64) |
| North Africa and Middle East | 1.17 (1.11-1.22) | 0.91 (0.84-0.97) | 2.39 (2.2-2.57) | 1.47 (1.4-1.53) |
| Oceania | -0.04 (-0.1-0.01) | -0.28 (-0.33--0.23) | 0.94 (0.79-1.08) | -0.04 (-0.09-0.02) |
| South Asia | 0.58 (0.55-0.62) | 0.41 (0.37-0.44) | 2.05 (1.92-2.19) | 0.84 (0.79-0.88) |
| Southeast Asia | 0.23 (0.18-0.28) | -0.17 (-0.21--0.12) | 1.65 (1.43-1.88) | 0.82 (0.75-0.9) |
| Southern Latin America | 0.15 (0.13-0.17) | 0.07 (0.05-0.09) | -1.69 (-1.96--1.42) | -0.87 (-1.01--0.74) |
| Southern Sub-Saharan Africa | 0.19 (0.12-0.26) | 0.08 (0.04-0.13) | 0.68 (0.52-0.83) | 0.32 (0.24-0.41) |
| Tropical Latin America | 0.69 (0.58-0.8) | 0.37 (0.29-0.45) | -0.06 (-0.63-0.51) | 0.4 (0.16-0.64) |
| Western Europe | 1.08 (1.01-1.14) | 0.94 (0.91-0.97) | 0.81 (0.71-0.92) | 0.77 (0.7-0.83) |
| Western Sub-Saharan Africa | 0.86 (0.82-0.91) | 0.65 (0.62-0.67) | 1.8 (1.66-1.95) | 1.48 (1.4-1.57) |
| 204 Countries |  |  |  |  |
| People's Republic of China | 0.95 (0.76-1.14) | -0.77 (-0.9--0.63) | -0.16 (-0.74-0.43) | 0.54 (0.37-0.71) |
| Democratic People's Republic of Korea | 0.58 (0.53-0.63) | -0.37 (-0.42--0.31) | 1.24 (1.13-1.35) | 0.79 (0.77-0.8) |
| Taiwan (Province of China) | 3.69 (3.26-4.11) | 1.75 (1.46-2.03) | -0.62 (-2.51-1.27) | 1.5 (0.61-2.38) |
| Kingdom of Cambodia | 0.38 (0.32-0.44) | -0.15 (-0.19--0.11) | 2.51 (2.29-2.73) | 1.09 (1.06-1.13) |
| Lao People's Democratic Republic | 0.28 (0.21-0.34) | -0.3 (-0.36--0.25) | 2.4 (2.11-2.69) | 1 (0.94-1.06) |
| Malaysia | 0.33 (0.3-0.37) | -0.14 (-0.18--0.1) | 1.39 (1.09-1.69) | 1.02 (0.86-1.18) |
| Republic of Indonesia | 0.18 (0.12-0.25) | -0.1 (-0.15--0.04) | 2.22 (1.86-2.59) | 0.92 (0.82-1.01) |
| Republic of Maldives | 0.24 (0.2-0.28) | -0.44 (-0.5--0.38) | 1.39 (0.9-1.88) | 0.65 (0.49-0.81) |
| Republic of the Union of Myanmar | 0.23 (0.16-0.29) | -0.31 (-0.35--0.26) | 1.61 (1.28-1.94) | 0.69 (0.59-0.79) |
| Republic of the Philippines | 0.04 (-0.04-0.12) | -0.25 (-0.31--0.19) | 0.58 (0.42-0.75) | 0.34 (0.21-0.47) |
| Democratic Socialist Republic of Sri Lanka | 0.16 (0.13-0.19) | -0.15 (-0.17--0.13) | 0.61 (0.39-0.84) | 0.24 (0.19-0.29) |
| Kingdom of Thailand | 0.23 (0.18-0.27) | -0.2 (-0.25--0.16) | 1.58 (1.38-1.78) | 0.59 (0.53-0.65) |
| Democratic Republic of Timor-Leste | 0.35 (0.29-0.41) | -0.11 (-0.17--0.05) | 2.69 (2.49-2.89) | 1.02 (0.94-1.1) |
| Socialist Republic of Viet Nam | 0.39 (0.35-0.42) | -0.06 (-0.09--0.03) | 2.6 (2.33-2.87) | 1.11 (1.06-1.16) |
| Republic of Fiji | 0.1 (0.04-0.16) | -0.18 (-0.22--0.14) | 0.62 (0.48-0.75) | 0.1 (0.04-0.16) |
| Republic of Kiribati | 0.35 (0.32-0.38) | 0.03 (-0.02-0.08) | 0.38 (0.12-0.65) | 0.35 (0.32-0.38) |
| Republic of the Marshall Islands | 0.25 (0.21-0.29) | -0.08 (-0.08--0.08) | 1.33 (1.27-1.39) | 0.26 (0.22-0.3) |
| Federated States of Micronesia | 0.23 (0.21-0.26) | -0.18 (-0.2--0.17) | 1.01 (0.93-1.08) | 0.24 (0.21-0.27) |
| Independent State of Samoa | 0.2 (0.16-0.23) | -0.13 (-0.15--0.11) | 1.38 (1.26-1.51) | 0.2 (0.17-0.24) |
| Independent State of Papua New Guinea | 0.03 (-0.05-0.11) | -0.26 (-0.33--0.19) | 1.24 (1.1-1.39) | 0.04 (-0.04-0.12) |
| Solomon Islands | 0.29 (0.28-0.31) | 0 (-0.02-0.02) | 1.37 (1.21-1.54) | 0.29 (0.28-0.31) |
| Kingdom of Tonga | 0.12 (0.06-0.17) | -0.18 (-0.23--0.14) | 1.57 (1.49-1.66) | 0.12 (0.07-0.18) |
| Republic of Vanuatu | 0.15 (0.11-0.19) | -0.13 (-0.16--0.1) | 1.23 (1.11-1.35) | 0.15 (0.11-0.2) |
| Republic of Armenia | 1.52 (1.43-1.6) | 1.34 (1.24-1.43) | 3.27 (1.84-4.7) | 1.71 (1.31-2.11) |
| Republic of Azerbaijan | 0.37 (0.24-0.51) | 0.07 (-0.04-0.18) | -0.47 (-1.02-0.09) | 0.12 (-0.07-0.3) |
| Georgia | 1.13 (1.09-1.17) | 1.01 (0.96-1.07) | 3.61 (1.87-5.34) | 1.64 (1.27-2.02) |
| Republic of Kazakhstan | 0.49 (0.46-0.52) | 0.24 (0.22-0.26) | -2.71 (-2.94--2.48) | -0.92 (-1.02--0.83) |
| Kyrgyz Republic | 0.31 (0.17-0.45) | -0.03 (-0.15-0.08) | 1.81 (-0.02-3.65) | 0.58 (0.02-1.15) |
| Mongolia | 0.25 (0.06-0.43) | -0.35 (-0.54--0.17) | 0.83 (0.67-1) | 0.54 (0.42-0.67) |
| Republic of Tajikistan | -0.08 (-0.22-0.05) | -0.35 (-0.47--0.23) | 0.49 (-0.08-1.07) | -0.01 (-0.23-0.22) |
| Turkmenistan | 0.33 (0.29-0.38) | 0.09 (0.05-0.12) | -1.4 (-1.84--0.97) | -0.4 (-0.61--0.19) |
| Republic of Uzbekistan | -0.71 (-0.84--0.58) | -1.02 (-1.14--0.9) | 2.45 (1.34-3.57) | 0.07 (-0.23-0.36) |
| Republic of Albania | -0.13 (-0.24--0.01) | -0.79 (-0.87--0.71) | -0.9 (-1.11--0.68) | -0.6 (-0.72--0.47) |
| Bosnia and Herzegovina | 0.19 (0.17-0.21) | -0.19 (-0.23--0.15) | -1.41 (-1.59--1.24) | -0.96 (-1.08--0.83) |
| Republic of Bulgaria | 0.3 (0.19-0.41) | -0.03 (-0.12-0.05) | -0.52 (-0.8--0.25) | -0.16 (-0.34-0.02) |
| Republic of Croatia | 1.65 (1.53-1.78) | 1.23 (1.12-1.34) | -1.03 (-1.16--0.9) | -0.49 (-0.58--0.4) |
| Czech Republic | -0.12 (-0.2--0.04) | -0.63 (-0.71--0.56) | -1.82 (-2--1.63) | -1.6 (-1.73--1.47) |
| Hungary | -0.97 (-1.33--0.61) | -1.18 (-1.48--0.89) | -1.37 (-1.58--1.17) | -1.43 (-1.51--1.35) |
| North Macedonia | 1.17 (1.07-1.28) | 0.8 (0.72-0.88) | -0.34 (-0.48--0.21) | -0.01 (-0.12-0.1) |
| Montenegro | 0.49 (0.44-0.54) | 0.2 (0.16-0.24) | 0.31 (0.17-0.44) | 0.21 (0.09-0.33) |
| Republic of Poland | 0.47 (0.4-0.55) | -0.28 (-0.3--0.26) | -1.88 (-1.99--1.76) | -1.39 (-1.46--1.31) |
| Romania | -0.14 (-0.21--0.08) | -0.67 (-0.74--0.6) | -2.43 (-2.62--2.24) | -2.14 (-2.31--1.97) |
| Republic of Serbia | 1.03 (0.95-1.11) | 0.62 (0.56-0.67) | -0.2 (-0.33--0.07) | 0.08 (-0.04-0.19) |
| Slovak Republic | 0.73 (0.72-0.75) | 0.44 (0.43-0.46) | -0.32 (-0.4--0.25) | -0.13 (-0.19--0.07) |
| Republic of Slovenia | 0.3 (0.27-0.33) | -0.07 (-0.1--0.04) | -2.23 (-2.41--2.06) | -1.88 (-2.02--1.75) |
| Republic of Belarus | 1.15 (0.95-1.34) | 0.27 (0.15-0.39) | -1.81 (-2.38--1.24) | -1.2 (-1.62--0.78) |
| Republic of Estonia | 0.65 (0.54-0.76) | -0.21 (-0.24--0.18) | -4.54 (-4.99--4.1) | -3.88 (-4.28--3.47) |
| Republic of Latvia | 0.86 (0.72-0.99) | 0 (-0.04-0.04) | -3.45 (-3.85--3.04) | -3.06 (-3.43--2.69) |
| Republic of Lithuania | 0.84 (0.73-0.94) | 0.02 (-0.01-0.05) | -2.97 (-3.27--2.66) | -2.63 (-2.91--2.34) |
| Republic of Moldova | 1.24 (0.98-1.5) | -0.31 (-0.48--0.13) | -3.24 (-3.58--2.91) | -2.13 (-2.38--1.88) |
| Russian Federation | 0.67 (0.54-0.8) | -0.31 (-0.4--0.21) | -2.58 (-2.97--2.19) | -1.76 (-2.07--1.45) |
| Ukraine | 0.57 (0.46-0.68) | 0 (-0.04-0.05) | -2.78 (-3.32--2.25) | -2.04 (-2.44--1.64) |
| Brunei Darussalam | 0.28 (0.26-0.3) | 0.1 (0.09-0.11) | 0.57 (0.47-0.68) | 0.38 (0.34-0.43) |
| Japan | 0.59 (0.51-0.68) | 0.42 (0.35-0.49) | 0.25 (-0.1-0.59) | 0.47 (0.31-0.63) |
| Republic of Korea | 0.35 (0.34-0.36) | 0.05 (0.02-0.08) | -1.51 (-1.79--1.23) | -0.16 (-0.22--0.09) |
| Republic of Singapore | 0.37 (0.33-0.42) | 0.17 (0.14-0.2) | -1.69 (-2.26--1.12) | -0.26 (-0.45--0.07) |
| Australia | 1.67 (1.32-2.03) | 1.46 (1.16-1.76) | 0.27 (0.11-0.43) | 0.9 (0.65-1.15) |
| Principality of Andorra | 1.07 (0.98-1.17) | 0.99 (0.91-1.07) | 0.38 (0.17-0.59) | 0.57 (0.44-0.71) |
| New Zealand | -0.14 (-0.17--0.11) | -0.19 (-0.22--0.15) | -0.32 (-0.71-0.07) | -0.36 (-0.61--0.11) |
| Kingdom of Belgium | 1.09 (1.02-1.15) | 0.95 (0.89-1.01) | 0.68 (0.54-0.83) | 0.75 (0.64-0.85) |
| Republic of Finland | 0.96 (0.86-1.06) | 0.85 (0.77-0.93) | 0.79 (0.69-0.89) | 0.74 (0.69-0.8) |
| Kingdom of Denmark | 0.8 (0.73-0.87) | 0.58 (0.53-0.64) | -0.31 (-0.45--0.16) | -0.01 (-0.11-0.08) |
| French Republic | 1.39 (1.24-1.54) | 1.14 (1.03-1.25) | 0.41 (0.21-0.6) | 0.79 (0.63-0.95) |
| Republic of Austria | 1.35 (1.26-1.45) | 1.26 (1.17-1.35) | 1.36 (1.14-1.58) | 1.16 (1.05-1.27) |
| Hellenic Republic | 1.56 (1.51-1.62) | 1.37 (1.32-1.42) | 2.12 (2.05-2.2) | 1.87 (1.81-1.94) |
| Republic of Cyprus | 1.79 (1.62-1.97) | 1.64 (1.48-1.8) | -0.02 (-0.13-0.09) | 0.87 (0.76-0.97) |
| Federal Republic of Germany | 0.95 (0.87-1.02) | 0.81 (0.75-0.87) | 0.84 (0.71-0.98) | 0.63 (0.56-0.69) |
| State of Israel | 0.7 (0.67-0.73) | 0.55 (0.53-0.56) | 0.29 (0.17-0.41) | 0.45 (0.39-0.51) |
| Grand Duchy of Luxembourg | 0.85 (0.78-0.91) | 0.68 (0.63-0.73) | 0.31 (0.09-0.54) | 0.45 (0.33-0.57) |
| Republic of Iceland | 0.51 (0.46-0.56) | 0.33 (0.28-0.38) | 0.54 (0.42-0.66) | 0.46 (0.39-0.53) |
| Kingdom of Norway | 1.63 (1.57-1.68) | 1.42 (1.38-1.46) | 0 (-0.33-0.33) | 0.59 (0.41-0.77) |
| Kingdom of Spain | 1.01 (0.88-1.15) | 0.81 (0.69-0.92) | 0.72 (0.6-0.84) | 0.78 (0.67-0.89) |
| Republic of Italy | 1.1 (1.02-1.18) | 0.94 (0.87-1.02) | 1.36 (1.11-1.6) | 1.01 (0.91-1.12) |
| Swiss Confederation | 0.42 (0.37-0.46) | 0.29 (0.25-0.33) | -0.24 (-0.39--0.08) | -0.08 (-0.18-0.02) |
| Ireland | 0.76 (0.58-0.94) | 0.7 (0.53-0.87) | 0.08 (-0.05-0.2) | 0.4 (0.27-0.53) |
| Kingdom of Sweden | 0.8 (0.75-0.86) | 0.79 (0.74-0.84) | 1.15 (0.97-1.33) | 0.77 (0.7-0.85) |
| Portuguese Republic | 0.24 (0.23-0.25) | 0.06 (0.04-0.08) | 0.29 (0.18-0.41) | 0.19 (0.13-0.25) |
| Kingdom of the Netherlands | 0.79 (0.72-0.86) | 0.67 (0.61-0.74) | 0.39 (0.24-0.54) | 0.48 (0.38-0.58) |
| Republic of Malta | 1.32 (1.15-1.48) | 0.93 (0.78-1.08) | 0.91 (0.77-1.04) | 1.15 (1.04-1.26) |
| United Kingdom of Great Britain and Northern Ireland | 1.4 (1.32-1.47) | 1.12 (1.06-1.18) | 1.27 (1.1-1.44) | 1.11 (1.01-1.22) |
| Eastern Republic of Uruguay | 0.35 (0.33-0.38) | 0.27 (0.25-0.29) | -1.16 (-1.44--0.87) | -0.69 (-0.88--0.49) |
| United States of America | 0.5 (0.4-0.61) | 0.42 (0.32-0.51) | 1.17 (0.81-1.53) | 0.47 (0.27-0.66) |
| Argentine Republic | 0.09 (0.06-0.11) | -0.02 (-0.03-0) | -1.47 (-1.76--1.19) | -0.83 (-0.99--0.68) |
| Antigua and Barbuda | 1.9 (1.69-2.12) | 1.7 (1.51-1.9) | 1.43 (1.17-1.69) | 1.42 (1.24-1.61) |
| Commonwealth of the Bahamas | 0.9 (0.84-0.97) | 0.68 (0.63-0.72) | 1.74 (1.57-1.92) | 1.47 (1.34-1.6) |
| Barbados | 1.74 (1.54-1.94) | 1.51 (1.34-1.67) | 1.94 (1.73-2.16) | 1.71 (1.53-1.88) |
| Canada | 0.58 (0.52-0.64) | 0.46 (0.39-0.53) | 0.43 (0.15-0.71) | 0.36 (0.25-0.47) |
| Republic of Chile | 0.32 (0.27-0.37) | 0.17 (0.11-0.23) | -2.22 (-2.43--2.01) | -0.75 (-0.83--0.67) |
| Republic of Cuba | 1.12 (1.01-1.23) | 0.93 (0.84-1.02) | 0.7 (0.52-0.88) | 0.56 (0.43-0.69) |
| Commonwealth of Dominica | 0.47 (0.44-0.49) | 0.25 (0.21-0.29) | 1.82 (1.65-1.99) | 1.23 (1.14-1.32) |
| Republic of Haiti | 0.51 (0.47-0.56) | 0.24 (0.22-0.26) | 1.34 (1.19-1.49) | 1.04 (0.95-1.14) |
| Republic of Suriname | 0.52 (0.5-0.55) | 0.24 (0.2-0.28) | 1.3 (1.14-1.45) | 0.97 (0.89-1.06) |
| Saint Lucia | 0.65 (0.63-0.67) | 0.38 (0.37-0.39) | 0.39 (0.08-0.7) | 0.55 (0.33-0.77) |
| Republic of Trinidad and Tobago | 1.01 (0.97-1.05) | 0.68 (0.66-0.7) | 1.22 (1.06-1.38) | 1.3 (1.18-1.42) |
| Grenada | 0.9 (0.78-1.02) | 0.64 (0.55-0.72) | 1.15 (0.81-1.49) | 0.89 (0.65-1.14) |
| Republic of Guyana | 0.59 (0.56-0.63) | 0.3 (0.25-0.34) | 2.15 (1.95-2.35) | 1.6 (1.48-1.72) |
| Saint Vincent and the Grenadines | 0.89 (0.84-0.95) | 0.69 (0.65-0.74) | 1.13 (0.9-1.36) | 1.06 (0.9-1.22) |
| Jamaica | 0.5 (0.49-0.52) | 0.28 (0.25-0.31) | 2.44 (2.25-2.62) | 1.69 (1.56-1.83) |
| Belize | 0.54 (0.5-0.58) | 0.3 (0.25-0.35) | 2.47 (2.19-2.74) | 1.64 (1.5-1.77) |
| Dominican Republic | 0.56 (0.53-0.59) | 0.33 (0.29-0.38) | 1.71 (1.49-1.92) | 1.15 (1.05-1.25) |
| Republic of Peru | 1.41 (1.34-1.49) | 1.1 (1.04-1.15) | 3.12 (2.73-3.51) | 2.03 (1.85-2.2) |
| Republic of Honduras | 0.81 (0.78-0.84) | 0.51 (0.48-0.53) | 2.09 (1.89-2.29) | 0.81 (0.78-0.84) |
| Republic of Ecuador | 1.1 (1.05-1.14) | 0.86 (0.83-0.89) | 3.12 (2.38-3.87) | 2.16 (1.71-2.6) |
| Republic of Costa Rica | 1.35 (1.2-1.51) | 1.1 (0.96-1.24) | 1.77 (1.43-2.11) | 1.62 (1.37-1.88) |
| Bolivarian Republic of Venezuela | 1.22 (1.12-1.31) | 0.91 (0.84-0.99) | 2.23 (1.99-2.47) | 2.05 (1.86-2.25) |
| Republic of Guatemala | 1.34 (1.29-1.4) | 0.95 (0.93-0.98) | 1.38 (0.99-1.76) | 1.32 (1.06-1.57) |
| United Mexican States | 1.58 (1.46-1.71) | 1.31 (1.21-1.41) | 2.4 (1.96-2.85) | 2.07 (1.74-2.4) |
| Plurinational State of Bolivia | 1.42 (1.34-1.5) | 1.1 (1.03-1.16) | 2.08 (1.98-2.18) | 1.62 (1.54-1.7) |
| Republic of Nicaragua | 2.01 (1.85-2.17) | 1.67 (1.54-1.81) | 2.34 (1.87-2.8) | 1.99 (1.67-2.31) |
| Republic of Panama | 0.73 (0.68-0.77) | 0.44 (0.42-0.47) | 2.16 (1.73-2.58) | 1.72 (1.42-2.02) |
| Republic of El Salvador | 0.85 (0.83-0.87) | 0.48 (0.46-0.51) | 2.63 (2.35-2.91) | 1.77 (1.61-1.93) |
| Republic of Colombia | 1.24 (1.18-1.3) | 0.91 (0.86-0.95) | 1.15 (0.8-1.5) | 1.22 (0.96-1.48) |
| State of Kuwait | 1.8 (1.6-1.99) | 1.57 (1.41-1.73) | 19.68 (15.22-24.15) | 2.34 (1.96-2.73) |
| Lebanese Republic | 1.54 (1.51-1.58) | 1.35 (1.32-1.37) | 0.58 (0.44-0.72) | 1.32 (1.27-1.36) |
| Hashemite Kingdom of Jordan | 0.47 (0.43-0.51) | 0.38 (0.35-0.42) | -0.42 (-0.87-0.04) | 0.02 (-0.17-0.22) |
| Arab Republic of Egypt | 2.97 (2.82-3.12) | 2.59 (2.44-2.74) | 0.64 (0.47-0.82) | 2.66 (2.54-2.78) |
| Republic of Iraq | 1.18 (1.08-1.28) | 0.97 (0.87-1.07) | 1.39 (1.3-1.49) | 1.06 (1-1.12) |
| People's Democratic Republic of Algeria | 1.55 (1.53-1.57) | 1.35 (1.34-1.36) | 3.49 (3.09-3.88) | 2.11 (1.98-2.25) |
| Islamic Republic of Iran | 0.87 (0.66-1.07) | 0.66 (0.49-0.84) | 2 (1.79-2.21) | 1.31 (1.16-1.46) |
| Kingdom of Bahrain | 1.55 (1.53-1.58) | 1.38 (1.35-1.4) | 9.86 (7.87-11.84) | 2.63 (2.34-2.92) |
| Federative Republic of Brazil | 0.7 (0.59-0.8) | 0.38 (0.29-0.46) | -0.1 (-0.67-0.48) | 0.39 (0.15-0.64) |
| Republic of Paraguay | 0.36 (0.29-0.43) | 0.19 (0.11-0.27) | 2.04 (1.51-2.56) | 0.93 (0.74-1.12) |
| Palestine | 1.34 (1.3-1.37) | 1.17 (1.13-1.2) | 5.05 (4.37-5.74) | 2.55 (2.33-2.77) |
| Syrian Arab Republic | 1.26 (1.24-1.29) | 1.08 (1.05-1.1) | 1.28 (1.17-1.39) | 1.21 (1.18-1.23) |
| Republic of Turkey | 0.11 (0.08-0.13) | -0.07 (-0.08--0.06) | 0.98 (0.84-1.12) | 0.31 (0.25-0.37) |
| Republic of Tunisia | 1.49 (1.48-1.51) | 1.33 (1.31-1.34) | 3.43 (3.01-3.86) | 2.08 (1.93-2.22) |
| United Arab Emirates | 0.36 (0.26-0.46) | 0.25 (0.17-0.32) | 2.97 (2.64-3.29) | 0.62 (0.53-0.7) |
| Republic of Yemen | 1.28 (1.24-1.33) | 1.05 (1.01-1.09) | 3.76 (3.34-4.18) | 1.9 (1.8-1.99) |
| Kingdom of Saudi Arabia | 1.41 (1.36-1.46) | 1.16 (1.11-1.22) | 3.29 (2.99-3.6) | 1.76 (1.69-1.83) |
| Sultanate of Oman | 1.75 (1.71-1.8) | 1.56 (1.52-1.59) | 3.57 (2.94-4.2) | 2.16 (1.95-2.37) |
| State of Qatar | 1.08 (0.83-1.32) | 1.02 (0.81-1.24) | 10.7 (8.44-12.97) | 1.46 (1.2-1.73) |
| Kingdom of Morocco | 1.55 (1.52-1.58) | 1.36 (1.33-1.39) | 4.06 (3.72-4.41) | 2.28 (2.19-2.36) |
| State of Libya | 1.63 (1.53-1.72) | 1.46 (1.38-1.55) | 5.96 (5.76-6.16) | 3.41 (3.31-3.51) |
| Democratic Republic of the Congo | 0.17 (0.11-0.24) | 0.03 (-0.02-0.08) | 1.41 (1.16-1.65) | 0.54 (0.41-0.67) |
| Kingdom of Bhutan | 0.69 (0.66-0.73) | 0.46 (0.43-0.48) | 2.31 (2.27-2.35) | 0.92 (0.88-0.95) |
| Republic of the Congo | 0.34 (0.32-0.37) | 0.12 (0.11-0.14) | 1.49 (1.36-1.63) | 0.85 (0.8-0.9) |
| Central African Republic | 0.02 (-0.03-0.08) | -0.04 (-0.09-0.01) | 0.67 (0.55-0.8) | 0.22 (0.17-0.26) |
| Republic of Angola | 0.25 (0.19-0.31) | 0.03 (-0.02-0.08) | 1.98 (1.9-2.06) | 0.81 (0.75-0.88) |
| Islamic Republic of Pakistan | 0.58 (0.54-0.63) | 0.43 (0.4-0.47) | 1.65 (1.49-1.8) | 0.77 (0.74-0.8) |
| Republic of India | 0.59 (0.56-0.63) | 0.41 (0.38-0.45) | 2.12 (1.95-2.29) | 0.85 (0.8-0.91) |
| Federal Democratic Republic of Nepal | 0.64 (0.61-0.67) | 0.43 (0.39-0.46) | 2.19 (2-2.38) | 0.85 (0.81-0.89) |
| People's Republic of Bangladesh | 0.36 (0.32-0.41) | 0.1 (0.05-0.14) | 2.13 (2-2.25) | 0.65 (0.58-0.71) |
| Islamic Republic of Afghanistan | 1.29 (1.22-1.36) | 1.01 (0.95-1.07) | 3.36 (3.2-3.52) | 1.97 (1.95-1.99) |
| Republic of Mozambique | 0.14 (0.08-0.2) | 0.04 (-0.02-0.09) | 1.95 (1.87-2.03) | 0.61 (0.55-0.67) |
| Republic of Malawi | 0.09 (0.02-0.16) | -0.11 (-0.18--0.04) | 1.85 (1.74-1.97) | 0.57 (0.5-0.65) |
| Republic of Madagascar | 0.02 (-0.04-0.08) | -0.11 (-0.17--0.04) | 0.91 (0.79-1.04) | 0.28 (0.19-0.36) |
| Republic of Mauritius | 0.17 (0.11-0.23) | -0.15 (-0.21--0.09) | 25.37 (21.1-29.64) | 3.74 (3.25-4.22) |
| Federal Democratic Republic of Ethiopia | 0 (-0.08-0.08) | -0.37 (-0.45--0.3) | 0.92 (0.76-1.09) | 0.23 (0.13-0.33) |
| Republic of Kenya | 0.34 (0.29-0.4) | 0.31 (0.27-0.36) | 2.17 (2.12-2.22) | 0.92 (0.88-0.97) |
| Union of the Comoros | 0.22 (0.19-0.26) | 0.07 (0.03-0.1) | 1.52 (1.34-1.7) | 0.61 (0.54-0.68) |
| Republic of Djibouti | 0.23 (0.17-0.29) | 0.09 (0.05-0.14) | 1.47 (1.32-1.62) | 0.61 (0.58-0.64) |
| State of Eritrea | 0.3 (0.26-0.35) | 0.17 (0.12-0.22) | 2.05 (1.83-2.27) | 0.85 (0.8-0.89) |
| Republic of Burundi | 0.01 (-0.05-0.08) | -0.16 (-0.23--0.1) | 0.41 (0.27-0.55) | 0.11 (0.05-0.18) |
| Republic of Equatorial Guinea | 0.61 (0.57-0.64) | 0.16 (0.12-0.2) | 3.22 (2.95-3.5) | 1.58 (1.49-1.67) |
| Gabonese Republic | 0.33 (0.31-0.36) | 0.12 (0.1-0.14) | 1.28 (1.09-1.48) | 0.71 (0.63-0.79) |
| Republic of Zimbabwe | 0.06 (-0.02-0.13) | 0.22 (0.18-0.27) | 1.63 (1.21-2.05) | 0.06 (-0.02-0.13) |
| Republic of Botswana | 0.25 (0.18-0.32) | 0.08 (0.04-0.13) | 1.29 (0.84-1.75) | 0.44 (0.33-0.55) |
| Kingdom of Eswatini | 0.08 (0-0.17) | 0 (-0.07-0.06) | 1.87 (1.57-2.16) | 0.44 (0.41-0.48) |
| Republic of Namibia | 0.18 (0.1-0.26) | 0 (-0.07-0.06) | 1.34 (1.2-1.48) | 0.4 (0.33-0.46) |
| Republic of South Africa | 0.18 (0.11-0.26) | 0.06 (0.01-0.11) | 0.53 (0.38-0.67) | 0.24 (0.15-0.33) |
| Republic of Zambia | 0.21 (0.15-0.27) | -0.02 (-0.06-0.02) | 1.54 (1.39-1.69) | 0.63 (0.54-0.72) |
| Kingdom of Lesotho | 0.2 (0.12-0.28) | 0.23 (0.16-0.29) | 3.08 (2.68-3.48) | 0.67 (0.62-0.72) |
| Republic of Seychelles | 1.26 (1.14-1.39) | 0.89 (0.77-1.02) | 4.71 (3.71-5.71) | 2.26 (1.9-2.62) |
| United Republic of Tanzania | 0.16 (0.11-0.21) | 0.02 (-0.03-0.08) | 1.51 (1.46-1.56) | 0.55 (0.51-0.6) |
| Republic of Uganda | 0.12 (0.05-0.19) | -0.08 (-0.15--0.01) | 2.44 (2.21-2.67) | 0.7 (0.65-0.75) |
| Republic of Rwanda | 0.16 (0.1-0.21) | -0.19 (-0.24--0.13) | 0.58 (0.42-0.75) | 0.25 (0.17-0.34) |
| Federal Republic of Somalia | -0.03 (-0.11-0.04) | -0.11 (-0.19--0.03) | 0.5 (0.35-0.64) | 0.06 (0.01-0.11) |
| Republic of Mali | 0.28 (0.26-0.29) | 0.1 (0.08-0.13) | 1.49 (1.36-1.62) | 1.13 (1.03-1.23) |
| Republic of Liberia | 0.37 (0.35-0.39) | 0.11 (0.07-0.14) | 2.94 (2.71-3.16) | 2.21 (2.04-2.39) |
| Republic of Guinea-Bissau | 0.44 (0.42-0.46) | 0.27 (0.25-0.29) | 1.59 (1.53-1.65) | 1.3 (1.26-1.35) |
| Republic of Ghana | 2.01 (1.81-2.21) | 1.78 (1.6-1.96) | 1.02 (0.91-1.13) | 1.38 (1.3-1.47) |
| Republic of Guinea | 0.29 (0.26-0.32) | 0.15 (0.12-0.19) | 2.36 (2.31-2.42) | 1.7 (1.65-1.76) |
| Republic of Côte d'Ivoire | 0.29 (0.24-0.34) | 0.1 (0.06-0.15) | 2.4 (2.25-2.56) | 1.83 (1.7-1.96) |
| Republic of the Gambia | 0.62 (0.58-0.66) | 0.5 (0.46-0.54) | 2.63 (2.35-2.91) | 2.05 (1.84-2.26) |
| Republic of Chad | 0.26 (0.21-0.3) | 0.14 (0.1-0.19) | 2.09 (2-2.18) | 1.37 (1.3-1.44) |
| Republic of Cameroon | 0.39 (0.36-0.43) | 0.23 (0.21-0.25) | 1.62 (1.5-1.74) | 1.31 (1.21-1.42) |
| Republic of Cabo Verde | 0.57 (0.54-0.6) | 0.37 (0.34-0.4) | 0.54 (0.31-0.77) | 0.55 (0.38-0.72) |
| Burkina Faso | 0.23 (0.21-0.26) | 0.11 (0.07-0.15) | 1.81 (1.54-2.09) | 1.28 (1.09-1.48) |
| Republic of Benin | 0.3 (0.27-0.32) | 0.12 (0.1-0.15) | 1.55 (1.42-1.68) | 1.16 (1.07-1.25) |
| Islamic Republic of Mauritania | 0.35 (0.34-0.37) | 0.07 (0.06-0.09) | 1.36 (1.24-1.48) | 1.06 (0.97-1.14) |
| Federal Republic of Nigeria | 0.77 (0.73-0.82) | 0.57 (0.53-0.61) | 2.02 (1.79-2.26) | 1.63 (1.46-1.8) |
| Republic of Senegal | 0.49 (0.47-0.51) | 0.34 (0.31-0.37) | 1.9 (1.72-2.08) | 1.51 (1.38-1.64) |
| Democratic Republic of Sao Tome and Principe | 0.49 (0.46-0.52) | 0.33 (0.29-0.36) | 2.46 (1.87-3.05) | 1.31 (1.08-1.55) |
| Republic of the Niger | 0.24 (0.21-0.27) | 0.06 (0.02-0.1) | 0.84 (0.66-1.02) | 0.6 (0.49-0.71) |
| Republic of Sierra Leone | 0.37 (0.33-0.42) | 0.24 (0.2-0.28) | 3.03 (2.86-3.2) | 2.15 (2.03-2.27) |
| Togolese Republic | 0.31 (0.27-0.35) | 0.15 (0.12-0.18) | 1.53 (1.34-1.72) | 1.23 (1.07-1.38) |
| American Samoa | 0.1 (0.05-0.15) | -0.17 (-0.22--0.12) | 1.73 (1.63-1.82) | 0.1 (0.05-0.16) |
| Bermuda | 0.8 (0.72-0.89) | 0.54 (0.49-0.6) | -1.03 (-1.27--0.79) | -0.4 (-0.54--0.26) |
| Cook Islands | 0.03 (-0.01-0.08) | -0.4 (-0.45--0.35) | 0.91 (0.74-1.08) | 0.04 (-0.01-0.08) |
| Greenland | -0.13 (-0.28-0.02) | -0.25 (-0.4--0.09) | -1.49 (-1.85--1.13) | -0.11 (-0.25-0.02) |
| Guam | 0.02 (-0.03-0.07) | -0.33 (-0.39--0.26) | 2.37 (0.95-3.79) | 0.04 (-0.02-0.09) |
| Principality of Monaco | 0.97 (0.91-1.03) | 0.86 (0.81-0.91) | 1.79 (1.44-2.14) | 1.31 (1.12-1.49) |
| Republic of Nauru | 0.16 (0.1-0.21) | -0.21 (-0.23--0.2) | 0.79 (0.56-1.01) | 0.16 (0.1-0.22) |
| Republic of Niue | 0.14 (0.11-0.16) | -0.25 (-0.26--0.23) | 1.64 (1.14-2.13) | 0.16 (0.12-0.19) |
| Northern Mariana Islands | 0.07 (0.04-0.1) | -0.15 (-0.18--0.13) | 0.08 (-0.33-0.48) | 0.07 (0.04-0.1) |
| Republic of Palau | 0.21 (0.19-0.24) | -0.1 (-0.12--0.08) | 2.51 (2.34-2.67) | 0.24 (0.21-0.26) |
| Puerto Rico | 1.01 (0.92-1.1) | 0.77 (0.7-0.84) | 0.22 (-0.05-0.48) | 0.34 (0.14-0.55) |
| Saint Kitts and Nevis | 0.85 (0.74-0.96) | 0.56 (0.49-0.64) | -0.42 (-0.79--0.04) | -0.5 (-0.83--0.18) |
| Tokelau | 0.24 (0.22-0.27) | -0.21 (-0.23--0.2) | 1.69 (1.1-2.28) | 0.26 (0.23-0.3) |
| Tuvalu | 0.17 (0.14-0.2) | -0.3 (-0.32--0.28) | 1.32 (1.24-1.41) | 0.18 (0.15-0.21) |
| Republic of San Marino | 0.29 (0.24-0.33) | 0.17 (0.11-0.24) | 0.34 (-0.02-0.69) | 0.28 (0.22-0.33) |
| United States Virgin Islands | 0.68 (0.62-0.74) | 0.47 (0.42-0.51) | 0.22 (0.08-0.37) | 0.49 (0.38-0.61) |
| Republic of South Sudan | 0 (-0.07-0.07) | -0.15 (-0.22--0.07) | 1.23 (1.16-1.31) | 0.32 (0.24-0.39) |
| Republic of Sudan | 1.34 (1.29-1.38) | 1.08 (1.03-1.13) | 3.74 (3.48-4.01) | 2.05 (2.01-2.09) |

Abbreviations: ASIR, age-standardized incidence rate; ASPR, age-standardized prevalence rate; ASMR, age-standardized mortality rate; ASDR, age-standardized disability rate; MS, Multiple sclerosis; SDI, socio-demographic index.

Supplementary Table 5 Global and regional prevalence of MS in 1990 and 2021, and EAPC of ASR from 1990 to 2021

| location | Case 1990(95%UI) | Case 2021(95%UI) | Percentage change | ASPR 1990(95%UI) | ASPR 2021(95%UI) | EAPC(95%UI) |
| --- | --- | --- | --- | --- | --- | --- |
| Global | 365303(307711.42-431148.96) | 606711.23(526386.61-695366.28) | 0.66 | 29.31(25.45-33.77) | 29.32(26.24-32.74) | 0.1 (0.06-0.14) |
| Low SDI | 9906.67(7750.14-12699.57) | 29346.09(23288.34-36488.49) | 1.96 | 8.79(7.16-10.61) | 10.4(8.58-12.26) | 0.57 (0.52-0.62) |
| Low-middle SDI | 27695.92(22053.15-35396.65) | 77240.51(61824.51-95687.93) | 1.79 | 10.04(8.21-12.06) | 13.94(11.62-16.57) | 1.13 (1.08-1.18) |
| Middle SDI | 37796.06(29886.55-47580.66) | 94725.24(76567.22-113824.76) | 1.51 | 8.76(7.32-10.39) | 12.53(10.58-14.74) | 1.33 (1.28-1.38) |
| High-middle SDI | 69148.52(58459-80537.42) | 103239.85(91837.25-116011.26) | 0.49 | 24.4(21.37-27.8) | 26.02(23.48-28.95) | 0.19 (0.16-0.22) |
| High SDI | 220312.32(187864.86-257272.66) | 301544.38(272233.7-333366.32) | 0.37 | 80.72(70.68-93) | 96.02(87.63-104.92) | 0.69 (0.63-0.75) |
| Andean Latin America | 689.18(525.64-902.91) | 2109.37(1648.4-2682.39) | 2.06 | 8.62(7.01-10.57) | 12.61(10.3-15) | 1.34 (1.28-1.41) |
| Australasia | 3373(2914.17-3910.25) | 7325.46(6138.24-8719.92) | 1.17 | 54.18(48.05-61.37) | 82.09(70.64-95) | 1.45 (1.14-1.75) |
| Caribbean | 1225.62(969.49-1518.57) | 2207.53(1770.02-2663.71) | 0.80 | 13.23(10.89-15.75) | 16.58(13.85-19.56) | 0.76 (0.68-0.84) |
| Central Asia | 2788.14(2231.97-3541.12) | 5313.93(4314.41-6640.64) | 0.91 | 29.13(25.49-32.99) | 29.54(26.12-33.69) | 0.08 (0.04-0.11) |
| Central Europe | 17940.84(15064.37-21110.3) | 19980.89(17752.89-22207.69) | 0.11 | 45.14(39.79-51.22) | 51.78(46.78-56.77) | 0.42 (0.34-0.5) |
| Central Latin America | 3480.43(2718.14-4445.6) | 10521.77(8375.1-12859.99) | 2.02 | 9.2(7.51-11.12) | 14.14(11.76-16.82) | 1.46 (1.36-1.56) |
| Central Sub-Saharan Africa | 637.16(479.87-845.27) | 1828.59(1397.78-2409.25) | 1.87 | 4.93(3.84-6.18) | 5.35(4.28-6.64) | 0.24 (0.18-0.3) |
| East Asia | 8186.08(5954.41-11397.2) | 12964.59(9741.82-17334.78) | 0.58 | 1.83(1.4-2.37) | 2.72(2.13-3.44) | 1.04 (0.87-1.22) |
| Eastern Europe | 20022.65(16293.26-24230.74) | 25334.98(22992.16-27753.92) | 0.27 | 27.35(23.39-31.92) | 34.62(31.76-37.75) | 0.67 (0.6-0.75) |
| Eastern Sub-Saharan Africa | 2471.94(1872.57-3291.73) | 6419.55(4936.38-8357.71) | 1.60 | 5.69(4.51-7.09) | 5.95(4.79-7.27) | 0.09 (0.03-0.16) |
| High-income Asia Pacific | 5177.99(4033.16-6626.55) | 5547.34(4368.73-7010.06) | 0.07 | 9.89(8.02-12.1) | 11.71(9.61-14.19) | 0.49 (0.43-0.56) |
| High-income North America | 120965.06(102978.13-141597.85) | 154160.15(142274.27-167033.62) | 0.27 | 134.59(117.65-155.36) | 145.65(135.61-156.47) | 0.52 (0.44-0.6) |
| North Africa and Middle East | 33072.59(27873.89-39070.95) | 105835.24(89221.55-125353.33) | 2.20 | 45.68(39.71-52.58) | 61.73(53.46-71) | 1.17 (1.11-1.22) |
| Oceania | 38.93(28.19-54.13) | 90.67(65.76-125.65) | 1.33 | 1.93(1.46-2.49) | 1.94(1.48-2.53) | -0.04 (-0.1-0.01) |
| South Asia | 24058.45(18924.13-30820.25) | 58687.24(46283.71-72920.61) | 1.44 | 8.84(7.19-10.76) | 10.42(8.58-12.43) | 0.58 (0.55-0.62) |
| Southeast Asia | 3887.28(2840.55-5375.82) | 6897.2(5162.62-9283.41) | 0.77 | 2.59(2-3.31) | 2.82(2.2-3.58) | 0.23 (0.18-0.28) |
| Southern Latin America | 3504.37(2804.4-4251.06) | 5602.2(4517.07-6754.73) | 0.60 | 24.87(20.51-29.48) | 26.18(21.6-30.89) | 0.15 (0.13-0.17) |
| Southern Sub-Saharan Africa | 1086.56(843.42-1410.02) | 2104.29(1659.34-2684.92) | 0.94 | 8.36(6.8-10.22) | 8.93(7.4-10.72) | 0.19 (0.12-0.26) |
| Tropical Latin America | 6634.17(5198.41-8467.57) | 14317.9(11345.67-17963.38) | 1.16 | 21.69(18.24-25.51) | 25.55(21.7-30.12) | 0.69 (0.58-0.8) |
| Western Europe | 102283.06(87677.85-119491.27) | 146232.19(127665.89-167455.73) | 0.43 | 88.16(76.98-101.58) | 121.71(108.47-137.09) | 1.08 (1.01-1.14) |
| Western Sub-Saharan Africa | 3779.48(2961.66-4831.25) | 13230.15(10618.2-16347.48) | 2.50 | 9.03(7.43-10.88) | 11.54(9.84-13.55) | 0.86 (0.82-0.91) |

Abbreviations: ASIR, age-standardized incidence rate; Multiple Sclerosis; SDI, sociodemographic index; GBD, Global Burden of Diseases, Injuries, and Risk Factors Study; EAPC, estimated annual percentage change; UIs, uncertainty intervals; CI, confidence interval.

Supplementary Table 6 Global and regional Incidence of MS in 1990 and 2021, and EAPC of ASR from 1990 to 2021

| location | Case 1990(95%UI) | Case 2021(95%UI) | Percentage change | ASIR 1990(95%UI) | ASIR 2021(95%UI) | EAPC(95%UI) |
| --- | --- | --- | --- | --- | --- | --- |
| Global | 22866.69(19661.09-26640.68) | 33939.85(29760.56-38684.01) | 0.48 | 1(0.88-1.14) | 0.98(0.88-1.1) | 0.04 (-0.02-0.1) |
| Low SDI | 744.41(596.59-934.97) | 2137.6(1744.75-2590.61) | 1.87 | 0.44(0.36-0.53) | 0.49(0.42-0.58) | 0.38 (0.35-0.42) |
| Low-middle SDI | 2053.13(1647.54-2556.27) | 5194.31(4253.23-6300.76) | 1.53 | 0.49(0.41-0.59) | 0.63(0.53-0.73) | 0.84 (0.8-0.89) |
| Middle SDI | 2986.55(2406.85-3692.44) | 5980.16(4951.83-7158.83) | 1.00 | 0.46(0.39-0.54) | 0.58(0.5-0.67) | 0.89 (0.85-0.93) |
| High-middle SDI | 4459.73(3847.56-5128.54) | 5016.54(4480.19-5632.99) | 0.12 | 0.96(0.85-1.07) | 0.96(0.88-1.06) | 0 (-0.05-0.06) |
| High SDI | 12594.44(11086.47-14401.9) | 15579.44(14176.55-17093.99) | 0.24 | 2.95(2.64-3.34) | 3.45(3.17-3.77) | 0.61 (0.57-0.64) |
| Andean Latin America | 55.09(42.99-70.4) | 154.79(122.2-190.03) | 1.81 | 0.41(0.34-0.49) | 0.55(0.46-0.66) | 1.06 (1-1.11) |
| Australasia | 207.51(183.64-233.81) | 412.81(357.26-474.94) | 0.99 | 2.07(1.86-2.33) | 2.95(2.59-3.35) | 1.24 (0.99-1.49) |
| Caribbean | 92.43(73.83-113.56) | 145.84(119.74-172.54) | 0.58 | 0.62(0.51-0.73) | 0.71(0.6-0.82) | 0.43 (0.39-0.48) |
| Central Asia | 290.64(242.94-352.74) | 491.19(422.14-579.07) | 0.69 | 1.45(1.27-1.64) | 1.32(1.17-1.5) | -0.38 (-0.42--0.33) |
| Central Europe | 1147.58(1003.18-1298.45) | 905.44(812.77-1002.7) | -0.21 | 2.08(1.88-2.32) | 2.02(1.86-2.21) | -0.14 (-0.18--0.1) |
| Central Latin America | 276.48(217.84-346.54) | 712.99(581.09-855.56) | 1.58 | 0.45(0.36-0.53) | 0.63(0.53-0.73) | 1.16 (1.07-1.24) |
| Central Sub-Saharan Africa | 49.31(38.11-64.02) | 135.88(107.13-172.17) | 1.76 | 0.28(0.23-0.34) | 0.28(0.23-0.34) | 0.07 (0.02-0.11) |
| East Asia | 759.17(564.54-1027.26) | 743.59(561.68-983.35) | -0.02 | 0.21(0.17-0.25) | 0.18(0.14-0.21) | -0.69 (-0.83--0.56) |
| Eastern Europe | 1416.67(1184.04-1651.55) | 1122.27(996.98-1259.34) | -0.21 | 1.41(1.23-1.61) | 1.34(1.23-1.47) | -0.21 (-0.26--0.16) |
| Eastern Sub-Saharan Africa | 193(149.7-250.43) | 478.58(377.82-602.12) | 1.48 | 0.31(0.25-0.38) | 0.31(0.25-0.37) | -0.1 (-0.17--0.04) |
| High-income Asia Pacific | 299.62(237.29-376.93) | 281.06(222.86-351.44) | -0.06 | 0.42(0.34-0.51) | 0.46(0.38-0.56) | 0.27 (0.22-0.32) |
| High-income North America | 6801.22(5913.99-7809.94) | 7898.96(7308.06-8528.45) | 0.16 | 4.71(4.16-5.39) | 4.94(4.6-5.34) | 0.42 (0.34-0.5) |
| North Africa and Middle East | 2325.13(1963.44-2730.64) | 6082.95(5180.76-7136.9) | 1.62 | 1.67(1.46-1.91) | 2.1(1.82-2.42) | 0.91 (0.84-0.97) |
| Oceania | 3.41(2.53-4.63) | 7.32(5.49-9.88) | 1.15 | 0.18(0.14-0.22) | 0.17(0.13-0.21) | -0.28 (-0.33--0.23) |
| South Asia | 1744.45(1397.62-2202.27) | 3949.53(3199.23-4812.96) | 1.26 | 0.45(0.37-0.53) | 0.5(0.42-0.59) | 0.41 (0.37-0.44) |
| Southeast Asia | 304.73(231.68-406.99) | 458.89(350.28-597.73) | 0.51 | 0.21(0.17-0.25) | 0.2(0.16-0.24) | -0.17 (-0.21--0.12) |
| Southern Latin America | 216.67(177.71-258.66) | 315.86(261.6-383.07) | 0.46 | 0.97(0.82-1.15) | 0.99(0.84-1.18) | 0.07 (0.05-0.09) |
| Southern Sub-Saharan Africa | 79.62(63.25-101.32) | 139.54(111.44-173.99) | 0.75 | 0.41(0.33-0.49) | 0.42(0.35-0.49) | 0.08 (0.04-0.13) |
| Tropical Latin America | 567.58(455.69-695.47) | 1082.4(886.46-1322.33) | 0.91 | 0.98(0.82-1.13) | 1.06(0.9-1.22) | 0.37 (0.29-0.45) |
| Western Europe | 5723.99(5015.3-6501.02) | 7343.94(6528.16-8244.75) | 0.28 | 3.27(2.92-3.68) | 4.34(3.9-4.85) | 0.94 (0.91-0.97) |
| Western Sub-Saharan Africa | 312.4(250.67-387.97) | 1076(886.85-1291.28) | 2.44 | 0.49(0.41-0.58) | 0.59(0.51-0.68) | 0.65 (0.62-0.67) |

Abbreviations: ASIR, age-standardized incidence rate; Multiple Sclerosis; SDI, sociodemographic index; GBD, Global Burden of Diseases, Injuries, and Risk Factors Study; EAPC, estimated annual percentage change; UIs, uncertainty intervals; CI, confidence interval.

Supplementary Table 7 Global and regional Deaths of MS in 1990 and 2021, and EAPC of ASR from 1990 to 2021

| location | Case 1990(95%UI) | Case 2021(95%UI) | Percentage change | ASIR 1990(95%UI) | ASIR 2021(95%UI) | EAPC(95%UI) |
| --- | --- | --- | --- | --- | --- | --- |
| Global | 1797.77(1698.82-1896.81) | 2111.99(1934.47-2320.57) | 0.17 | 0.25(0.24-0.26) | 0.23(0.21-0.24) | -0.35 (-0.46--0.24) |
| Low SDI | 46.49(17.73-77.18) | 218.53(110.04-330.57) | 3.73 | 0.03(0.01-0.05) | 0.05(0.03-0.08) | 2.05 (1.98-2.12) |
| Low-middle SDI | 62.22(39.54-87.92) | 271.62(207.26-356.58) | -0.81 | 0.03(0.02-0.04) | 0.05(0.04-0.06) | 2.26 (2.15-2.37) |
| Middle SDI | 132.63(108.61-160.41) | 427.72(376.92-486.87) | -1.00 | 0.05(0.04-0.05) | 0.08(0.07-0.08) | 1.57 (1.32-1.83) |
| High-middle SDI | 647.48(621.15-680.49) | 462.07(405.19-522.26) | -0.97 | 0.29(0.28-0.31) | 0.18(0.16-0.2) | -2.03 (-2.21--1.85) |
| High SDI | 903.66(883.24-926.7) | 728.73(694.88-763.06) | -0.57 | 0.63(0.61-0.64) | 0.69(0.64-0.73) | 0.43 (0.29-0.57) |
| Andean Latin America | 2.82(2.02-3.99) | 11.07(7.36-16.49) | 6.00 | 0.06(0.05-0.08) | 0.12(0.08-0.15) | 2.89 (2.48-3.29) |
| Australasia | 14.14(12.7-15.72) | 21.28(18.66-24.18) | 7.53 | 0.53(0.47-0.59) | 0.58(0.5-0.66) | 0.14 (-0.06-0.35) |
| Caribbean | 15.5(14.02-17.65) | 23.47(18.58-30.28) | -0.30 | 0.22(0.2-0.25) | 0.25(0.22-0.3) | 0.71 (0.55-0.87) |
| Central Asia | 14.14(12.54-15.96) | 12.69(9.55-16.22) | 20.27 | 0.22(0.19-0.25) | 0.13(0.1-0.17) | -0.64 (-1.3-0.02) |
| Central Europe | 271.84(257.97-289.48) | 125.83(109.98-143.77) | -0.91 | 0.96(0.91-1.02) | 0.64(0.57-0.72) | -1.41 (-1.47--1.36) |
| Central Latin America | 32.21(30.92-33.64) | 120.55(100.17-140.25) | -0.66 | 0.12(0.12-0.13) | 0.23(0.2-0.26) | 2.13 (1.75-2.5) |
| Central Sub-Saharan Africa | 0.97(0.41-1.78) | 4.32(2.16-7.31) | 54.73 | 0.02(0.01-0.03) | 0.03(0.01-0.04) | 1.56 (1.41-1.71) |
| East Asia | 19.15(11.49-29.69) | 26.69(19.33-35.37) | 2.90 | 0.01(0-0.01) | 0.01(0.01-0.01) | -0.21 (-0.8-0.39) |
| Eastern Europe | 420.72(407.05-435.77) | 220.13(181.13-267.77) | -0.48 | 0.59(0.57-0.61) | 0.37(0.32-0.44) | -2.68 (-3.07--2.29) |
| Eastern Sub-Saharan Africa | 3.44(1.2-5.91) | 14.03(5.99-21.44) | 6.76 | 0.02(0-0.03) | 0.03(0.01-0.04) | 1.44 (1.36-1.52) |
| High-income Asia Pacific | 13.18(12.29-14.13) | 12.01(10.89-13.32) | 25.03 | 0.04(0.04-0.04) | 0.04(0.03-0.04) | -0.25 (-0.58-0.08) |
| High-income North America | 328.35(317.9-339.01) | 300.78(286.27-316.19) | -0.10 | 0.68(0.65-0.7) | 0.99(0.92-1.05) | 1.09 (0.74-1.44) |
| North Africa and Middle East | 63.49(37.4-99.86) | 294.55(247.54-351.74) | 5.74 | 0.12(0.06-0.19) | 0.23(0.19-0.26) | 2.39 (2.2-2.57) |
| Oceania | 0(0-0) | 0(0-0.01) | Inf | 0(0-0) | 0(0-0) | 0.94 (0.79-1.08) |
| South Asia | 18.21(8.97-31.77) | 74.62(51.72-98.74) | 5.91 | 0.01(0.01-0.02) | 0.02(0.02-0.03) | 2.05 (1.92-2.19) |
| Southeast Asia | 10.48(7.18-14.1) | 32.41(26.67-38.77) | 2.09 | 0.01(0.01-0.01) | 0.02(0.01-0.02) | 1.65 (1.43-1.88) |
| Southern Latin America | 22.3(20.43-24.45) | 19.73(17.19-22.36) | 19.72 | 0.27(0.25-0.29) | 0.17(0.15-0.19) | -1.69 (-1.96--1.42) |
| Southern Sub-Saharan Africa | 5.95(4.47-7.4) | 10.81(7.45-14.72) | 1.36 | 0.09(0.06-0.12) | 0.12(0.09-0.15) | 0.68 (0.52-0.83) |
| Tropical Latin America | 22.25(21.11-23.5) | 54.06(50.01-58.41) | 11.21 | 0.12(0.11-0.13) | 0.15(0.14-0.16) | -0.06 (-0.63-0.51) |
| Western Europe | 443.52(426.19-463.61) | 389.81(365.1-414.06) | -0.97 | 0.75(0.72-0.77) | 0.86(0.78-0.91) | 0.81 (0.71-0.92) |
| Western Sub-Saharan Africa | 75.11(39.76-113.39) | 343.14(209.32-498.55) | 8.70 | 0.07(0.04-0.1) | 0.12(0.07-0.17) | 1.8 (1.66-1.95) |

Abbreviations: ASIR, age-standardized incidence rate; Multiple Sclerosis; SDI, sociodemographic index; GBD, Global Burden of Diseases, Injuries, and Risk Factors Study; EAPC, estimated annual percentage change; UIs, uncertainty intervals; CI, confidence interval.

Supplementary Table 8 Global and regional DALYs of MS in 1990 and 2021, and EAPC of ASR from 1990 to 2021

| location | Case 1990(95%UI) | Case 2021(95%UI) | Percentage change | ASDR 1990(95%UI) | ASDR 2021(95%UI) | EAPC(95%UI) |
| --- | --- | --- | --- | --- | --- | --- |
| Global | 187147.57(157391.96-222277.19) | 269173.6(221179.06-325225.96) | -0.70 | 15.85(13.65-18.37) | 14.54(12.37-16.93) | -0.3 (-0.37--0.23) |
| Low SDI | 5760.13(3521.16-8195.12) | 22132.76(14330.76-30372.96) | -0.55 | 3.88(2.61-5.41) | 5.6(3.95-7.46) | 1.2 (1.13-1.27) |
| Low-middle SDI | 11439.39(8522.11-15401.52) | 37299.28(28831.56-46892.01) | 3.33 | 3.78(2.86-5.02) | 5.98(4.8-7.56) | 1.54 (1.52-1.56) |
| Middle SDI | 17467.57(14163.62-22304.11) | 47763.16(39069.92-58353.17) | -0.88 | 3.94(3.23-4.89) | 6.15(5.11-7.4) | 1.52 (1.43-1.62) |
| High-middle SDI | 51084.66(45437.83-58094.15) | 49540.6(40813.8-58389.88) | -1.00 | 17(15.22-19.19) | 12.86(10.89-14.86) | -1.27 (-1.39--1.14) |
| High SDI | 101013.86(84099.42-120027.72) | 112113.84(90051.39-135682.95) | -0.53 | 41.52(35.63-47.96) | 44.54(37.45-51.42) | 0.32 (0.22-0.42) |
| Andean Latin America | 338.53(264.83-445.84) | 1141.37(859.65-1502.09) | 64.38 | 4.41(3.55-5.61) | 7.21(5.75-8.98) | 1.98 (1.76-2.2) |
| Australasia | 1568.54(1264.5-1942.3) | 2885.87(2208.76-3650.42) | 0.58 | 30.47(25.88-35.61) | 38.08(30.85-45.73) | 0.69 (0.45-0.93) |
| Caribbean | 1151.8(1014.97-1331.42) | 1799.07(1470.64-2235.24) | -0.03 | 11.98(10.64-13.9) | 13.76(11.68-16.49) | 0.59 (0.48-0.69) |
| Central Asia | 1513.4(1240.79-1837.69) | 2089.35(1502.34-2712.46) | 6.47 | 12.95(10.71-15.41) | 10.57(8.19-13.15) | -0.4 (-0.58--0.21) |
| Central Europe | 18245.46(16563.45-20150.96) | 11306.29(9685.76-13113.04) | -0.84 | 48.12(44.27-52.71) | 35.47(30.99-40.31) | -1.06 (-1.09--1.03) |
| Central Latin America | 2693.54(2348.61-3105.95) | 9025.49(7598.5-10525.73) | 40.62 | 6.93(6.09-7.95) | 12.14(10.33-14) | 1.87 (1.59-2.15) |
| Central Sub-Saharan Africa | 230.94(162.21-330.57) | 745.01(534.47-1046.71) | 160.51 | 1.95(1.4-2.74) | 2.43(1.79-3.29) | 0.67 (0.57-0.76) |
| East Asia | 3340.17(2410.58-4769.29) | 5021.64(3549.56-6727.73) | -0.46 | 0.81(0.6-1.15) | 1.08(0.79-1.4) | 0.58 (0.4-0.75) |
| Eastern Europe | 26782.88(24740.56-29040.39) | 17467.8(14613.21-20169.95) | 0.57 | 32.1(29.56-34.73) | 23.71(20.25-27.31) | -1.89 (-2.2--1.58) |
| Eastern Sub-Saharan Africa | 883.58(613.14-1263.26) | 2569.34(1780.61-3536.41) | 29.46 | 2.14(1.48-3.03) | 2.54(1.75-3.38) | 0.46 (0.39-0.53) |
| High-income Asia Pacific | 2126.86(1620.85-2777.03) | 2151.1(1603.23-2808.74) | 8.60 | 4.22(3.34-5.38) | 4.57(3.49-5.9) | 0.24 (0.1-0.38) |
| High-income North America | 47018.48(37841.19-57554.64) | 53543.09(42526.98-65163.16) | -0.96 | 56.94(47.05-67.44) | 64.29(54.04-74.71) | 0.46 (0.28-0.64) |
| North Africa and Middle East | 12004.6(9172.23-16098.63) | 42139.31(33378.54-52327.7) | -0.25 | 15.9(12.3-21.04) | 23.4(18.99-28.51) | 1.47 (1.4-1.53) |
| Oceania | 11.03(6.73-16.91) | 25.72(15.89-39.29) | 1,582.66 | 0.55(0.36-0.79) | 0.55(0.36-0.8) | -0.04 (-0.09-0.02) |
| South Asia | 7742.92(5366.07-10867.85) | 20421.49(14893.51-27428.76) | -0.15 | 2.92(2.11-4.01) | 3.74(2.81-4.91) | 0.84 (0.79-0.88) |
| Southeast Asia | 1685.65(1239.81-2255.35) | 3682.85(2918-4577.72) | 1.18 | 1.14(0.86-1.51) | 1.52(1.22-1.85) | 0.82 (0.75-0.9) |
| Southern Latin America | 2073.03(1738.58-2506.59) | 2479.67(1936.14-3110.74) | 24.83 | 16.04(13.85-18.84) | 12.7(10.31-15.24) | -0.87 (-1.01--0.74) |
| Southern Sub-Saharan Africa | 614.52(487.24-781.3) | 1113.51(851.38-1434.46) | 437.02 | 5.5(4.23-6.91) | 6.26(5.11-7.72) | 0.32 (0.24-0.41) |
| Tropical Latin America | 2972.13(2337.04-3791) | 6561.82(5247.56-8101.56) | -0.62 | 9.32(7.55-11.51) | 11.28(9.14-13.61) | 0.4 (0.16-0.64) |
| Western Europe | 48008.22(39668.94-57522.67) | 56094.21(44458.59-68078.15) | -0.90 | 47.21(40.44-54.5) | 56.53(47.25-65.46) | 0.77 (0.7-0.83) |
| Western Sub-Saharan Africa | 6141.27(3677.27-8770.48) | 26909.6(17714.7-37822.09) | -0.88 | 7.01(4.66-9.58) | 10.99(7.78-14.82) | 1.48 (1.4-1.57) |

Abbreviations: ASIR, age-standardized incidence rate; Multiple Sclerosis; SDI, sociodemographic index; GBD, Global Burden of Diseases, Injuries, and Risk Factors Study; EAPC, estimated annual percentage change; UIs, uncertainty intervals; CI, confidence interval.

Supplementary Table9. The EAPC in Disease Burden of MS in Women of Childbearing Age by Age Group from 1990 to 2021

| Metric | age | Prevalence(95%UI) | Incidence(95%UI) | Deaths(95%UI) | DALYs(95%UI) |
| --- | --- | --- | --- | --- | --- |
| Number | 15-19 years | 11507.3(7798.08-15676.15) | 2755.94(1779.19-3931.4) | 17.31(14.05-20.98) | 4497.92(3118.56-6383.94) |
|  | 20-24 years | 30361.8(22386.63-40651.83) | 4896.26(3360.59-6741.32) | 388.1(252.48-545.85) | 34611.93(24396.56-45775.23) |
|  | 25-29 years | 60308.41(46458.27-75872.75) | 6821.17(4734.1-9070.61) | 87.88(79.02-97.89) | 21616.94(15584.03-28479.79) |
|  | 30-34 years | 99237.34(80045.32-118260.15) | 7563.59(5212.63-10281.43) | 188.43(175.59-205.11) | 37006.26(28347.49-47222.47) |
|  | 35-39 years | 128282.38(108821.22-150288.5) | 5790.4(3730.31-7965.84) | 312.19(288.42-340.27) | 49955.2(39965.67-61121.5) |
|  | 40-44 years | 138239.57(122219.39-158738.69) | 3679.07(2235.12-5463) | 464.88(431.76-507.25) | 57904.44(46799.6-69141.43) |
|  | 45-49 years | 138774.44(123534.35-156750.91) | 2433.42(1615.57-3416.69) | 653.2(611.76-696.65) | 63580.91(53117.72-74776.63) |
| Rate | 15-19 years | 3.79(2.57-5.16) | 0.91(0.59-1.29) | 0.01(0-0.01) | 1.48(1.03-2.1) |
|  | 20-24 years | 10.34(7.62-13.84) | 1.67(1.14-2.29) | 0.13(0.09-0.19) | 11.78(8.31-15.58) |
|  | 25-29 years | 20.73(15.97-26.07) | 2.34(1.63-3.12) | 0.03(0.03-0.03) | 7.43(5.36-9.79) |
|  | 30-34 years | 33.2(26.78-39.56) | 2.53(1.74-3.44) | 0.06(0.06-0.07) | 12.38(9.48-15.8) |
|  | 35-39 years | 46.18(39.17-54.1) | 2.08(1.34-2.87) | 0.11(0.1-0.12) | 17.98(14.39-22) |
|  | 40-44 years | 55.72(49.26-63.98) | 1.48(0.9-2.2) | 0.19(0.17-0.2) | 23.34(18.86-27.87) |
|  | 45-49 years | 58.89(52.42-66.52) | 1.03(0.69-1.45) | 0.28(0.26-0.3) | 26.98(22.54-31.73) |
| *MS, Multiple sclerosis; EAPC, Estimated Annual Percentage Change****.*** | | | | | |
