# Supplementary figures and images for: The epidemiological trends of multiple sclerosis among women of child-bearing age: a global analysis from 1990 to 2021 and forecasts to 2040
Source: Front Immunol. 2026 May 1;17:1677178. doi: 10.3389/fimmu.2026.1677178 (PMC13175834; doi:10.3389/fimmu.2026.1677178)

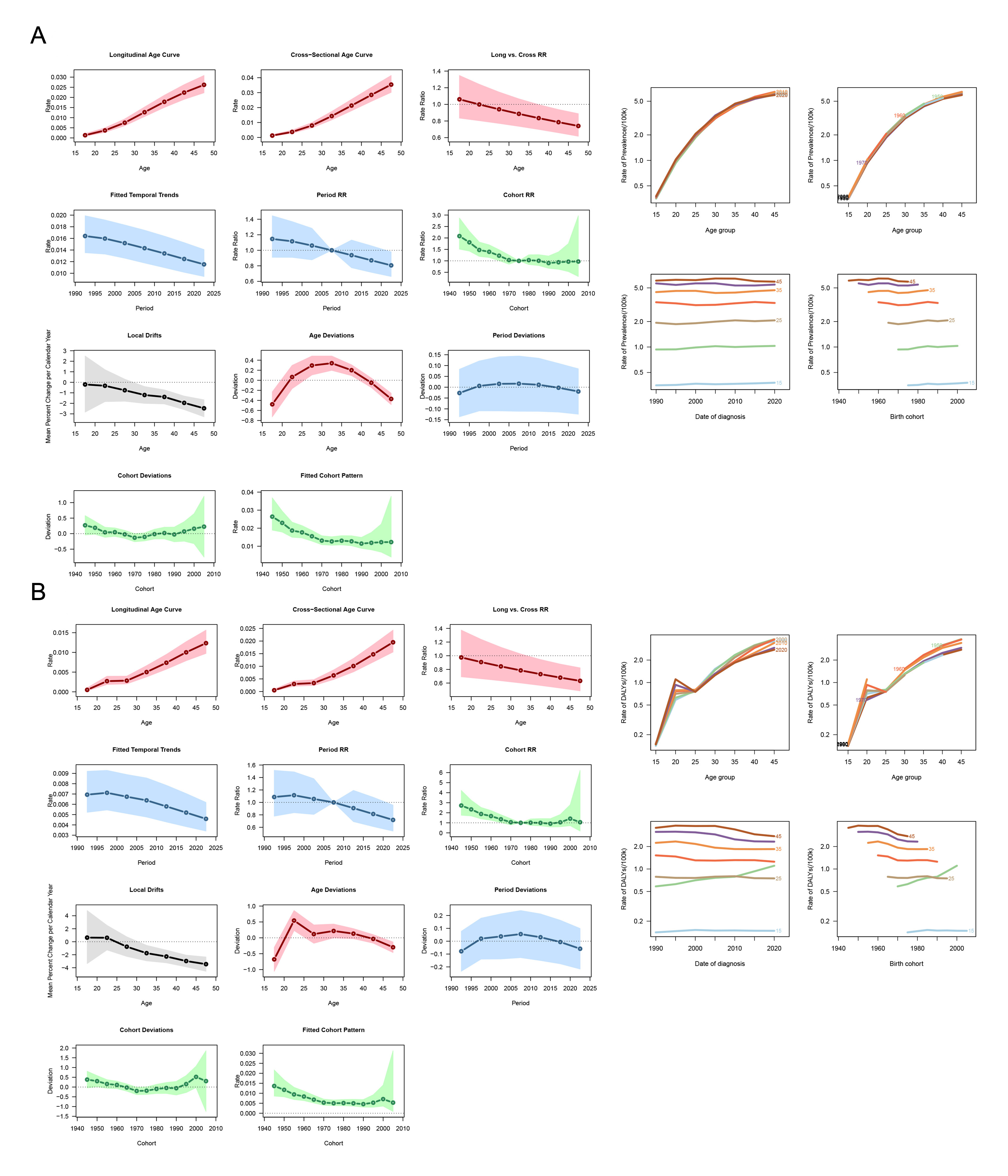

Supplement: Supplementary Figure 1 — Age, period, and cohort trends in global MS among women of childbearing age. (A) prevalence (B) DALYs. [file Image1.jpeg]
